# Stereoselective Synthesis of C‑Glycosylated Pyrrolizidines through Nitrone Cycloadditions

**DOI:** 10.1021/acsomega.5c10431

**Published:** 2026-01-07

**Authors:** Francisco Franco-Montalban, Mónica Díaz-Gavilán, Daniele Lo Re, Juan A. Tamayo

**Affiliations:** Department of Medicinal and Organic Chemistry, Faculty of Pharmacy, 16741 University of Granada, Campus de Cartuja S/N, Granada 18071, Spain

## Abstract

Polyhydroxylated alkaloids, among which iminosugars represent a prominent subclass, are a structurally diverse family of carbohydrate mimics with significant biological activity. Herein, we report a concise and stereoselective synthesis of five novel C-glycosylated pyrrolizidines (**15**–**19**) related to casuarine and hyacinthacines, achieved through 1,3-dipolar cycloadditions of nitrones with *E*-alkenes. The reactions of nitrone **21** with fructose-derived α,β-unsaturated ester **20a** and ketone **20b** proceeded with predictable anti-selectivity, affording isoxazolidine intermediates that were transformed into pyrrolizidines via reductive N–O cleavage and subsequent ring closure. Careful stereochemical assignments were established by NOE analyses.

## Graphical abstract

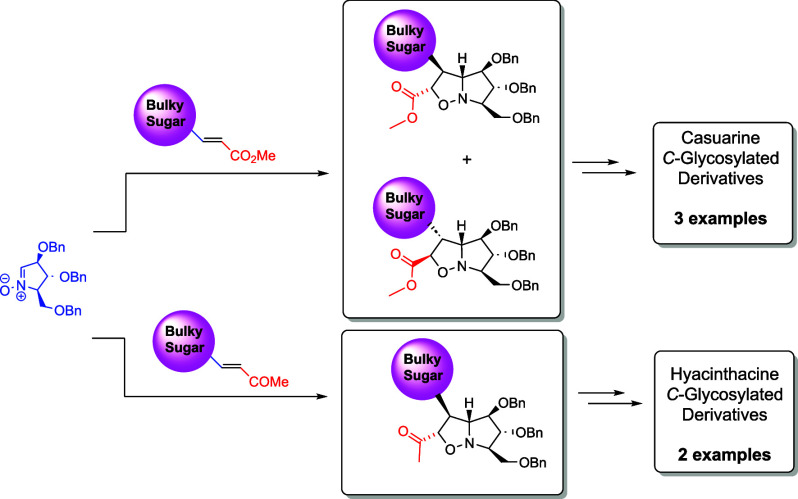

## Introduction

Polyhydroxylated alkaloids represent a widely distributed group of natural compounds, typically isolated from the water-soluble fractions of medicinal plants as well as other organisms.^1–5^ Within this family, *iminosugars* (also known as *azasugars*) constitute the best-studied subgroup and are commonly categorized into five structural classes: pyrrolidines, piperidines, pyrrolizidines (Figure 1), indolizidines, and nortropanes. Members within each class differ primarily in their polyhydroxylation patterns.

**Figure 1:**
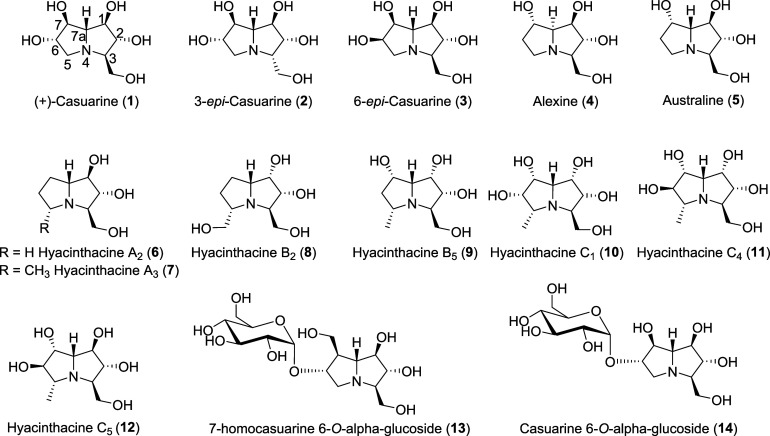
Examples of natural polyhydroxylated pyrrolizidine alkaloids.

As carbohydrate mimics, many polyhydroxylated alkaloids exhibit biological activity, and some glycoside-processing enzymes can recognize and interact with them.^6–10^ Since the etiology of various diseases, including cancer, HIV, and diabetes, is mediated by these enzymes, polyhydroxylated alkaloids have attracted considerable attention as antiviral, antitumoral, antidiabetic, immunostimulatory, and anti-inflammatory agents, as well as for the treatment of lysosomal storage diseases, among others.^3,5,11^

Natural alkyl-substituted polyhydroxylated pyrrolizidines are structurally limited alkaloids. Apart from hydroxymethyl groups at C-3 and/or C-5, other naturally occurring alkyl substituents are mostly limited to a methyl group at C-5.^2,12–14^ Natural and synthetic pyrrolizidine *O*-glycosides are well documented^15–19^ Their disaccharide-like structures account for interesting inhibitory activities with higher enzyme selectivity.^17,18^ In contrast, only a few alkylpolyhydroxylated pyrrolizidines with alternative substitution patterns have been reported,^20,21^ and no examples are known where the iminosugar is directly attached to the anomeric carbon of a saccharide moiety via a C-glycosidic bond.^22^ This gap is relevant because the stability of C-glycosidic linkages makes iminosugars attractive targets for synthetic modification, particularly C-glycosylation, to probe new biological activities and bond stability.

Among the various approaches reported for pyrrolizidine synthesis,^22^ 1,3-dipolar cycloadditions (1,3DC) of suitable alkenes and nitrones^23–32^ have proven particularly effective, enabling access to pyrrolizidines^33,34^ via isoxazolidine intermediates. This well-studied strategy^34–42^ allows the formation of multiple stereocenters with high regio- and stereoselectivity in a step-efficient, noncatalyzed process. For substituted five-membered cyclic nitrones, stereocontrol is guided by the nitrone configuration, with diastereoselectivity influenced by steric demands of both partners.^34,43^ This predictable stereocontrol makes 1,3DC an ideal platform for the preparation of novel C-glycosylated pyrrolizidines, where precise stereochemistry is key to achieving biologically relevant enzyme inhibition and controlling the structural stability of the resulting glycosidic linkages.

Given the promising biological activity of pyrrolizidine *O*-glycosides, we pursued the preparation of *C*-glycosylated casuarine (**15**–**17**) and hyacinthacine (**18**–**19**) derivatives (Figure 2) using a 1,3-dipolar cycloaddition strategy between fructose-derived *E*-alkenes **20a** and **20b** and the well-known nitrone **21** (Figure 3).

**Figure 2:**
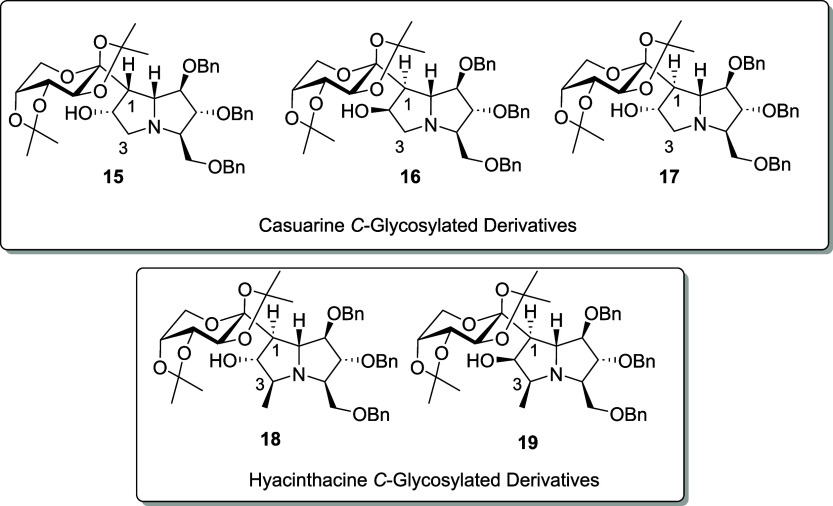
*C*-glycosylated derivatives **15–19**.

**Figure 3:**
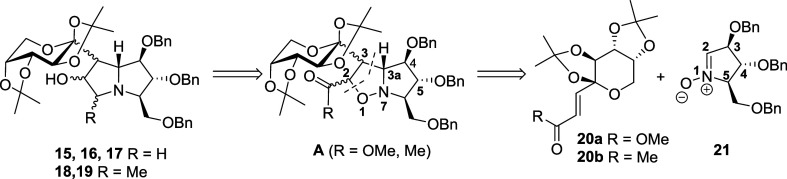
Retrosynthetic analysis for *C*-glycosylated casuarine (**15**–**17**) and hyacinthacine (**18**–**19**) derivatives.

## Results and Discussion

A retrosynthetic analysis of pyrrolizidines **15**–**19** suggested that these compounds could be accessed through isoxazolidine intermediate A (Figure 3). This key intermediate, in turn, was envisioned to arise from a 1,3-dipolar cycloaddition between an α,β-unsaturated ester or ketone (**20a** and **20b**, respectively) and a suitably protected nitrone **21** with a d-arabinose configuration. Within this synthetic approach, α,β-unsaturated ester and ketone **20a** and **20b** serve to introduce the saccharide moiety, which remains directly attached to the bicyclic core via a C–C bond in the final pyrrolizidine rings. Compounds **20a**,^44^ **20b**,^45^ and nitrone **21** ^46,47^ were synthesized according to procedures reported in the literature.

Nitrone **21** ^46,47^ has been extensively reported by several authors^17,40,42,43,46,48–52^ to undergo 1,3-dipolar cycloadditions with various types of alkenes, with the observed regio- and stereoselectivity depending on the alkene employed and being largely governed by both steric and electronic effects.^29^

Regarding stereoselectivity, nitrone **21** follows the general trend of five-membered cyclic nitrones, where the nitrone configuration governs the outcome of the cycloaddition.^16,17,29,38^ The benzyl protecting groups at positions 3 and 5 of the nitrone strongly enforce an *anti* approach of the dipolarophile to nitrone **21**, while the benzyl group at position 4 favors an *exo* approach over an *endo* approach in reactions with monosubstituted alkenes and *Z*-disubstituted alkenes. As a result, the observed diastereoselectivity reflects the combined effect of these steric constraints and the nature of the dipolarophile.

The cycloaddition between the α,β-unsaturated ester **20a** ^44^ and nitrone **21** was first explored under various conditions. When carried out in dichloromethane at 70 °C for 2.5 h under microwave irradiation, the reaction afforded *anti-exo* **22a** and *anti-endo* **22b** products in a 1:1.2 ratio with an overall yield of 54%. Importantly, *anti* refers to the relative orientation of the nitrone C-3 and C-5 substituents with respect to the approaching alkene, while *endo* and *exo* denote the approach of the carbonyl substituent. Extending the reaction time to 30 h in a sealed tube under otherwise identical conditions improved the yield to 83% while maintaining the same isomer ratio. Similarly, the reaction of α,β-unsaturated methylketone **20b** ^45^ with nitrone **21** was investigated under microwave irradiation in dichloromethane at 70 °C. In contrast to the reaction with **20a**, which gave a mixture of stereoisomers, the cycloaddition with **20b** proceeded stereoselectively, affording a single stereomer under the same conditions. After 2.5 h, *anti*-*endo* cycloadduct **23** was obtained in 49% yield. Performing the same transformation in a sealed tube under identical solvent and temperature conditions but extending the reaction time to 18 h significantly increased the yield to 71%. These results highlight the strong influence of the substituent in *E*-alkenes. In the case of ester **20a**, the ester group in the *E*-alkene with the incoming sugar moiety leads to the formation of nearly equal amounts of the *exo* and *endo* products. In contrast, with ketone **20b**, only the *endo* isomer was obtained, with the sugar moiety adopting an *exo* orientation. This outcome underscores the key role of the substituents in controlling the stereoisomeric outcome of the cycloaddition with disubstituted *E*-alkenes (Scheme 1). It is noteworthy that the cycloaddition exhibited the opposite regioselectivity to that reported for a closely related system.^42^ This difference highlights the sensitivity of these reactions to structural and electronic variations and merits consideration in the interpretation of the reaction outcome (Figure 4).

**Scheme 1:**
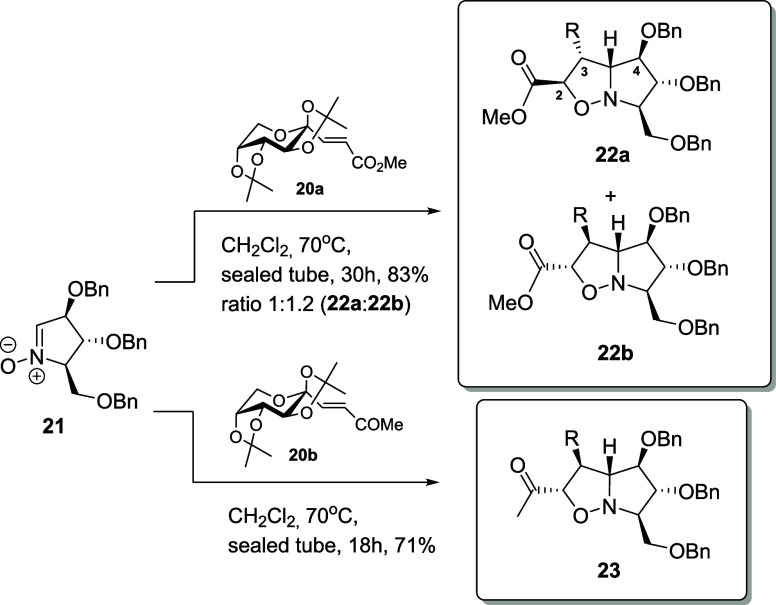
1,3-Dipolar Cycloaddition of Nitrone **21** with Alkenes **20a** and **20b**

**Figure 4:**
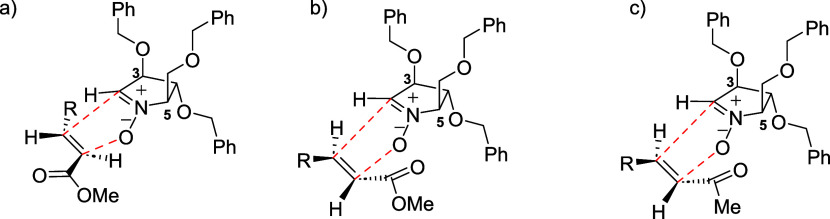
Transition state leading to (a) **22a**, (b) **22b**, and (c) **23**.

Extensive NOE experiments were performed on isoxazolidines **22a**, **22b,** and **23** to establish their stereochemical relationships. These analyses confirmed that the cycloaddition consistently occurs with an *anti* relationship between C-3 and C-5 of the nitrone and the incoming alkene, in line with the expected behavior of nitrone **21**,^16,17,29,38^ as the benzyl protecting groups at positions 3 and 5 strongly enforce an anti approach of the dipolarophile, minimizing steric interactions during the cycloaddition and leading to the observed anti C-3/C-5 relationship. Remarkably, this *anti* stereochemistry was observed across all of the examined products. For the reaction with ester **20a**, NOE correlations confirmed that compound **22a** adopts an *exoanti* orientation of the carboxylate group, as shown by interactions between H-2/H-4 and H-2/H-6, whereas compound **22b** displays an *endoanti* approach, as evidenced by the NOE values between H-3 and H-6. In the case of the cycloaddition with ketone **20b**, the NOE analysis confirmed that isoxazolidine **23** adopts an *endoanti* orientation of the acetyl group, as indicated by the correlation between H-3 and H-4. Figure 5 depicts the structures of **22a**, **22b**, and **23**, showing the NOE correlations used to assign their stereochemistry. NOE data for all other synthesized compounds are provided in the Supporting Information.

**Figure 5:**
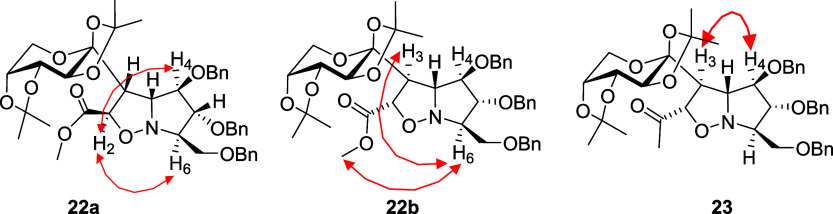
NOE interactions in compounds **22a**, **22b**, and **23**.

With these results in hand, we proceeded toward the synthesis of *C*-glycosylated pyrrolizidine by testing the reductive N–O cleavage of **22a**. Molybdenum-mediated conditions (Mo­(CO)_6_ in MeCN–H_2_O^36,53^ ) promoted N–O bond opening but, contrary to expectations, no spontaneous lactamization was observed, even after prolonged reflux. Instead, ester **24** was isolated in a moderate yield (Scheme 2).

**Scheme 2:**
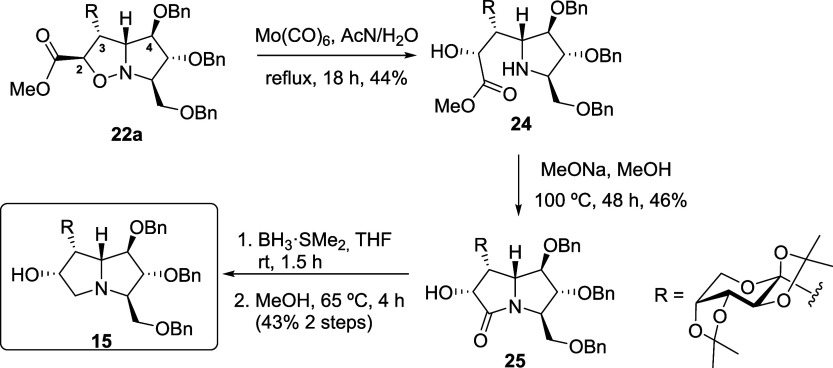
Synthetic Route toward Pyrrolizidine **15** from **22a**

Conversion of **24** into lactam **25** required the presence of sodium methoxide (Table 1).^54^ In all cases, long reaction times (>24 h) and high temperatures (>65 °C) were necessary to obtain lactam **25** in moderate yields (entries 2–4). Attempts to promote the transformation under microwave irradiation were completely ineffective (entries 5–7), leading to material loss and partial racemization at the α-position of the carbonyl group of **24**.^55^ Other reported reductive protocols, like catalytic hydrogenation over Raney-Ni^56^ or Pd^56^ or reduction by Zn in acetic acid,^17,18^ were unsuitable due to the presence of sensitive benzyl ethers and acetonide protecting groups in **24**. Finally, reduction of lactam **25** with borane-dimethyl sulfide complex afforded the target pyrrolizidine **15** (overall yield 3% from **21**), highlighting the reliability of this methodology for the synthesis of C-glycosylated pyrrolizidines (Scheme 2).

**Table 1:** Reaction Conditions Tested for the Lactamization of **24**

Entry	Reagent (equiv)	Solvent^a^	Conditions	Yield (% of 24)
**1**	NaOCH_3_ (1.6)	CH_3_OH	Reflux, 2 h	NR
**2**	NaOCH_3_ (1.6)	CH_3_OH	Sealed tube, 100 °C, 30 h	41
**3**	NaOCH_3_ (2.5)	CH_3_OH	Sealed tube, 100 °C, 48 h	46
**4**	NaOCH_3_ (3.0)	CH_3_OH	Sealed tube, 100 °C, 30 h	30
**5**	NaOCH_3_ (1.6)	CH_3_OH	Microwave, 65 °C, 30 min	NR
**6**	NEt_3_ (5.0)	Toluene	Microwave, 150 °C, 1 h	NR
**7**	AcONa (5.0)	CH_3_CN	Microwave, 130 °C, 1 h	NR

a All reactions were carried out using anhydrous solvents; NR: no reaction observed.

Similarly, cycloadduct **22b** was subjected to the same sequence of transformations as that applied to **22a**. In this case, the treatment of **22b** with Mo­(CO)_6_ at reflux for 18 h led to spontaneous cyclization, affording lactam **26**. Subsequent reduction with BH_3_·SMe_2_ furnished pyrrolizidine **16** in good yield (overall yield of 21% from **21**). On the other hand, when compound **26** was treated with NaOMe at 100 °C, epimerization occurred, leading to the formation of compound **27**. Subsequent reduction of **27** with BH_3_·SMe_2_ afforded pyrrolizidine **17** (overall yield of 17% from **21**) (Scheme 3).

**Scheme 3:**
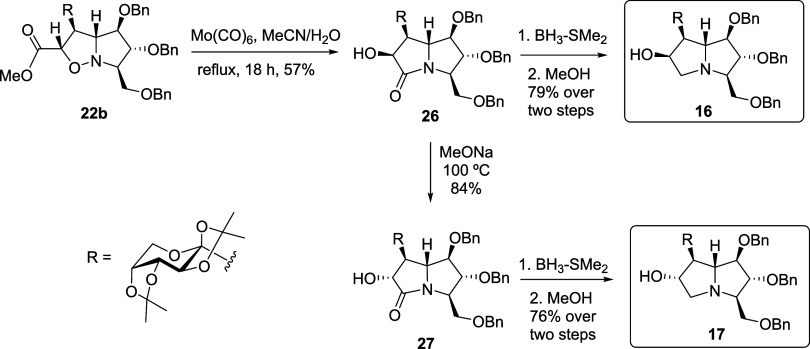
Synthetic Route toward Pyrrolizidines **16** and **17** from **22b**

The observed difference in lactamization rates between **22a** and **22b** can be attributed to their stereochemistry at C2 and C3. In **22a**, the *endo* orientation of the sugar group increases the steric hindrance around the reactive center, making cyclization more difficult. In contrast, in **22b**, the sugar adopts an *exo* orientation, which reduces steric hindrance and facilitates the reaction.

For the synthesis of *C*-glycosylated hyacinthacine, molybdenum-mediated reductive N–O cleavage of **23** was followed by amination and diastereoselective tautomerization in a tandem process, affording ketone **28** as a single stereoisomer.^57^ The newly formed stereocenter at C-5 oriented the methyl group toward the upper face of the bicyclic framework, stabilizing a low-energy conformation of **28**, in which most substituents are directed toward the less hindered convex face (Scheme 4). Ketone **28** was subsequently reduced to secondary alcohol **18** (overall yield 35% from **21**) using sodium borohydride in methanol at room temperature. Higher temperatures led to decreased diastereoselectivity, yielding mixtures of **18** and **19** (overall yield of 14% from **21**), whereas cooling the reaction to 0 °C resulted in no observable conversion (Scheme 4).

**Scheme 4:**
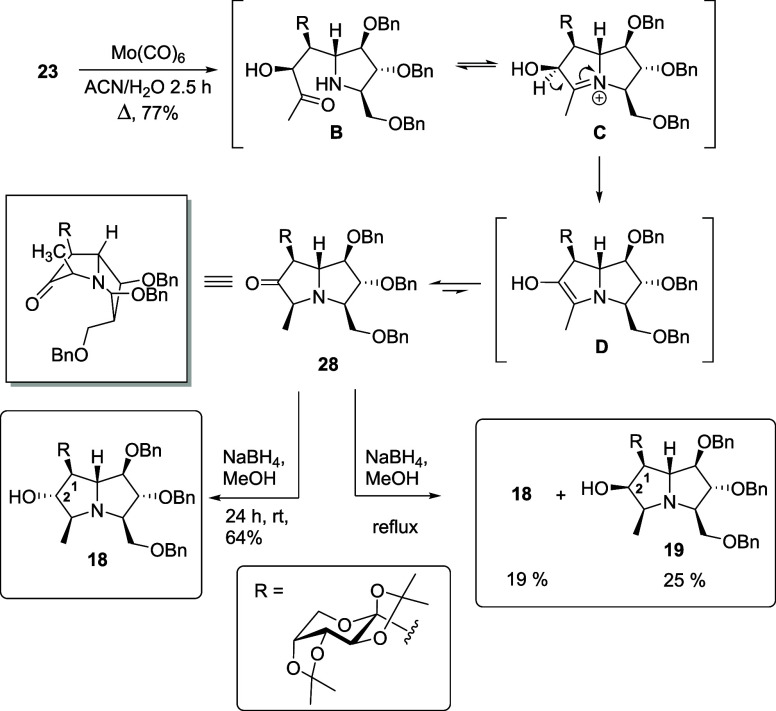
Transformation of Isoxazolidine **27** to *C*-Glycosylated Hyacinthacines **18** and **19**

The stereochemistry of ketone **28** and its reduction products, **18** and **19**, was determined by NOE experiments, as summarized in Figure 6. The orientation of the newly formed methyl group in **28** was established from its spatial interaction with the saccharide moiety on the upper face of the bicyclic system, as well as from the NOE correlation observed between H-3 and H-5 in derivative **18**. Furthermore, the stereochemistry at C-2 of the two alcohols obtained from the reduction of **28** was determined by diagnostic NOE interactions: between H-2 and the methyl group in **18**, and between H-2 and H-5 in **19**. These correlations collectively confirm the relative configurations of the reduction products.

**Figure 6:**
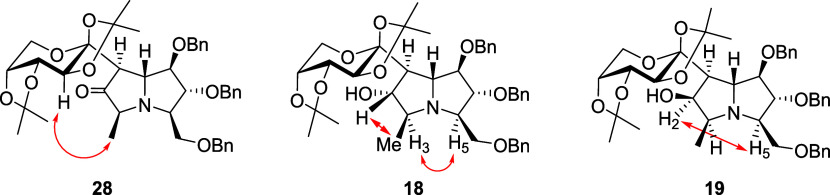
NOE correlations supporting the stereochemical assignments of ketone **28** and reduction products **18** and **19**.

As a proof of concept, the benzyl protecting groups of compound **18** were selectively removed while preserving the acetal moieties. This approach enabled the isolation of a well-defined intermediate, compound **29** (Scheme 5), without generating hemiacetal mixtures. Initial attempts under various conditions were unsuccessful; however, prolonged catalytic hydrogenation with Pd–C at 60 psi for 4 days afforded compound **29** in good yield (overall yield 29% from **21**). The fully characterized product confirmed the feasibility of selective benzyl deprotection in this system and provided a stable intermediate for further transformations.

**Scheme 5:**
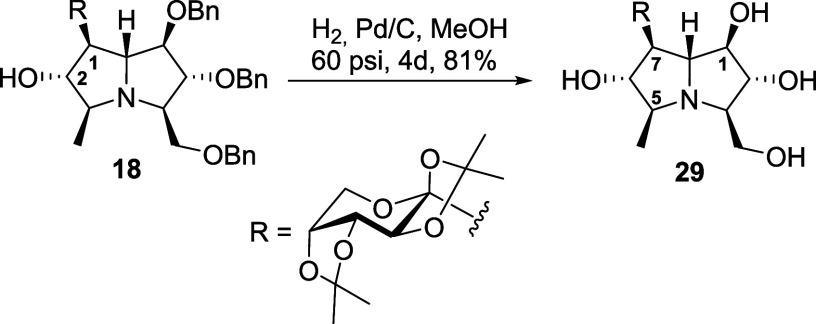
Selective Benzyl Deprotection of Pyrrolizidine **18** to Afford **29**

In summary, five protected C-glycosylated pyrrolizidines (**15**–**19**) have been successfully synthesized through a short and efficient sequence based on 1,3-dipolar cycloaddition of nitrone **21** with suitably functionalized alkenes. The methodology proved versatile and reliable, allowing access to both casuarine- and hyacinthacine-type derivatives. These results highlight the utility of this approach for the preparation of novel C-glycosylated iminosugars, providing a solid framework for the further exploration of their biological and structural properties.

## Conclusions

We have developed a concise and efficient synthetic route to five new C-glycosylated pyrrolizidines (**15–19**) using 1,3-dipolar cycloadditions of nitrone **21** with *E*-alkenes **20a** and **20b**. This strategy consistently delivered the desired isoxazolidine intermediates with high regio- and stereocontrol, highlighting the reliability of this methodology for complex iminosugar construction. Reductive transformations enabled access to both casuarine- and hyacinthacine-type frameworks, while stereochemical outcomes were established through NOE experiments.

The stereochemical outcome revealed a distinctive influence of the substituent on the dipolarophile: ester **20a** gave nearly equal amounts of *exo* and *endo* adducts, whereas ketone **20b** afforded exclusively the *endo* isomer of the carbonyl substituent, with the bulky sugar group adopting an *exo* orientation. This expands the understanding of stereocontrol in nitrone–alkene cycloadditions and emphasizes the critical role of the alkene geometry in directing product formation.

Overall, this work establishes an efficient platform for the preparation of novel iminosugar derivatives through 1,3-dipolar cycloadditions with *E*-alkenes, providing a solid foundation for further exploration of their structural stability and biological activity.

## Experimental Section

### Preparation of (2*R*,3*R*,3a*R*,4*R*,5*R*,6*R*)-Methyl 4,5-bis­(benzyloxy)-6-benzyloxymethyl-3-[(1*S*,2*S*,3*R*,4*R*)-(1,2:3,4-di-*O*-isopropylidene-β-D-arabinopyranos-1-yl)­hexahydropyrrolo­[1,2-*b*]­isoxazole-2-carboxylate, (22a)

A mixture of nitrone **21** (485 mg, 1.16 mmol) and alkene **20a** (584 mg, 1.86 mmol) in anhydrous CH_2_Cl_2_ (5 mL) was prepared in a screw cap tube. The reaction mixture was heated at 70 °C for 30 h and monitored by TLC, showing the disappearance of the nitrone and the appearance of two new products. The solvent was then evaporated under reduced pressure, and the residue was purified by flash chromatography (hexane-Et_2_O 5:1→2:1). Two products were isolated and identified as diastereoisomer **22a** (eluted first in hexane-Et_2_O 2:1, yellow oil, 319 mg, 38%) and **22b** (eluted second in hexane-Et_2_O 2:1, yellow oil, 383 mg, 45%). **22a**: *R*_*f*_ = 0.53 (hexane-Et_2_O 1:2); [α]_D_ ^26^ – 60.8 (*c* 0.3, CHCl_3_); ^1^H NMR (500 MHz, CDCl_3_) δ 7.38–7.26 (m, 15H, H_*Ar*_), 4.71 (bd, *J* = 6.1 Hz, 1H, H-2), 4.63–4.51 (m, 7H, H-3′, 3CH_2_Ph), 4.47 (brd, *J* = 2.7 Hz, 1H, H-2′), 4.34 (t, *J* = 5.5 Hz, 1H, H-4), 4.21 (dd, *J*_1_ = 8.0 Hz, *J*_2_ = 1.1 Hz, 1H, H-4′), 4.18 (t, *J* = 5.3 Hz, 1H, H-5), 3.93 (m, 2H, H-3, 3a), 3.85 (dd, *J*_1_ = 12.9 Hz, *J*_2_ = 1.8 Hz, 1H, H-5′_A_), 3.69 (d, *J* = 12.9 Hz, 1H, H-5′_B_), 3.64–3.56 (m, 3H, H-6,8_A_,8_B_), 3.59 (s, 3H, −OCH_3_), 1.55, 1.52, 1.48, and 1.35 (4s, 12H, 2CMe_2_); ^13^C NMR (125 MHz, CDCl_3_) δ 171.2 (C-7), 138.3, 138.2, 138.1 (C_*quatern Ar*_), 128.40, 128.36, 128.11, 127.79, 127.75, 127.73, 127.67, 127.64 (CH-Ar), 109.3 and 109.1 (2CMe_2_), 103.1 (C-1′), 86.2 (CH-5), 85.4 (CH-4), 79.5 (CH-2), 73.4, 72.38, 72.36 (3CH_2_Ph), 71.1 (CH-4′), 70.8, 70.5, 70.4 (CH-3a, 3′, 2′), 69.6 (CH_2_-8), 69.4 (CH-6), 61.5 (CH_2_-5′), 52.1 (−OCH_3_), 51.5 (CH-3), 26.6, 25.9, 25.1, and 24.5 (2CMe_2_); HRMS (TOF ESI^+^, *m*/*z*) 732.3358 [M + H]^+^, calcd for C_41_H_50_NO_11_ 732.3384.

### Preparation of (2*S*,3*S*,3a*R*,4*R*,5*R*,6*R*)-Methyl 4,5-bis­(benzyloxy)-6-benzyloxymethyl-3-[(1*S*,2*S*,3*R*,4*R*)-(1,2:3,4-di-*O*-isopropylidene-β-D-arabinopyranos-1-yl)­hexahydropyrrolo­[1,2-*b*]­isoxazole-2-carboxylate, (22b)

*R*_*f*_ = 0.37 (hexane-Et_2_O 1:2); ^1^H NMR (500 MHz, CDCl_3_) δ 7.37–7.28 (m, 15H, H_*Ar*_), 4.73 (d, *J* = 9.0 Hz, 1H, H-2), 4.67 and 4.54 (2d, *J* = 12.5 Hz, 2H, CH_2_Ph), 4.60 (m, 3H, H-3′, CH_2_Ph), 4.54 (m, 1H, CH_2_Ph), 4.44–4.42 (m, 2H, H-2′, CH_2_Ph), 4.32 (br s, 1H, H-4), 4.24 (dd, *J*_1_ = 9.7 Hz, *J*_2_ = 1.3 Hz, 1H, H-4′), 4.20 (br d, *J* = 10.5 Hz, 1H, H-3a), 4.01–3.95 (m, 2H, H-5, 5′_A_), 3.86 (m, 1H, H-6), 3.82–3.78 (m, 2H, CH_2_OBn_(A)_, 5′_B_), 3.73 (s, 3H, −OCH_3_), 3.60 (dd, *J*_1_ = 12.3 Hz, *J*_2_ = 9.3 Hz, 1H, CH_2_OBn_(B)_), 3.55 (dd, *J*_1_ = 10.5 Hz, *J*_2_ = 9.5 Hz, 1H, H-3), 1.56, 1.42, 1.38, and 1.33 (4s, 12H, 2CMe_2_); ^13^C NMR (125 MHz, CDCl_3_) δ 172.9 (COOMe), 138.6, 138.5, 138.2 (C_*quatern Ar*_), 128.3, 128.2, 127.9, 127.7, 127.6, 127.5 (CH-Ar), 109.2 and 108.5 (2CMe_2_), 103.3 (C-1′), 85.7 (CH-4), 84.9 (CH-5), 79.8 (CH-2), 73.3 (CH_2_Ph), 72.4 (CH-2′), 71.6 and 71.5 (2CH_2_Ph), 71.6 (CH-3a), 70.6, 70.3, 70.2 (CH-6, 4′, 3′), 70.4 (CH_2_OBn), 61.8 (CH_2_-5′), 58.1 (CH-3), 52.5 (−OCH_3_), 26.8, 25.8, 25.7, and 23.9 (2CMe_2_); HRMS (TOF ESI^+^, *m*/*z*) 732.3370 [M + H]^+^, calcd for C_41_H_50_NO_11_ 732.3384.

### Preparation of (2*R*,3*R*)-Methyl 3-[(2*R*,3*R*,4*R*,5*R*)-3,4-bis­(benzyloxy)-5-benzyloxymethyl-2-pyrrolidinyl]-2-hydroxy-3-[(1*S*,2*S*,3*R*,4*R*)-(1,2:3,4-di-*O*-isopropylidene-β-D-arabinopyranos-1-yl)­propanoate, (24)

Mo­(CO)_6_ (197 mg, 0.75 mmol) was added to a solution of **22a** (498 mg, 0.68 mmol) in MeCN–H_2_O (15:1, 20 mL). The mixture was refluxed under argon for 18 h, after which a new product with lower *R*_*f*_ was observed on TLC. The mixture was cooled and filtered through Celite washing with CH_2_Cl_2_. Evaporation of the reaction solvent afforded a residue that was purified by flash chromatography (hexane-Et_2_O 1:1→1:2) to yield the open product **24** as a yellow oil (217 mg, 44%): *R*_*f*_ = 0.27 (hexane-Et_2_O 1:2); [*a*]_D_ ^26^ + 1.6 (*c* 0.7, CHCl_3_); ^1^H NMR (500 MHz, CDCl_3_) δ 7.34–7.26 (m, 15H, H_*Ar*_), 4.62–4.60 (m, 3H, H-3′, CH_2_Ph), 4.53 (s, 2H, CH_2_Ph), 4.49 (brd, *J* = 2.7 Hz, 1H, H-2″), 4.47 (2d, *J* = 10.0 Hz, 2H, CH_2_Ph), 4.23–4.22 (m, 2H, H-2, 4′), 4.14 (dd, *J*_1_ = 7.8 Hz, *J*_2_ = 5.4 Hz, 1H, H-3″), 4.00 (t, *J* = 5.3 Hz, 1H, H-4″), 3.85 (dd, *J*_1_ = 12.8 Hz, *J*_2_ = 1.5 Hz, 1H, H-5′_A_), 3.78 (m, 1H, H-2″), 3.71 (d, *J* = 12.8 Hz, 1H, H-5′_B_), 3.63 (s, 3H, −OCH_3_), 3.54–3.46 (m, 3H, H-5″, CH_2_OBn), 3.31 (t, *J* = 6.3 Hz, 1H, H-3), 1.49, 1.42, 1.36, and 1.34 (4s, 12H, 2CMe_2_); ^13^C NMR (125 MHz, CDCl_3_) δ 173.3 (C-1), 138.7, 138.4, 138.2 (C_*quatern Ar*_), 128.50, 128.46, 128.39, 128.09, 128.02, 127.78, 127.77, 127.76, 127.66 (CH-Ar), 109.2 and 109.1 (2CMe_2_), 104.0 (C-1′), 88.0 (CH-3″), 85.2 (CH-4″), 74.0 (CH-2), 73.4, 72.5, and 72.0 (3CH_2_Ph), 71.1 (CH-4′), 70.8 (CH-2′), 70.6 (CH-3′), 69.0 (CH_2_OBn), 61.5 (CH_2_-5′), 60.7 (CH-5″), 59.5 (CH-2″), 51.7 (−OCH_3_), 44.6 (CH-3), 26.9, 25.8, 25.6, and 24.4 (2CMe_2_); HRMS (TOF ESI^+^, *m*/*z*) 734.3516 [M + H]^+^, calcd for C_41_H_52_NO_11_ 734.3535.

### Preparation of (1*R*,2*R*,5*R*,6*R,*7*R,*7a*R*)-6,7-Bis­(benzyloxy)-5-benzyloxymethyl-2-hydroxy-1-[(1*S*,2*S*,3*R*,4*R*)-(1,2:3,4-di-*O*-isopropylidene-β-D-arabinopyranos-1-yl)]­hexahydropyrrolizin-3-one, (25)

A solution of **24** (217 mg, 0.30 mmol) in anhydrous CH_3_OH (20 mL) was prepared in a screw cap tube. A 2 M methanolic solution of NaOCH_3_ (2.0 equiv, 0.30 mL) was then added, the tube was sealed, and the reaction mixture was stirred at 100 °C for 48 h. After this time, the solvent was evaporated under reduced pressure, and the residue was purified by flash chromatography (hexane-AcOEt 1:1 → EtOAc–MeCN–CH_3_OH–H_2_O 70:5:2.5:2.5) to give **25** (98 mg, 46%) as a colorless oil: *R*_*f*_ = 0.38 (EtOAc–CH_3_CN–CH_3_OH–H_2_O 70:5:2.5:2.5); [*a*]_D_ ^26^ – 20.4 (*c* 0.7, CHCl_3_); ^1^H NMR (600 MHz, CDCl_3_) δ 7.32–7.24 (m, 13H, H_*Ar*_), 7.21–7.19 (m, 2H, H_*Ar*_), 4.70 (d, *J* = 11.5 Hz, 1H, CH_2_Ph), 4.62–4.58 (m, 3H, H-2′, 3′, CH_2_Ph), 4.55–4.53 (m, 2H, H-7, CH_2_Ph), 4.51 (d, *J* = 11.8 Hz, 1H, CH_2_Ph), 4.46 (d, *J* = 12.0 Hz, 1H, CH_2_Ph), 4.40 (d, *J* = 11.8 Hz, 1H, CH_2_Ph), 4.36 (brs, 1H, H-2), 4.17 (brd, *J* = 7.9 Hz, 1H, H-4′), 4.08 (brd, *J* = 8.9 Hz, 1H, H-7a), 3.91 (t, *J* = 5.5 Hz, 1H, H-6), 3.86 (brs, 1H, H-5), 3.81 (d, *J* = 12.6 Hz, 1H, H-5′_A_), 3.63 (d, *J* = 12.8 Hz, 1H, H-5′_B_), 3.56 (dd, *J*_1_ = 10.3 Hz, *J*_2_ = 3.6 Hz, 1H, CH_2_OBn_(A)_), 3.45 (dd, *J*_1_ = 10.3 Hz, *J*_2_ = 5.7 Hz, 1H, CH_2_OBn_(B)_), 3.34 (brs, 1H, H-1), 1.49 (s, 3H, CMe_2_), 1.35 (s, 6H, CMe_2_), 1.31 (s, 3H, CMe_2_); ^13^C NMR (150 MHz, CDCl_3_) δ 176.5 (C-3), 137.9, 137.7, 137.1 (C_*quatern Ar*_), 128.69, 128.60, 128.59, 128.24, 128.12, 128.10, 128.03 (CH-Ar), 109.5 and 109.4 (2CMe_2_), 104.4 (C-1′), 85.6 (CH-7), 82.0 (CH-6), 73.5, 73.4, and 72.5 (3CH_2_Ph), 72.4 (CH-2), 71.1 (CH-4′), 70.7, 70.6 (CH-3′, CH-2′), 66.7 (CH_2_OBn), 61.6 (CH_2_-5′), 60.1 (CH-7a), 59.4 (CH-5), 44.9 (CH-1), 26.8, 26.2, 25.6, and 24.5 (2CMe_2_); HRMS (TOF ESI^+^, *m*/*z*) 702.3263 [M + H]^+^, calcd for C_40_H_48_NO_10_ 702.3278.

### Preparation of (1*R*,2*R*,5*R*,6*R,*7*R,*7a*R*)-6,7-Bis­(benzyloxy)-5-benzyloxymethyl-1-[(1*S*,2*S*,3*R*,4*R*)-(1,2:3,4-di-*O*-isopropylidene-β-D-arabinopyranos-1-yl)]­hexahydro-1*H*-pyrrolizin-2-ol, (15)

To a solution of **25** (98 mg, 0.14 mmol) in anhydrous THF (8 mL), BH_3_·S­(CH_3_)_2_ (106 mg, 1.40 mmol) was added dropwise. The mixture was stirred under argon at rt for 1.5 h. After this time, a new product with higher *R*_*f*_ could be observed on TLC. The reaction was then quenched by careful addition of CH_3_OH (1 mL) at rt and subsequent evaporation of the organic solvent under vacuum. The remaining residue was resuspended in CH_3_OH (5 mL) and stirred at reflux for 4 h. TLC showed the disappearance of the high *R*_*f*_ N–B complexes, and the mixture was cooled down to rt and evaporated under reduced pressure. The residue was purified by flash chromatography (CH_2_Cl_2_–CH_3_OH 10:0.5) to yield **15** (43 mg, 43%) as a colorless oil: *R*_*f*_ = 0.30 (CH_2_Cl_2_–CH_3_OH 10:0.5); [*a*]_D_ ^26^ – 41.6 (*c* 0.9, CHCl_3_); ^1^H NMR (600 MHz, CDCl_3_) δ 7.35–7.26 (m, 15H, H_*Ar*_), 4.61 (dd, *J*_1_ = 7.8 Hz, *J*_2_ = 2.4 Hz, 1H, H-3′), 4.60–4.55 (m, 5H, H-2′, 2CH_2_Ph), 4.50 and 4.48 (2d, *J* = 12.0 Hz, 2H, CH_2_Ph), 4.22 (brd, *J* = 7.9 Hz, 1H, H-4′), 4.20 (dd, *J*_1_ = 7.1 Hz, *J*_2_ = 4.5 Hz, 1H, H-7a), 4.06 (d, *J* = 2.9 Hz, 1H, H-6), 4.03 (dd, *J*_1_ = 7.5 Hz, *J*_2_ = 5.3 Hz, 1H, H-7), 4.00 (dd, *J*_1_ = 12.0 Hz, *J*_2_ = 4.5 Hz, 1H, CH_2_OBn_(A)_), 3.90–3.87 (m, 2H, H-1, 5′_A_), 3.82 (dd, *J*_1_ = 11.7 Hz, *J*_2_ = 3.6 Hz, 1H, CH_2_OBn_(B)_), 3.70 (d, *J* = 12.9 Hz, 1H, H-5′_B_), 3.49 (dd, *J*_1_ = 9.6 Hz, *J*_2_ = 6.2 Hz, 1H, H-3_A_), 3.45 (dd, *J*_1_ = 9.7 Hz, *J*_2_ = 5.1 Hz, 1H, H-3_B_), 3.33 (q, *J* = 3.6 Hz, 1H, H-2), 2.32 (m, 1H, H-5), 1.45, 1.45, 1.39, and 1.33 (4s, 12H, 2CMe_2_); ^13^C NMR δ (150 MHz, CDCl_3_) 138.3, 138.2, 138.0 (C_*quatern Ar*_), 128.61, 128.58, 128.56, 128.10, 128.08, 127.97, 127.91, 127.86 (C*H*-Ar), 109.1 and 109.0 (2CMe_2_), 104.5 (C-1′), 86.2 (CH-7a), 85.1 (CH-1), 75.5 (CH-6), 73.4 (CH_2_Ph), 72.2 (2CH_2_Ph), 71.2 (CH-4′), 70.64, 70.61 (CH-3′, CH-2′), 69.1 (CH_2_-3), 64.7 (CH_2_-8), 61.5 (CH-7), 61.4 (CH_2_-5′), 61.1 (CH-5), 39.0 (CH-2), 27.0, 26.1, 25.9, and 24.3 (2CMe_2_); HRMS (TOF ESI^+^, *m*/*z*) 706.3601 [M + H + H_2_O]^+^, calcd for C_40_H_52_NO_10_ 706.3586.

### Preparation of (1*S*,2*S*,5*R*,6*R,*7*R,*7a*R*)-6,7-Bis­(benzyloxy)-5-benzyloxymethyl-2-hydroxy-1-[(1*S*,2*S*,3*R*,4*R*)-(1,2:3,4-di-*O*-isopropylidene-β-D-arabinopyranos-1-yl)]­hexahydropyrrolizin-3-one, (26)

Mo­(CO)_6_ (159 mg, 0.60 mmol) was added to a solution of **22b** (400 mg, 0.55 mmol) in MeCN–H_2_O (15:1, 16 mL). The mixture was refluxed under argon for 18 h, after which a new product with lower *R*_*f*_ was observed on TLC. The mixture was cooled and filtered through Celite washing with CH_2_Cl_2_. Evaporation of the reaction solvent afforded a residue that was purified by flash chromatography (hexane-Et_2_O 1:1→1:2) to yield the product **26** as a yellow oil (230 mg, 57%): *R*_*f*_ = 0.4 (AcOEt-hexane 2:1); [*a*]_D_ ^28^ – 38 (*c* 1.0, CHCl_3_); ^1^H NMR (500 MHz, CDCl_3_) δ 7.28–7.19 (m, 15H, H_*Ar*_), 4.59–4.45 (m, 8H, H-2′, H-3′, H-5, 5xCH_2_Ph), 4.33–4.30 (m, 3H, H-2, H-7a, CH_2_Ph), 4.20 (bd, *J* = 8.0 Hz, 1H, H-4′), 4.12 (s, 1H, H-6), 4.06 (bs, 1H, H-7), 3.97 (bd, *J* = 13.02 Hz, 1H, H-5′a), 3.72 (d, 1H, H-5′b), 3.59–3.56 (m, 2H, CH_2_OBn), 2.62 (dd, *J* = 8.9, 5.2 Hz, H-1), 1.52, 1.39, 1.30, and 1.28 (4s, 12H, 2CMe_2_); ^13^C NMR (125 MHz, CDCl_3_) δ 175.1 (C-3), 138.06, 137.99, 137.4 (C_*quatern Ar*_), 128.3, 128.0, 127.66, 127.60, 127.54, 127.51 (CH-Ar), 109.2 and 109.1 (2CMe_2_), 103.0 (C-1′), 87.2 (CH-7), 84.2 (C-6), 74.0 and 66.5 (CH-2, 7a), 73.0, 72.0, and 71.1 (3CH_2_Ph), 71.7 and 58.8 (CH-5,2′), 70.3 (CH-4′), 69.9 (C-3′), 68.1 (CH_2_–OBn), 61.5 (CH_2_-5′), 51.9 (CH-1), 26.8, 25.9, 25.5, and 23.5 (2CMe_2_); HRMS (TOF ESI^+^, *m*/*z*) 702.3248 [M + H]^+^, calcd for C_40_H_48_NO_10_ 702.3278.

### Preparation of (1*S*,2*S*,5*R*,6*R,*7*R,*7a*R*)-6,7-Bis­(benzyloxy)-5-benzyloxymethyl-1-[(1*S*,2*S*,3*R*,4*R*)-(1,2:3,4-di-*O*-isopropylidene-β-D-arabinopyranos-1-yl)]­hexahydro-1*H*-pyrrolizin-2-ol, (16)

To a solution of **26** (40 mg, 0.057 mmol) in anhydrous THF (6 mL), BH_3_·S­(CH_3_)_2_ (2 M, 0.28 mL, 0.57 mmol) was added dropwise. The mixture was stirred under argon at rt for 1.5 h. After this time, a new product with higher *R*_*f*_ could be observed on TLC. The reaction was then quenched by careful addition of CH_3_OH (1 mL) at rt and subsequent evaporation of the organic solvent under vacuum. The remaining residue was resuspended in CH_3_OH (5 mL) and stirred at reflux for 4 h. TLC showed the disappearance of the high *R*_*f*_ N–B complexes, and the mixture was cooled down to rt and evaporated under reduced pressure. The residue was purified by flash chromatography (AcOEt → AcOEt-CH_3_OH 10:0.5) to yield **16** (31 mg, 79%) as a colorless oil: *R*_*f*_ = 0.40 (AcOEt); [*a*]_D_ ^29^ – 34.8 (*c* 1.0, CHCl_3_); ^1^H NMR (500 MHz, CDCl_3_) δ 7.32–7.25 (m, 15H, H_*Ar*_), 4.62–4.47 (m, 8H, H-2,2′,3′,5xCH_2_Ph), 4.43 (d, *J* = 11.93 Hz, 1H, CH_2_Ph), 4.27 (s, 1H, H-7), 4.23 (d, *J*_3′,4′_ = 7.9 Hz, *J*_4′,5′_ = 1.9 Hz, 1H, H-4′), 4.15 (s, 1H, H-6), 4.05 (bs, 1H, H-7a), 4.00 (dd, *J*_5′a,5′b_ = 13.0 Hz, *J*_4′,5′a_ = 2.0 Hz, 1H, H-5′a), 3.75 (d, 1H, H-1, 5′b), 3.55–3.62 (m, 1H, CH_2_OBn), 3.32 (m, 2H, H-3a,5), 3.09 (dd, *J*_3a,3b_ = 10.1 Hz, *J*_2,3b_ = 3.1 Hz, 1H, H-3b), 2.63 (dd, *J*_1_ = 10.5 Hz, *J*_2_ = 3.8 Hz, 1H, H-1), 1.45, 1.38, 1.37, and 1.31 (4s, 12H, 2CMe_2_); ^13^C NMR δ (125 MHz, CDCl_3_) 138.7, 138.6, 138.2 (C_*quatern Ar*_), 128.27, 128.21, 128.1, 127.64, 127.58, 127.48, 127.36, 127.34, 127.22 (CH-Ar), 109.3 and 108.5 (2CMe_2_), 104.4 (C-1′), 87.9 (CH-7), 86.0 (CH-6), 73.4 (CH-2), 73.1 (CH_2_Ph), 72.1 (CH-2′), 71.9 (CH_2_–OBn), 71.4 and 71.2 (2CH_2_Ph), 70.5 (CH-4′), 70.3 (CH-3′), 70.0 (CH-5), 69.3 (CH-7a), 63.8 (CH-3), 61.4 (CH_2_-5′), 53.9 (CH-1), 26.7, 25.8, 25.7, and 23.8 (2CMe_2_); HRMS (TOF ESI^+^, *m*/*z*) 688.3445 [M + H]^+^, calcd for C_40_H_50_NO_9_ 688.3486.

### Preparation of (1*S*,2*R*,5*R*,6*R,*7*R,*7a*R*)-6,7-Bis­(benzyloxy)-5-benzyloxymethyl-2-hydroxy-1-[(1*S*,2*S*,3*R*,4*R*)-(1,2:3,4-di-*O*-isopropylidene-β-D-arabinopyranos-1-yl)]­hexahydropyrrolizin-3-one, (27)

A solution of **26** (75 mg, 0.32 mmol) in anhydrous CH_3_OH (10 mL) was prepared in a screw cap tube. A 2 M methanolic solution of NaOCH_3_ (2.0 equiv, 0.32 mL) was then added, the tube was sealed, and the reaction mixture was stirred at 60 °C for 24 h. After this time, the solvent was evaporated under reduced pressure, and the residue was purified by flash chromatography (hexane-Et_2_O 1:1→1:2) to give **27** (63 mg, 84%) as a colorless oil: *R*_*f*_ = 0.5 (AcOEt-hexane 2:1); [*a*]_D_ ^27^ – 21.8 (*c* 0.1, CHCl_3_); ^1^H NMR (400 MHz, CDCl_3_) δ 7.32–7.14 (m, 15H, H_*Ar*_), 4.65–4.62 (m, 2H, H-2′, CH_2_Ph), 4.60 (dd, 1H, *J* = 7.9 Hz, *J* = 2.8 Hz, 1H, H-3′), 4.56 (d, 1H, *J* = 10.1 Hz, H-2), 4.51 (m, 1H, H-5), 4.50 (s, 2H, CH_2_Ph), 4.45–4.42 (m, 2H, CH_2_Ph), 4.28 (d, *J* = 11.9 Hz, 1H, CH_2_Ph), 4.20 (brd, 1H, *J* = 7.9 Hz, H-4′), 4.09 (brs, 1H, H-7), 4.00–3.93 (m, 3H, H-6, 7a, 5′_a_), 3.68 (d, *J* = 12.8 Hz, 1H, H-5′_b_), 3.54–3.51 (m, 2H, CH_2_OBn), 2.72 (t, 1H, *J* = 9.9 Hz, H-1), 1.56 (s, 3H, CMe_2_), 1.45 (s, 3H, CMe_2_), 1.28 (s, 6H, CMe_2_); ^13^C NMR (100 MHz, CDCl_3_) δ 176.5 (C-3), 138.2, 138.0, 137.5 (C_*quatern Ar*_), 128.30, 128.27, 128.24, 127.67, 127.62, 127.61, 127.55, 127.55 (CH-Ar), 109.0 and 108.8 (2CMe_2_), 102.9 (C-1′), 87.4 (CH-7), 83.8 (CH-6), 73.0, 72.3, and 70.9 (3CH_2_Ph), 71.3 and 71.2 (CH-2,2′), 70.7 (CH-4′), 70.0 (CH-3′), 67.8 (CH_2_OBn), 63.4 (CH-7a), 61.4 (CH_2_-5′), 59.5 (CH-5), 56.8 (CH-1), 26.8, 25.8, 25.4, and 23.7 (2CMe_2_); HRMS (TOF ESI^+^, *m*/*z*) 702.3278 [M + H]^+^, calcd for C_40_H_48_NO_10_ 702.3251.

### Preparation of (1*S*,2*R*,5*R*,6*R,*7*R,*7a*R*)-6,7-Bis­(benzyloxy)-5-benzyloxymethyl-1-[(1*S*,2*S*,3*R*,4*R*)-(1,2:3,4-di-*O*-isopropylidene-β-D-arabinopyranos-1-yl)]­hexahydro-1*H*-pyrrolizin-2-ol, (17)

To a solution of **27** (43 mg, 0.061 mmol) in anhydrous THF (6 mL), BH_3_·S­(CH_3_)_2_ (2 M, 0.3 mL, 0.6 mmol) was added dropwise. The mixture was stirred under argon at rt for 1.5 h. After this time, a new product with higher *R*_*f*_ could be observed on TLC. The reaction was then quenched by careful addition of CH_3_OH (1 mL) at rt and subsequent evaporation of the organic solvent under vacuum. The remaining residue was resuspended in CH_3_OH (5 mL) and stirred at reflux for 4 h. TLC showed the disappearance of the high *R*_*f*_ N–B complexes, and the mixture was cooled down to rt and evaporated under reduced pressure. The residue was purified by flash chromatography (AcOEt → AcOEt-CH_3_OH 10:0.5) to yield **17** (32 mg, 76%) as a colorless oil: *R*_*f*_ = 0.40 (AcOEt); [*a*]_D_ ^27^ – 22.2 (*c* 1.0, CHCl_3_); ^1^H NMR (600 MHz, CD_3_CN) δ 7.37–7.28 (m, 15H, H_*Ar*_), 4.65–4.47 (m, 8H, H-2′,3′,6xCH_2_Ph), 4.36 (m, 1H, H-2), 4.26 (s, 1H, H-7a), 4.22 (dd, *J*_3′,4′_ = 8.0 Hz, *J*_4′,5′_ = 1.8 Hz, 1H, H-4′), 4.02 (s, 1H, H-7), 3.93 (dd, *J*_5′a,5′b_ = 13.9 Hz, *J*_4′,5′a_ = 1.9 Hz, 1H, H-5′a), 3.80 (bs, 1H, H-6), 3.65 (d, 1H, H-5′b), 3.58–3.51 (m, 3H, H-5, CH_2_OBn), 3.36 (m, 1H, H-3a), 3.05 (m, 1H, H-3b), 2.60 (m, 1H, H-1), 1.53, 1.42, 1.35, and 1.30 (4s, 12H, 2CMe_2_); ^13^C NMR δ (150 MHz, CD_3_CN) 138.7, 138.21, 138.19 (C_*quatern Ar*_), 128.28, 128.27, 127.80, 127.71, 127.65, 127.56, 127.44 (CH-Ar), 110.0 and 108.5 (2CMe_2_), 104.3 (C-1′), 88.4 (CH-7a), 86.1 (CH-7), 72.6 (CH_2_Ph), 72.05 (CH-2), 71.5 (CH_2_Ph), 71.4 (CH-2′), 71.3 (CH_2_Ph), 70.8 (CH-6), 70.7 (CH_2_–OBn), 70.4 (CH-4′), 70.1 (CH-3′), 69.7 (CH-5), 61.6 (CH-3), 61.20 (CH_2_-5′), 56.6 (CH-1), 26.0, 25.5, 25.1, and 23.1 (2CMe_2_); HRMS (TOF ESI^+^, *m*/*z*) 688.3519 [M + H]^+^, calcd for C_40_H_50_NO_9_ 688.3486

### Preparation of (2*S*,3*S*,3a*R*,4*R*,5*R*,6*R*)-2-Acetyl-4,5-bis­(benzyloxy)-6-benzyloxymethyl-3-[(1*S*,2*S*,3*R*,4*R*)-(1,2:3,4-di-*O*-isopropylidene-β-D-arabinopyranos-1-yl)]­hexahydropyrrolo­[1,2-*b*]­isoxazole, (23)

A mixture containing nitrone **21** (680 mg, 1.62 mmol) and alkene **20b** (775 mg, 2.60 mmol) in anhydrous CH_2_Cl_2_ (5 mL) was prepared in a screw cap tube. The reaction mixture was heated at 70 °C for 18 h and monitored by TLC, showing the formation of a new product. The solvent was removed under reduced pressure to yield a viscous residue, which was purified by flash chromatography (hexane-Et_2_O 2:1→hexane-Et_2_O 1:1) to give **23** (565 mg, 71% yield) as a pure yellow oil: *R*_*f*_ = 0.29 (hexane-AcOEt 2:1); [α]_D_ ^26^ – 41.5 (*c* 1.3, CHCl_3_); ^1^H NMR (500 MHz, CDCl_3_) δ 7.34–7.23 (m, 15H, H_*Ar*_), 4.62 and 4.52 (2d, *J* = 11.9 Hz, 2H, CH_2_Ph), 4.59 (d, *J* = 7.3 Hz, 1H, H-2), 4.57 (dd, *J*_1_ = 5.2 Hz, *J*_2_ = 2.6 Hz, 1H, H-3′), 4.55 (brs, 2H, CH_2_Ph), 4.47 and 4.38 (2d, *J* = 11.8 Hz, 2H, CH_2_Ph), 4.33 (brd, *J* = 2.6 Hz, 1H, H-2′), 4.21 (m, 1H, H-4), 4.20 (dd, *J*_1_ = 5.2 Hz, *J*_2_ = 1.4 Hz, 1H, H-4′), 4.16 (dd, *J*_1_ = 7.6 Hz, *J*_2_ = 1.8 Hz, 1H, H-3a), 3.95–3.91 (m, 2H, H-5, 5′_A_), 3.76 (d, *J* = 13.0 Hz, H-5′_B_), 3.70 (dd, *J*_1_ = 8.8 Hz, *J*_2_ = 4.6 Hz, 1H, CH_2_OBn_(A)_), 3.53–3.46 (m, 2H, H-6, CH_2_OBn_(B)_), 3.39 (t, *J* = 7.5 Hz, 1H, H-3), 2.25 (s, 3H, CH_3_), 1.50, 1.39, 1.32, and 1.31 (4s, 12H, 2CMe_2_); ^13^C NMR (125 MHz, CDCl_3_) δ 208.2 (CO), 138.5, 138.4, 138.1 (C_*quatern Ar*_), 128.47, 128.46, 128.41, 128.10, 127.92, 127.90, 127.76, 127.70, 127.68 (CH-Ar), 109.3 and 108.6 (2CMe_2_), 103.6 (C-1′), 86.9 (CH-4), 85.5 (CH-5), 85.2 (CH-2), 73.5 (CH_2_Ph), 72.29, 72.25 (CH-2′, 3a), 71.84 and 71.79 (2CH_2_Ph), 71.1 (CH-6), 70.7 (CH-4′), 70.7 (CH_2_OBn), 70.5 (CH-3′), 61.8 (CH_2_-5′), 56.4 (CH-3), 27.3 (CH_3_), 26.8, 26.0, 25.7, 24.0 (2CMe_2_); HRMS (LSIMS^+^, *m*/*z*) 738.3247 [M + Na]^+^, calcd for C_41_H_49_NO_10_Na 738.3254; LRMS (ESI^+^, *m*/*z*) 202, 242, 440, 738.

### Preparation of (1*S*,3*S*,5*R*,6*R,*7*R,*7a*R*)-6,7-Bis­(benzyloxy)-5-benzyloxymethyl-1-[(1*S*,2*S*,3*R*,4*R*)-(1,2:3,4-di-*O*-isopropylidene-β-D-arabinopyranos-1-yl)]-3-methylhexahydropyrrolizin-2-one, (28)

To a solution of **23** (309 mg, 0.43 mmol) in CH_3_CN–H_2_O (15:1,35 mL), Mo­(CO)_6_ (126 mg, 0.47 mmol) was added. The solution was refluxed under argon for 2.5 h, after which a new product with higher *R*_*f*_ could be observed on TLC. The mixture was cooled and filtered through Celite washing with CH_2_Cl_2_. Evaporation of the reaction solvent afforded a residue that was purified by flash chromatography (hexane-AcOEt 4:1) to give **28** (239 mg, 77%) as a colorless oil: *R*_*f*_ = 0.40 (hexane-AcOEt 3:1); [α]_D_ ^26^ – 7.3 (*c* 0.9, CHCl_3_); ^1^H NMR (500 MHz, CDCl_3_) δ 7.37–7.13 (m, 15H, H_*Ar*_), 5.13 (d, *J* = 2.8 Hz, 1H, H-2′), 4.69 and 4.54 (2 d, *J* = 12.1 Hz, 2H, CH_2_Ph), 4.63 (dd, *J*_1_ = 7.9 Hz, *J*_2_ = 2.9 Hz, 1H, H-3′), 4.52–4.47 (m, 2H, CH_2_Ph), 4.38–4.32 (m, 3H, H-7a, CH_2_Ph), 4.30 (brs, 1H, H-7), 4.20 (brd, *J* = 7.9 Hz, 1H, H-4′), 3.96 (d, *J* = 12.9 Hz, 1H, H-5′_A_), 3.86 (brd, *J* = 4.5 Hz, 1H, H-6), 3.68 (d, *J* = 12.9 Hz, 1H, H-5′_B_), 3.54 (m, 2H, CH_2_OBn), 3.48 (q, *J* = 7.3 Hz, 1H, H-3), 2.98 (m, 1H, H-5), 2.90 (brd, *J* = 10.6 Hz, 1H, H-1), 1.51, 1.29, 1.27, and 1.23 (4s, 12H, 2CMe_2_), 1.21 (d, *J* = 7.1 Hz, 3H, CH_3_); ^13^C NMR (125 MHz, CDCl_3_) δ 217.7 (CO), 138.8, 138.5, 138.1 (C_*quatern Ar*_), 128.50, 128.41, 128.37, 128.16, 127.81, 127.80, 127.69, 127.64, 127.61 (CH-Ar), 109.22 and 109.16 (2CMe_2_), 102.8 (C-1′), 87.6 (CH-7), 87.1 (CH-6), 73.4 (CH_2_Ph), 73.2 (CH_2_OBn), 71.6 and 71.4 (2CH_2_Ph), 70.8 (CH-4′), 70.4 (CH-5), 70.2 (CH-3′), 70.0 (CH-2′), 68.1 (CH-3), 67.4 (CH-7a), 61.8 (CH_2_-5′), 53.1 (CH-1), 27.3, 24.0, 26.0, 25.6 (2CMe_2_), 16.8 (CH_3_); HRMS (LSIMS^+^, *m*/*z*) 700.3469 [M + H]^+^, calcd for C_41_H_50_NO_9_ 700.3486. LRMS (ESI^+^, *m*/*z*): 244, 271, 382, 700, 722.

### Preparation of (1*R*,2*R*,3*S*,5*R*,6*R,*7*R,*7a*R*)-6,7-Bis­(benzyloxy)-5-benzyloxymethyl-1-[(1*S*,2*S*,3*R*,4*R*)-(1,2:3,4-di-*O*-isopropylidene-β-D-arabinopyranos-1-yl)]-3-methylhexahydro-1*H*-pyrrolizin-2-ol, (18)

A solution of ketone **28** (239 mg, 0.34 mmol, 1.0 equiv) in CH_3_OH (5 mL) was cooled to 0 °C, and NaBH_4_ (25 mg, 0.68 mmol, 2.0 equiv) was added portionwise. The mixture was then gradually warmed to rt and stirred for 24 h (small portions of NaBH_4_ were added in 4 h intervals until the starting material was consumed, according to TLC analysis). The reaction was quenched by cooling to 0 °C and addition of sat. NH_4_Cl solution. The organic solvent was then removed by evaporation under reduced pressure, and the crude was extracted from the remaining aqueous layer with 50 mL of CH_2_Cl_2_ (×3). The combined organic extracts were dried on Na_2_SO_4_, filtered, and evaporated under reduced pressure. The final residue was purified by flash chromatography (hexane-AcOEt 3:1) to afford **18** (154 mg, 64%) as a colorless oil: *R*_*f*_ = 0.50 (hexane-AcOEt 1:3); [α]_D_ ^26^ – 18.1 (*c* 1.7, CHCl_3_); ^1^H NMR (500 MHz, CDCl_3_) δ 7.34–7.20 (m, 15H, H_*Ar*_), 4.60 (dd, *J*_1_ = 7.9 Hz, *J*_2_ = 2.5 Hz, 1H, H-3′), 4.57–4.53 (m, 2H, H-2′, CH_2_Ph), 4.52–4.45 (m, 4H, 2CH_2_Ph), 4.40 (d, *J* = 11.9 Hz, 1H, CH_2_Ph), 4.29 (brs, 1H, H-7), 4.22 (brd, *J* = 7.8 Hz, 1H, H-4′), 4.19 (brs, 1H, H-6), 4.14 (brs, 1H, CH-2), 4.07 (brd, *J* = 10.6 Hz, 1H, H-7a), 4.01 (brd, *J* = 12.5 Hz, 1H, H-5′_A_), 3.90 (brs, 1H, OH), 3.72 (d, *J* = 13.0 Hz, 1H, H-5′_B_), 3.57 (m, 1H, CH_2_OBn_(A)_) 3.51 (m, 1H, CH_2_OBn_(B)_), 3.38 (brdd, *J*_1_ = 10.0 Hz, *J*_2_ = 6.0 Hz, 1H, H-5), 3.24 (m, 1H, H-3), 2.73 (dd, *J*_1_ = 10.6 Hz, *J*_2_ = 2.7 Hz, 1H, H-1), 1.53, 1.40, 1.33, and 1.26 (4s, 12H, 2CMe_2_), 1.16 (d, *J* = 6.2 Hz, 3H, CH_3_); ^13^C NMR (125 MHz, CDCl_3_) δ 139.0, 138.9, 138.4 (C_*quatern Ar*_), 128.44, 128.38, 128.30, 127.81, 127.71, 127.60, 127.47, 127.32 (CH-Ar), 109.4 and 108.8 (2CMe_2_), 104.2 (C-1′), 88.9 (CH-7), 86.4 (CH-6), 75.2 (CH-2), 73.3 (CH_2_Ph), 72.4 (CH_2_OBn), 72.1 (CH-2′), 71.5, 71.2 (2CH_2_Ph), 70.8 (CH-4′), 70.4, 70.3 (CH-3′, 7a), 68.0 (CH-5), 66.8 (CH-3), 61.6 (CH_2_-5′), 54.1 (CH-1), 27.3, 26.3, 25.8, and 23.8 (2CMe_2_), 15.4 (CH_3_); HRMS (LSIMS^+^, *m*/*z*) 702.3638 [M + H]^+^, calcd for C_41_H_52_NO_9_ 702.3642.

### Preparation of (1*R*,2*S*,3*S*,5*R*,6*R,*7*R,*7a*R*)-6,7-Bis­(benzyloxy)-5-benzyloxymethyl-1-[(1*S*,2*S*,3*R*,4*R*)-(1,2:3,4-di-*O*-isopropylidene-β-D-arabinopyranos-1-yl)]-3-methylhexahydro-1*H*-pyrrolizin-2-ol, (19)

A solution of ketone **28** (86 mg, 0.12 mmol) in CH_3_OH (4 mL) was cooled to 0 °C, and NaBH_4_ (9 mg, 0.24 mmol) was added portionwise. The mixture was then refluxed under argon for 18 h. After this time, two new products were seen on TLC. The mixture was then cooled to rt, and the solvent was removed under vacuum. The reaction crude was purified by flash chromatography (hexane-AcOEt 5:1→1:1) to yield **18** (16 mg, 19%) and **19** (21 mg, 25%, colorless liquid). **19**: *R*_*f*_ = 0.40 (hexane-AcOEt 1:3); ^1^H NMR (600 MHz, CDCl_3_) δ 7.40–7.19 (m, 15H, H_*Ar*_), 4.60–4.52 (m, 4H, H-3′, CH_2_Ph, CH_2_Ph), 4.50 (brd, *J* = 11.9 Hz, 1H, CH_2_Ph), 4.44 (d, *J* = 12.5 Hz, 1H, CH_2_Ph), 4.41 (brd, *J* = 11.9 Hz, 1H, CH_2_Ph), 4.34 (brs, 1H, H-2′), 4.18 (brd, *J* = 7.7 Hz, 1H, H-4′), 4.04 (brs, 2H, H-2, 7_a_), 3.99 (brs, 2H, H-6, 7), 3.89 (brd, *J* = 12.9 Hz, 1H, H-5′_A_), 3.76 (brd, *J* = 13.0 Hz, 1H, H-5′_B_), 3.52 (brs, 1H, OH), 3.49 (brs, 1H, CH_2_OBn_(A)_), 3.40 (brs, 1H, CH_2_OBn_(B)_), 3.31 (brs, 1H, H-5), 3.21 (brs, 1H, H-3), 2.70 (t, *J* = 8.1 Hz, 1H, H-1), 1.50, 1.40, 1.30, and 1.28 (4s, 12H, 2CMe_2_), 1.23 (d, *J* = 6.9 Hz, 3H, CH_3_); ^13^C NMR (125 MHz, CDCl_3_) δ 128.52, 128.40, 127.90, 127.80, 127.71, 127.55 (CH-Ar), 109.4 (2CMe_2_), 105.9 (C-1′), 87.6, 86.5 (CH-6, CH-7), 78.8 (CH-2), 73.7, 73.4 (CH-2′, CH_2_OBn, CH_2_Ph), 71.7 (CH_2_Ph), 71.4 (CH_2_Ph), 70.7, 70.4 (CH-3′, CH-4′), 69.8 (CH-7a), 64.7 (CH-3), 62.8 (CH-5), 61.6 (CH_2_-5′), 51.7 (CH-1), 26.7, 26.2, 25.5, and 24.1 (2CMe_2_), 15.6 (CH_3_).

### Preparation of (1*R*,2*R*,3*R*,5*S*,6*R,*7*R,*7a*R*)-3-Hydroxymethyl-7-[(1*S*,2*S*,3*R*,4*R*)-(1,2:3,4-di-*O*-isopropylidene-β-D-arabinopyranos-1-yl)]-5-methylhexahydro-1*H*-pyrrolizin-1,2,6-triol, (29)

A solution of **18** (30 mg, 0.04 mmol) in CH_3_OH (2 mL) was hydrogenated at room temperature (60 psi of H_2_) in the presence of 10% Pd–C (25 mg) for 4 days. The reaction was monitored by LCMS. The catalyst was filtered off, washed with CH_3_OH, and the filtrate and washings were evaporated to a residue that was purified using RP C-18 chromatography (H_2_O-MeCN) to afford pure **29** (15 mg, 81%) as a colorless syrup. ^1^H NMR (500 MHz, CD_3_OD) δ 4.70 (dd, 1H, *J* = 2.7, *J* = 7.9 Hz, H-3′), 4.60 (d, 1H, *J* = 2.7, H-2′), 4.47–4.44 (m, 2H, H-1, 7a), 4.40 (t, 1H, *J* = 2.7 Hz, H-6), 4.30 (dd, 1H, *J* = 2.0, *J* = 7.9 Hz, H-4′), 4.16 (brs, 1H, H-2), 4.07 (dd, 1H, *J*_1_ = 2.0, *J*_2_ = 13.0 Hz, H-5′A), 3.87 (dd, 1H, *J*_1_ = 11.8, *J*_2_ = 9.4 Hz, CH_2_OBn_(A)_), 3.75 (d, 1H, *J* = 13.0 Hz, H-5′B), 3.74 (dd, 1H, *J*_1_ = 4.3, *J*_2_ = 11.8 Hz, CH_2_OBn_(B)_), 3.69 (m, 1H, H-5), 3.57 (m, 1H, H-3), 3.08 (dd, 1H, *J*_1_ = 2.7, *J*_2_ = 11.5 Hz, H-7), 1.56, 1.50, 1.47, and 1.37 (4s, 12H, 2CMe_2_), 1.41 (d, 3H, *J* = 6.7 Hz, CH_3_); ^13^C NMR (125 MHz, CD_3_OD) δ 109.07 and 109.05 (2CMe_2_), 102.7 (C-1′), 79.6 (CH-2), 78.9 (CH-1), 74.4 (CH-7a), 73.5 (CH-6), 72.1 (CH-2′), 71.0 (CH-5), 70.4 (CH-4′), 70.0 (CH-3′), 61.3 (CH_2_-5′), 59.3 (CH_2_OBn), 54.0 (CH-7), 25.5, 24.82, 24.75, and 22.4 (2CMe_2_), 9.9 (CH_3_); HRMS (TOF ESI^+^, *m*/*z*) 432.2237 [M + H]^+^, calcd for C_20_H_34_NO_9_ 432.2234.

## Supplementary Material



## Notes

Complementary experimental details, discussion on NOESY experiments, and copies of NMR spectra (PDF)

## References

[1] Asano N., Nash R. J., Molyneux R. J., Fleet G. W. J. (2000). Sugar-Mimic Glycosidase
Inhibitors: Natural Occurrence, Biological Activity and Prospects
for Therapeutic Application. Tetrahedron Asymmetry.

[2] Watson A. A., Fleet G. W. J., Asano N., Molyneux R. J., Nash R. J. (2001). Polyhydroxylated
Alkaloids — Natural Occurrence and Therapeutic Applications. Phytochemistry.

[3] Compain, P. ; Martin, O. R. Iminosugars: From Synthesis to Therapeutic Applications. John Wiley & Sons, 2007, pp. 496.

[4] Horne G., Wilson F. X., Tinsley J., Williams D. H., Storer R. (2011). Iminosugars
Past, Present and Future: Medicines for Tomorrow. Drug. Discovery Today.

[5] Nash R. J., Kato A., Yu C. Y., Fleet G. W. (2011). Iminosugars as Therapeutic
Agents: Recent Advances and Promising Trends. Future Med. Chem..

[6] Stütz, A. E. ; Paulsen, H. Iminosugars as Glycosidase Inhibitors: Nojirimycin and Beyond; Wiley-VCH, 1999; p 411.

[7] Borges
de Melo E., da Silveira Gomes A., Carvalho I. (2006). α- and β-Glucosidase
Inhibitors: Chemical Structure and Biological Activity. Tetrahedron.

[8] Compain P., Chagnault V., Martin O. R. (2009). Tactics and Strategies for the Synthesis
of Iminosugar C-Glycosides: A Review. Tetrahedron
Asymmetry.

[9] D’Alonzo D., Guaragna A., Palumbo G. (2009). Glycomimetics at the
Mirror: Medicinal
Chemistry of L-Iminosugars. Curr. Med. Chem..

[10] Brás N. F., Cerqueira N. M. F. S. A., Ramos M. J., Fernandes P. A. (2014). Glycosidase
Inhibitors: A Patent Review (2008 – 2013). Expert Opin. Ther. Pat..

[11] Alonzi D. S., Scott K. A., Dwek R. A., Zitzmann N. (2017). Iminosugar Antivirals:
The Therapeutic Sweet Spot. Biochem. Soc. Trans..

[12] Kato A., Kato N., Adachi I., Hollinshead J., Fleet G. W. J., Kuriyama C., Ikeda K., Asano N., Nash R. J. (2007). Isolation of Glycosidase-Inhibiting Hyacinthacines
and Related Alkaloids from Scilla Socialis. J. Nat. Prod..

[13] Ritthiwigrom T., Au W. G., C G. P. (2012). Structure, Biological Activities
and Synthesis of Hyacinthacine Alkaloids and Their Stereoisomers. Curr. Org. Synth..

[14] Pecchioli T., Cardona F., Reissig H. U., Zimmer R., Goti A. (2017). Alkoxyallene-Based
Stereodivergent Syntheses of (−)-Hyacinthacine B4 and of Putative
Hyacinthacine C5 Epimers: Proposal of Hyacinthacine C5 Structure. J. Org. Chem..

[15] Kato A., Kano E., Adachi I., Molyneux R. J., Watson A. A., Nash R. J., Fleet G. W. J., Wormald M. R., Kizu H., Ikeda K., Asano N. (2003). Australine
and Related Alkaloids:
Easy Structural Confirmation by 13C NMR Spectral Data and Biological
Activities. Tetrahedron Asymmetry.

[16] Cardona F., Parmeggiani C., Faggi E., Bonaccini C., Gratteri P., Sim L., Gloster T. M., Roberts S., Davies G. J., Rose D. R. (2009). Total Syntheses of Casuarine
and Its 6-O-α-Glucoside: Complementary Inhibition towards Glycoside
Hydrolases of the GH31 and GH37 Families. Chem.—Eur.
J..

[17] Bonaccini C., Chioccioli M., Parmeggiani C., Cardona F., Lo Re D., Soldaini G., Vogel P., Bello C., Goti A., Gratteri P. S. (2010). Biological
Evaluation and Docking Studies of Casuarine
Analogues: Effects of Structural Modifications at Ring B on Inhibitory
Activity towards Glucoamylase. Eur. J. Org.
Chem..

[18] Cardona F., Goti A., Parmeggiani C., Parenti P., Forcella M., Fusi P., Cipolla L., Roberts S. M., Davies G. J., Gloster T. M. (2010). Casuarine-6-O-α-D-Glucoside and Its Analogues
Are Tight Binding Inhibitors of Insect and Bacterial Trehalases. Chem. Commun..

[19] Hattie M., Ito T., Debowski A. W., Arakawa T., Katayama T., Yamamoto K., Fushinobu S., Stubbs K. A. (2015). Gaining Insight into the Catalysis
by GH20 Lacto-N-Biosidase Using Small Molecule Inhibitors and Structural
Analysis †. Chem. Commun..

[20] Asano N., Ikeda K., Kasahara M., Arai Y., Kizu H. (2004). Glycosidase-Inhibiting
Pyrrolidines and Pyrrolizidines with a Long Side Chain in Scilla Peruviana. J. Nat. Prod..

[21] Usuki H., Toyo-Oka M., Kanzaki H., Okuda T., Nitoda T. (2009). Pochonicine,
a Polyhydroxylated Pyrrolizidine Alkaloid from Fungus Pochonia Suchlasporia
Var. Suchlasporia TAMA 87 as a Potent β-N-Acetylglucosaminidase
Inhibitor. Bioorg. Med. Chem..

[22] Robertson J., Stevens K. (2017). Pyrrolizidine Alkaloids: Occurrence, Biology, and Chemical
Synthesis. Nat. Prod. Rep..

[23] Rück-Braun K., Freysoldt T. H. E., Wierschem F. (2005). 1,3-Dipolar Cycloaddition on Solid
Supports: Nitrone Approach towards Isoxazolidines and Isoxazolines
and Subsequent Transformations. Chem. Soc. Rev..

[24] Yokoshima S. (2025). Intramolecular
Cycloaddition of Nitrones in Total Synthesis of Natural Products. Nat. Prod. Rep..

[25] Esteban A., Ortega P., Sanz F., Jambrina P. G., Díez D. (2024). Crystallization-Induced
Diastereomer Transformation Assists the Diastereoselective Synthesis
of 4-Nitroisoxazolidine Scaffolds. Org. Biomol.
Chem..

[26] Liu H., Zhao Y., Li Z., Jia H., Zhang C., Xiao Y., Guo H. (2017). Lewis Base-Catalyzed Diastereoselective
[3 + 2] Cycloaddition Reaction of Nitrones with Electron-Deficient
Alkenes: An Access to Isoxazolidine Derivatives. RSC Adv..

[27] Januário M.-O., Charmier J., Moussalli N., Chanet-Ray J., Chou S. (1999). 1 3,Dipolar Cycloaddition Reactions of Nitrones with Unsaturated
Methylsulfones and Substituted Crotonic Esters. J. Chem. Res..

[28] Banerji A., Biswas P. K., Bandyopadhyay D., Gupta M., Prangé T., Neuman A. (2007). 1 3,Dipolar Cycloadditions:
Investigation of Cycloadditions
of c-Aryl-n-(4-Chlorophenyl) Nitrones to n-Cinnamoyl Piperidines. J. Heterocycl. Chem..

[29] Brandi A., Cardona F., Cicchi S., Cordero F. M., Goti A. (2017). [3+ 2] Dipolar
Cycloadditions of Cyclic Nitrones with Alkenes. Org. React..

[30] Esteban A., Nieto C. T., Garrido N. M., Sanz F., Díez D. (2025). Regiodivergence
in the Cycloadditions between a Cyclic Nitrone and Carbonyl-Type Dipolarophiles. J. Org. Chem..

[31] Tamura O., Toyao A., Ishibashi H. (2002). TBAT-Mediated
Nitrone Formation of
ω-Mesyloxy-O-Tert-Butyldiphenylsilyloximes: Facile Synthesis
of Cyclic Nitrones from Hemiacetals. Synlett.

[32] Ishikawa T., Tajima Y., Fukui M., Saito S. (1996). Synthesis and Asymmetric
[3 + 2] Cycloaddition Reactions of Chiral Cyclic Nitrone: A Novel
System Providing Maximal Facial Bias for Both Nitrone and Dipolarophile. Angew. Chem., Int. Ed..

[33] Sayed, E. ; El Ashry, H. ; Heterocycles from Carbohydrate Precursors. Springer, 2007, 354.

[34] Brandi A., Cardona F., Cicchi S., Cordero F. M., Goti A. (2009). Stereocontrolled
Cyclic Nitrone Cycloaddition Strategy for the Synthesis of Pyrrolizidine
and Indolizidine Alkaloids. Chem.—Eur.
J..

[35] Hall A., Meldrum K. P., Therond P. R., Wightman R. H. (1997). Synthesis of Hydroxylated
Pyrrolizidines Related to Alexine Using Cycloaddition Reactions of
Functionalized Cyclic Nitrones. Synlett.

[36] Goti A., Cacciarini M., Cardona F., Brandi A. (1999). A Convenient Access
to (3S)-3-(Triisopropylsilyl)­Oxy-1-Pyrroline N-Oxide, a Useful Intermediate
for Polyfunctionalized Enantiopure Indolizidine and Pyrrolizidine
Synthesis. Tetrahedron Lett..

[37] Schieweck F., Altenbach H. J. (2001). Synthesis
of Geminal Bis­(Hydroxymethyl)­Pyrrolidine
and Pyrrolizidine Imino Sugars. J. Chem. Soc.,
Perkin Trans..

[38] Tamayo J.
A., Franco F., Re D. L., Sánchez-Cantalejo F. (2009). Synthesis
of Pentahydroxylated Pyrrolizidines and Indolizidines. J. Org. Chem..

[39] Martella D., Cardona F., Parmeggiani C., Franco F., Tamayo J. A., Robina I., Moreno-Clavijo E., Moreno-Vargas A. J., Goti A. (2013). Synthesis and Glycosidase Inhibition Studies of 5-Methyl-Substituted
Tetrahydroxyindolizidines and -Pyrrolizidines Related to Natural Hyacinthacines
B. Eur. J. Org. Chem..

[40] D’Adamio G., Parmeggiani C., Goti A., Moreno-Vargas A. J., Moreno-Clavijo E., Robina I., Cardona F. (2014). 6-Azido Hyacinthacine
A2 Gives a Straightforward Access to the First Multivalent Pyrrolizidine
Architectures. Org. Biomol Chem..

[41] Beňadiková D., Medvecký M., Filipová A., Moncol’ J., Gembický M., Prónayová N., Fischer R. (2014). New Synthetic
Approach to C5-Hydroxymethyl-Substituted Polyhydroxylated Pyrrolizidines
Hydroxymethyl-Substituted Polyhydroxylated Pyrrolizidines. Synlett.

[42] Lahiri R., Palanivel A., Kulkarni S. A., Vankar Y. D. (2014). Synthesis of Isofagomine–Pyrrolidine
Hybrid Sugars and Analogues of (−)-Steviamine and (+)-Hyacinthacine
C5 Using 1,3-Dipolar Cycloaddition Reactions. J. Org. Chem..

[43] Martella D., D’Adamio G., Parmeggiani C., Cardona F., Moreno-Clavijo E., Robina I., Goti A. (2016). Cycloadditions of Sugar-Derived Nitrones
Targeting Polyhydroxylated Indolizidines. Eur.
J. Org. Chem..

[44] Izquierdo I., Plaza M. T., Robles R., Rodríguez C. (1996). A Highly Diastereoselective
Synthesis of 4-Octulose and 2-Deoxy-4-Octulose from a d-Fructose Derivative. Tetrahedron Asymmetry.

[45] Cubero I. I., Lopez-Espinosa M. T. P., Richardson A. C. (1993). Enantiospecific Synthesis from D-Fructose
of (2 S,5 R)- and (2 R,5 R)-2-Methyl-1,6-Dioxaspiro[4.5]­Decane [the
Odor Bouquet Minor Components of Paravespula Vulgaris (L.)]. J. Chem. Ecol..

[46] Cardona F., Faggi E., Liguori F., Cacciarini M., Goti A. (2003). Total Syntheses of Hyacinthacine
A2 and 7-Deoxycasuarine by Cycloaddition
to a Carbohydrate Derived Nitrone. Tetrahedron
Lett..

[47] Cicchi S., Marradi M., Vogel P., Goti A. (2006). One-Pot Synthesis
of
Cyclic Nitrones and Their Conversion to Pyrrolizidines: 7a-Epi-Crotanecine
Inhibits α-Mannosidases. J. Org. Chem..

[48] Gkizis P., Argyropoulos N. G., Coutouli-Argyropoulou E. (2013). A Sort Synthesis of
Polyhydroxylated Pyrrolizidines via Sequential 1,3-Dipolar Cycloaddition
and Reductive Amination. Tetrahedron.

[49] Li Y. X., Wang J. Z., Shimadate Y., Kise M., Kato A., Jia Y. M., Fleet G. W. J., Yu C. Y. (2022). Diastereoselective
Synthesis, Glycosidase Inhibition, and Docking Study of C-7-Fluorinated
Casuarine and Australine Derivatives. J. Org.
Chem..

[50] Coutouli-Argyropoulou E., Xatzis C., Argyropoulos N. G. (2008). Application of Chiral Cyclic Nitrones
to the Diastereoselective Synthesis of Bicyclic Isoxazolidine Nucleoside
Analogues. Nucleosides Nucleotides Nucleic Acids.

[51] Podolan G., Kleščíková L., Fiera L., Kožíšek J., Fronc M. (2011). Efficient Synthesis of Tetrahydroxylated Pyrrolizidines by Nitrone
Cycloaddition Leading to Unnatural Stereoisomers of 7-Deoxycasuarine. Synlett.

[52] Majer R., Konechnaya O., Delso I., Tejero T., Attanasi O. A., Santeusanio S., Merino P. (2014). Highly Diastereoselective 1,3-Dipolar
Cycloadditions of Chiral Non-Racemic Nitrones to 1,2-Diaza-1,3-Dienes:
An Experimental and Computational Investigation. Org. Biomol. Chem..

[53] Goti A., Cicchi S., Cacciarini M., Cardona F., Fedi V., Brandi A. (2000). Straightforward Access
to Enantiomerically Pure, Highly
Functionalized Pyrrolizidines by Cycloaddition of Maleic Acid Esters
to Pyrroline N-Oxides Derived from Tartaric, Malic and Aspartic Acids
Synthesis of (−)-Hastanecine, 7-Epi-Croalbinecine and (−)-Croalbinecine. Eur. J. Org. Chem..

[54] Izquierdo I., Plaza M. T., Tamayo J. A. P. P. (2005). Part
6: A New and Concise Stereoselective
Synthesis of (+)-Casuarine and Its 6,7-Diepi Isomer, from DMDP. Tetrahedron.

[55] de
Wit G., de Hann C., Kieboom A. P. G., van Bekkum H. (1980). Enediol-Anion
Formation and β-Elimination of Cyclic α-Hydroxycarbonyl
Compounds as Studied by u.v. and n.m.r. Spectroscopy. Carbohydr. Res..

[56] Cicchi S., Goti A., Brandi A., Guarna A., De Sarlo F. (1990). 1,3-Aminoalcohols
by Reductive Cleavage of Isoxazolidines with Molybdenum Hexacarbonyl. Tetrahedron Lett..

[57] Cordero F. M., Brandi A., Cristilli S., De Sarlo F., Viti F. G. (1994). Syntheses
of 3-Phenyl Substituted Indolizidin-2-Ones and a Pyrrolizidin-2-One
on the Route to Constrained Potential NK1 Receptor Antagonists. Tetrahedron.

