# Supplementary material for: Stereoselective Synthesis of C‑Glycosylated Pyrrolizidines through Nitrone Cycloadditions
Source: ACS Omega. 2026 Jan 7;11(2):3412–22. doi: 10.1021/acsomega.5c10431 (PMC12824959; doi:10.1021/acsomega.5c10431)

Stereoselective synthesis of C-Glycosylated Pyrrolizidines through Nitrone Cycloadditions

Francisco Franco,[a] Mónica Díaz-Gavilán,[a] Daniele Lo Re,*[a] and Juan A. Tamayo*[a]

Table of contents:

1. General experimental methods S3

2. Tridimensional structure of bicyclic compounds: NOESY experiments S4

3. NMR spectra S7-S59

General Experimental Methods

^{1}H-NMR and ^{13}C-NMR spectra have been recorded in 400, 500 and 600 MHz spectrometers. Chemical shifts (δ) are quoted in ppm and are referenced to residual H in the deuterated solvent as the internal standard. Coupling constants (*J*) are expressed in Hz. Splitting patterns are designated as follows: s, singlet; d, doublet; t, triplet; q, quartet; quintet, quintet; m, multiplet and br, broad. High resolution mass spectra (HRMS) were recorded by liquid secondary ionization (LSIMS) in thioglycerol or NOBA matrix, or by time of flight (TOF) mass spectrometry with electrospray ionization (ESI) in positive mode. Low resolution mass spectra (LRMS) were recorded by ESI coupled to high performance liquid chromatography (LC-MS). Small scale microwave-assisted synthesis was carried out in sealed vessels using an Initiator 2.0 single-mode microwave instrument producing controlled irradiation at 2.450 GHz. Optical rotations were measured for solutions in $CHCl_3$ (1 dm tube). Reactions were controlled by thin layer chromatography (TLC) on aluminium plates (Merck AL, Silicagel 60 F254) and detected using a mixture of 10% ammonium molybdate (w/v) in 10% aqueous sulfuric acid containing 0.8% cerium sulfate (w/v) and heating. *R*f values refer to these TLC plates developed in the solvents indicated. The non-crystalline compounds were shown to be homogeneous by chromatographic methods and characterized by NMR and HRMS. Purification by flash chromatography was performed on Silicagel Merck 60 (230-400 mesh ASTM). Anhydrous solvents were obtained from commercial sources.

2. Tridimensional structure of bicyclic compounds: NOESY experiments

The tridimensional structure has been determined on the intermediates along the synthetic route from NOE interactions (NOESY experiments).

The relative configurations of C-2 and C-3 on the isoxazolidines are determined by the *endo/exo* stereochemistry of the cycloaddition. In the case of **22a**, an *exo* orientation of the carboxylate group was deduced from the NOE interactions observed between H-2 with H-4 and H-2 with H-6. This assignment was further supported by correlations observed in subsequent derivatives, specifically between H-2 and H-7a.

For compound **22b**, an *endo* orientation of the carboxylate group was deduced from the NOE correlation between H-3 and H-6. The same type of spatial correlation was later confirmed in derivatives **26** (H-1 with H-7) and **16** (H-1 with H-7).

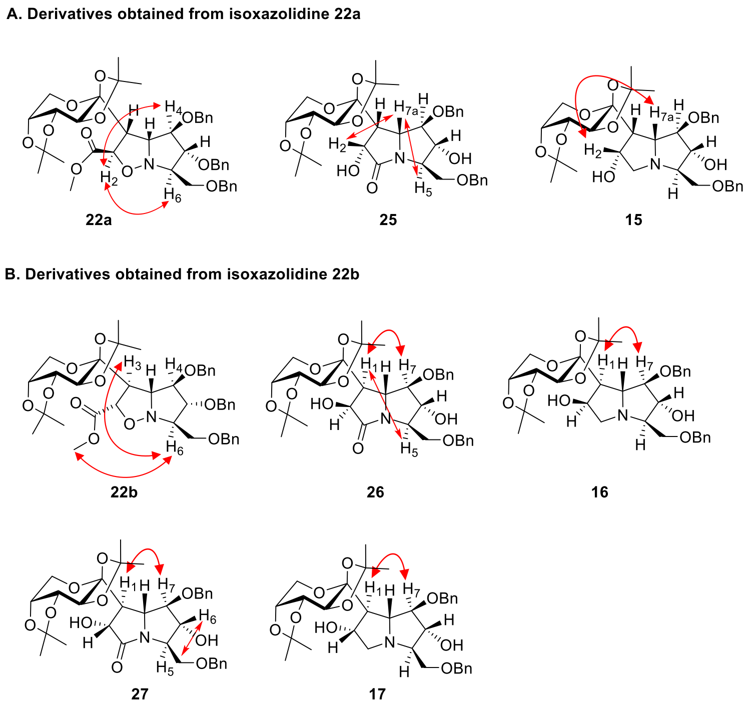

Figure S1. NOE-based stereochemical analysis supporting the formation of pyrrolizidines **15**, **16**, and **17** from cycloadducts **22a** and **22b**.

When the cycloaddition is performed using the ketone 2**0b**, the resulting isoxazolidine **23** displays an *endo* orientation of the acetyl group, as indicated by the NOE interaction observed between H-3 and H-

4. This stereochemical assignment is further confirmed in the subsequent derivatives **28** and **18**, where similar interactions between H-1 and H-7, as well as H-3 and H-5, are observed.

The stereochemistry of H-2 in the two compounds obtained from the reduction of **28** was determined by NOE interactions: between H-2 and the methyl group in **18**, and between H-2 and H-5 in **19**.

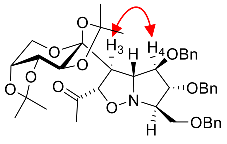

23

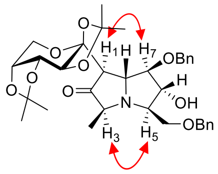

28

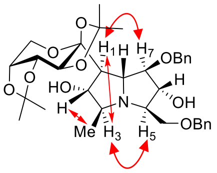

18

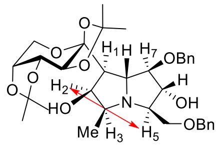

19

Figure S2. NOE-based stereochemical analysis supporting the formation of pyrrolizidines **18** and **19** from cycloadducts **23**.

3. NMR spectra

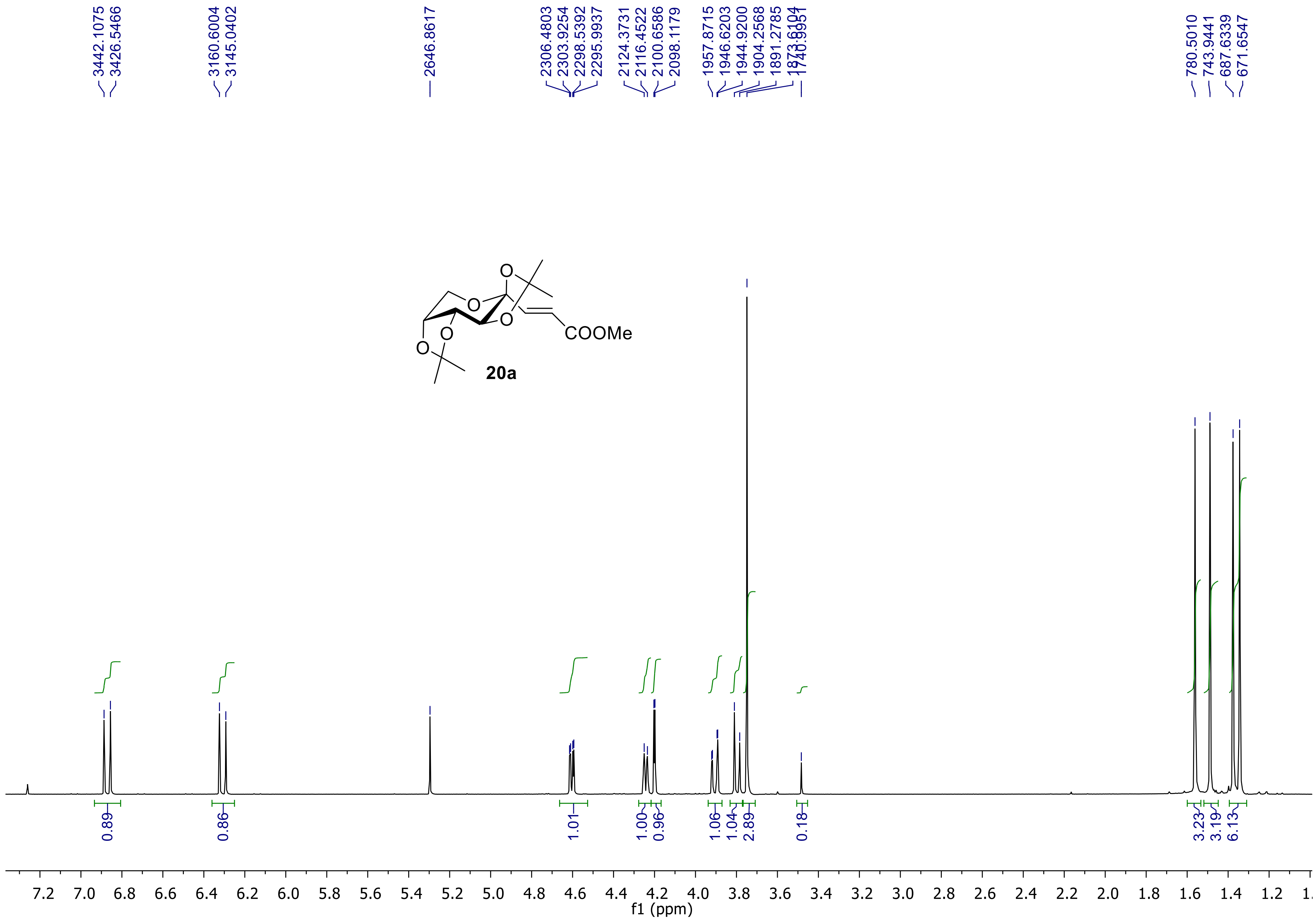

3442.1075
3426.5466
3160.6004
3145.0402
2646.8617
2306.4803
2303.9254
2298.5392
2295.9937
2124.3731
2116.4522
2100.6586
2098.1179
1957.8715
1946.6203
1944.9200
1904.2568
1891.2785
1873.6104
1740.9951
780.5010
743.9441
687.6339
671.6547
O
O
O
O
O
O
COOMe
20a
0.89
0.86
1.01
1.00
0.96
1.06
1.04
2.89
0.18
3.23
3.19
6.13
7.2
7.0
6.8
6.6
6.4
6.2
6.0
5.8
5.6
5.4
5.2
5.0
4.8
4.6
4.4
4.2
4.0
3.8
3.6
3.4
3.2
3.0
2.8
2.6
2.4
2.2
2.0
1.8
1.6
1.4
1.2
f1 (ppm)

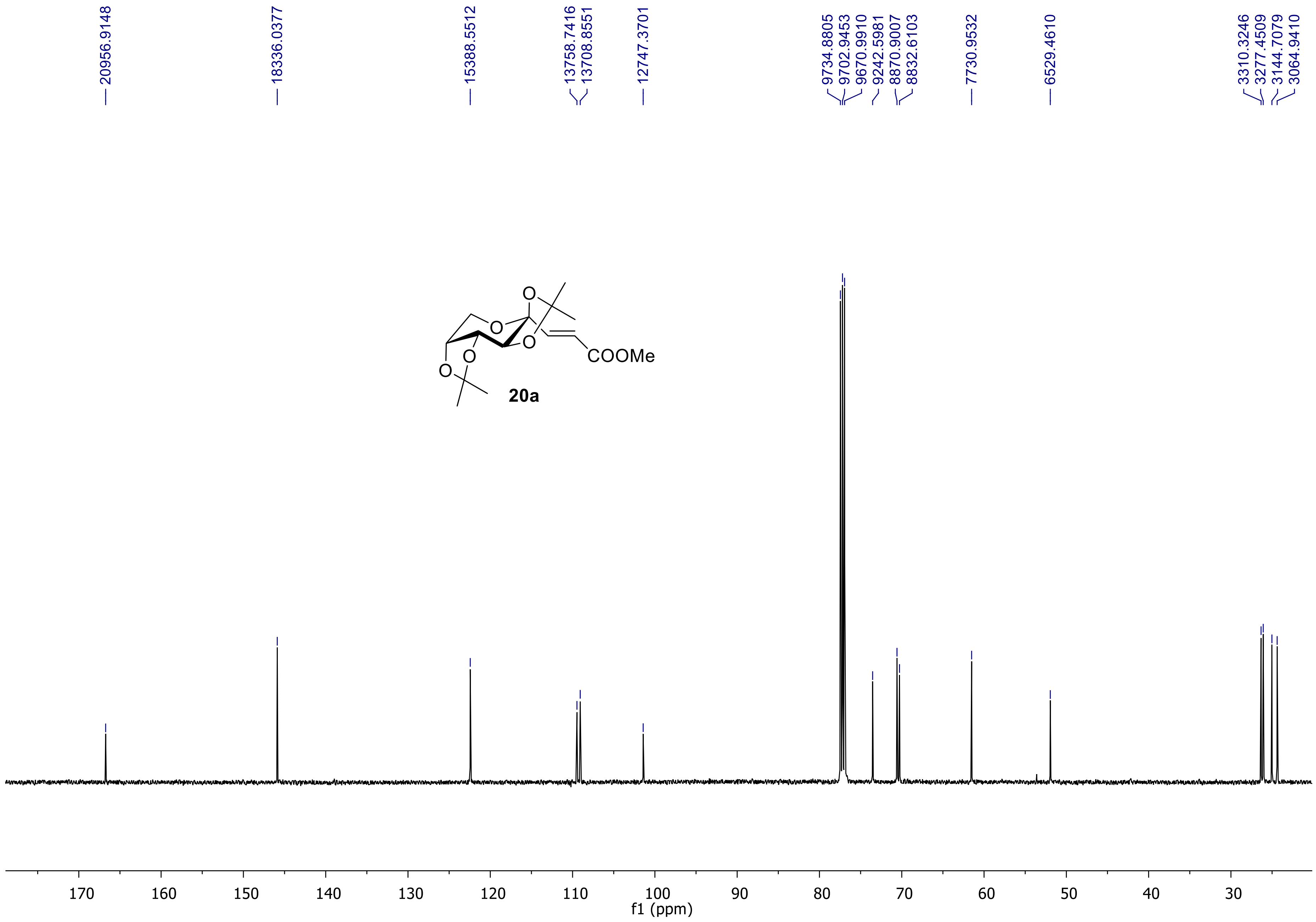
20956.9148
18336.0377
15388.5512
13758.7416
13708.8551
12747.3701
9734.8805
9702.9453
9670.9910
9242.5981
8870.9007
8832.6103
7730.9532
6529.4610
3310.3246
3277.4509
3144.7079
3064.9410
O
O
O
O
O
COOMe
20a
170
160
150
140
130
120
110
100
90
80
70
60
50
40
30
f1 (ppm)

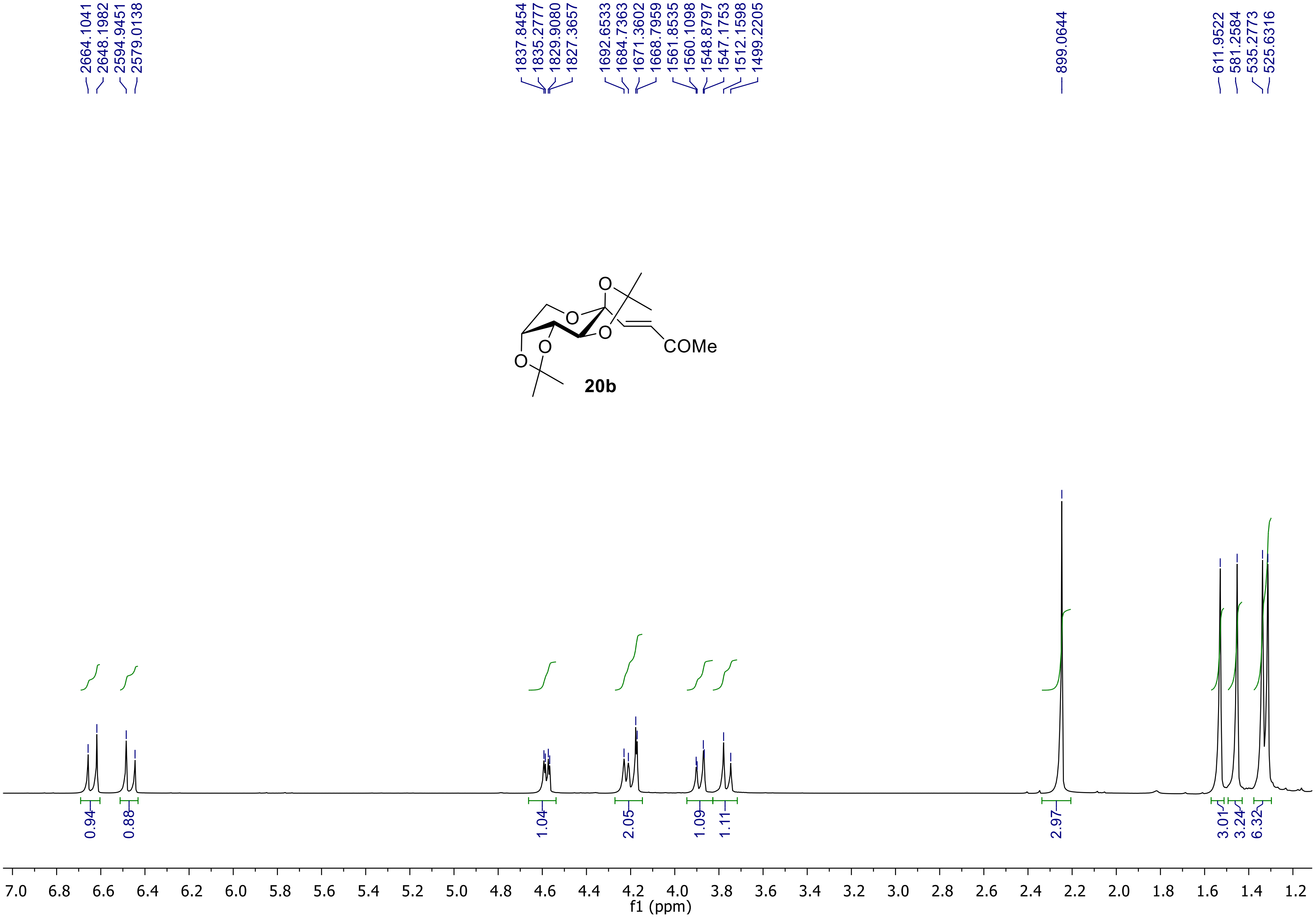

2664.1041
2648.1982
2594.9451
2579.0138
1837.8454
1835.2777
1829.9080
1827.3657
1692.6533
1684.7363
1671.3602
1668.7959
1561.8535
1560.1098
1548.8797
1547.1753
1512.1598
1499.2205
899.0644
611.9522
581.2584
535.2773
525.6316
COMe
20b
0.94
0.88
1.04
2.05
1.09
1.11
2.97
3.01
3.24
6.32
7.0 6.8 6.6 6.4 6.2 6.0 5.8 5.6 5.4 5.2 5.0 4.8 4.6 4.4 4.2 4.0 3.8 3.6 3.4 3.2 3.0 2.8 2.6 2.4 2.2 2.0 1.8 1.6 1.4 1.2
f1 (ppm)

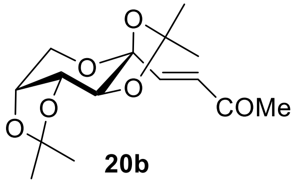
COMe

20b

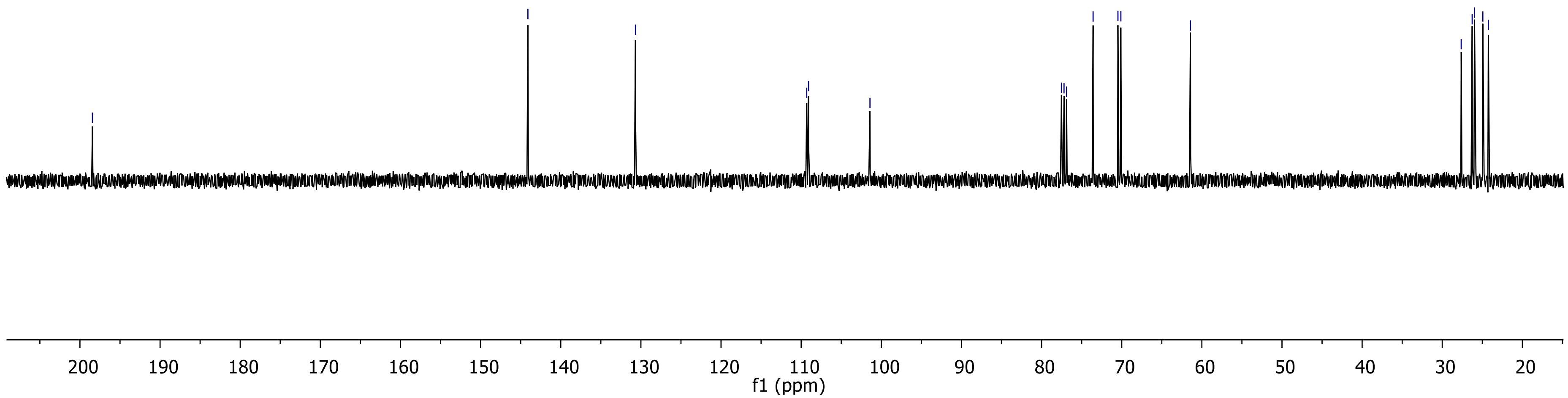
19969.3290
14501.0061
13150.4113
11000.0568
10976.7617
10205.5409
7801.0488
7768.8465
7736.9823
7403.6857
7091.6844
7055.0456
6181.3232
2780.8673
2643.3713
2614.2405
2509.5260
2440.0775
200 190 180 170 160 150 140 130 120 110 100 90 80 70 60 50 40 30 20
f1 (ppm)

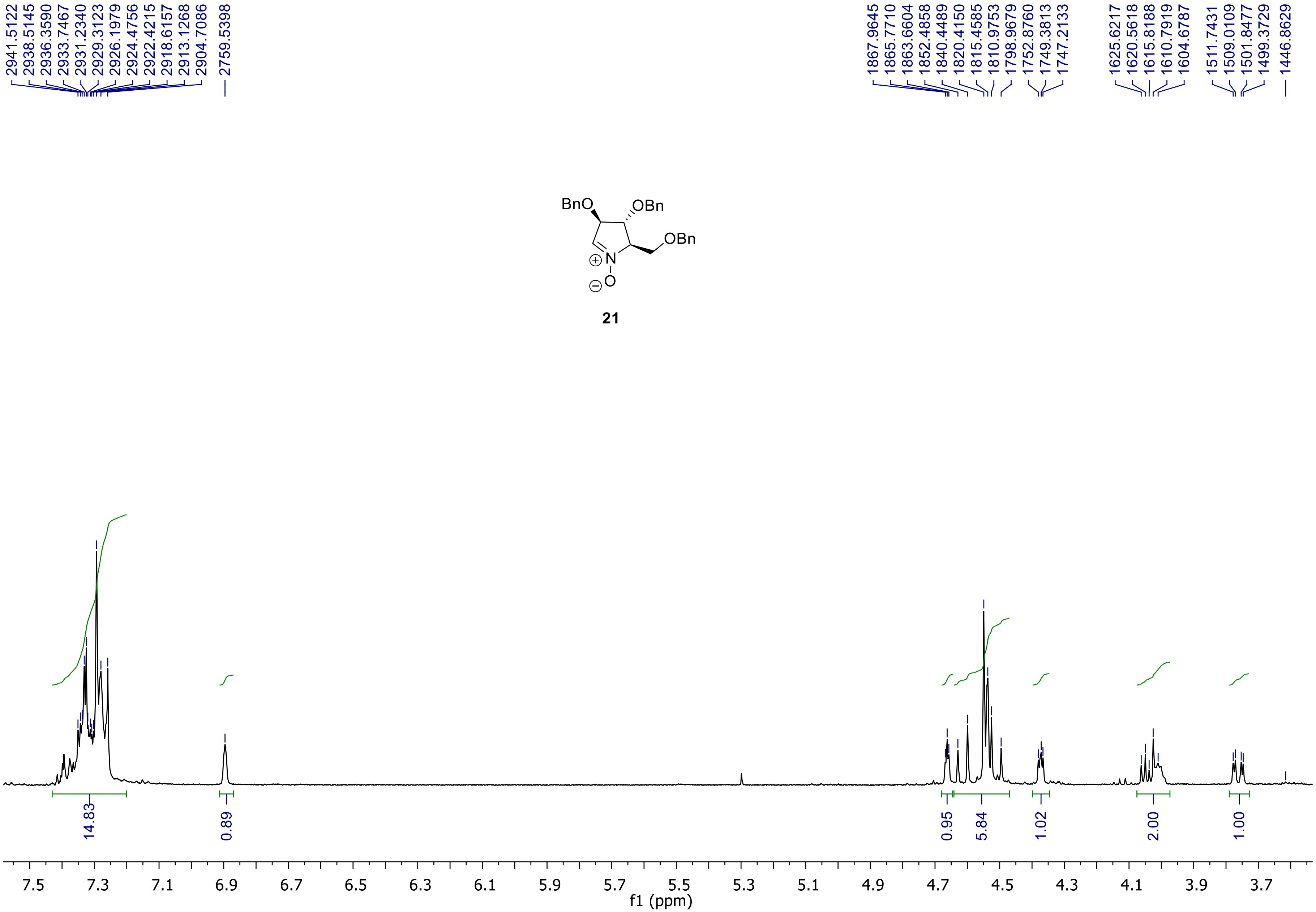
BnO
OBn
OBn
N
O
21
14.83
0.89
0.95
5.84
1.02
2.00
1.00
f1 (ppm)

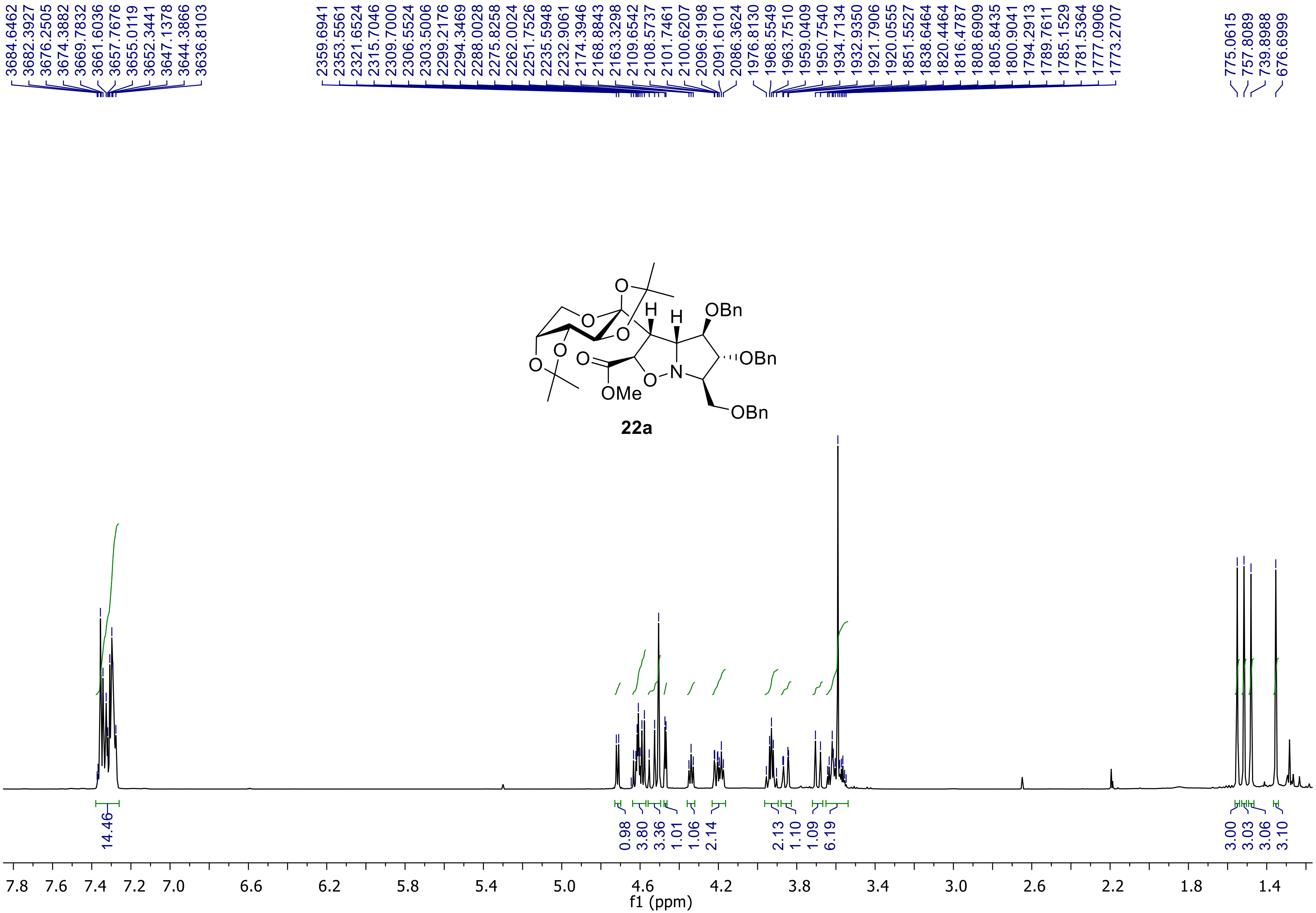
3684.6462
3682.3927
3676.2505
3674.3882
3669.7832
3661.6036
3657.7676
3655.0119
3652.3441
3647.1378
3644.3866
3636.8103
2359.6941
2353.5561
2321.6524
2315.7046
2309.7000
2306.5524
2303.5006
2299.2176
2294.3469
2288.0028
2275.8258
2262.0024
2251.7526
2235.5948
2232.9061
2174.3946
2168.8843
2163.3298
2109.6542
2108.5737
2101.7461
2100.6207
2096.9198
2091.6101
2086.3624
1976.8130
1968.5549
1963.7510
1959.0409
1950.7540
1934.7134
1932.9350
1921.7906
1920.0555
1851.5527
1838.6464
1820.4464
1816.4787
1808.6909
1805.8435
1800.9041
1794.2913
1789.7611
1785.1529
1781.5364
1777.0906
1773.2707
775.0615
757.8089
739.8988
676.6999
OBn
OBn
OBn
OMe
22a
14.46
0.98
3.80
3.36
1.01
1.06
2.14
2.13
1.10
1.09
6.19
3.00
3.03
3.06
3.10
7.8 7.6 7.4 7.2 7.0 6.6 6.2 5.8 5.4 5.0 4.6 4.2 3.8 3.4 3.0 2.6 2.2 1.8 1.4
f1 (ppm)

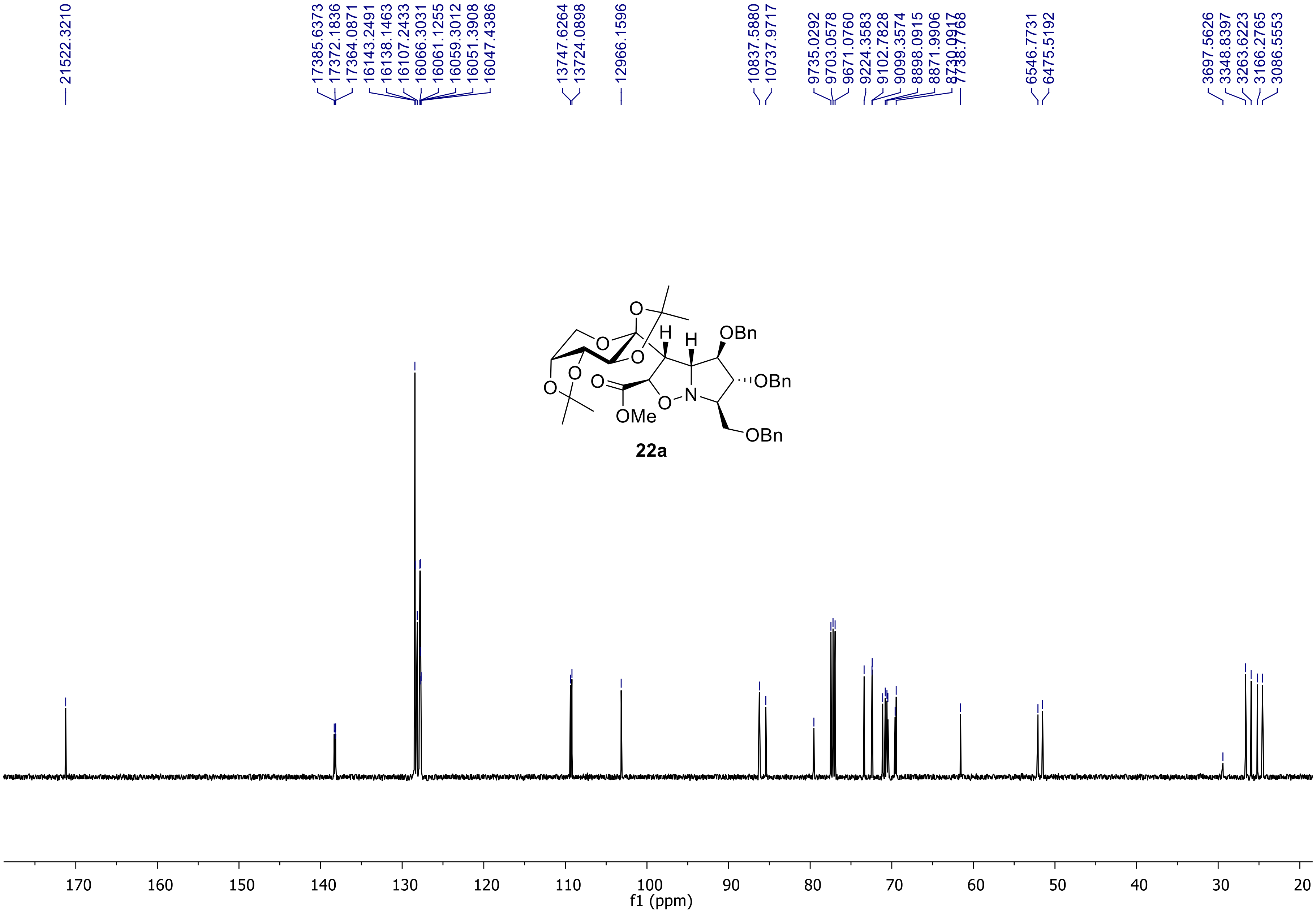
21522.3210
17385.6373
17372.1836
17364.0871
16143.2491
16138.1463
16107.2433
16066.3031
16061.1255
16059.3012
16051.3908
16047.4386
13747.6264
13724.0898
12966.1596
10837.5880
10737.9717
9735.0292
9703.0578
9671.0760
9224.3583
9102.7828
9099.3574
8898.0915
8871.9906
6546.7731
6475.5192
3697.5626
3348.8397
3263.6223
3166.2765
3086.5553
OBn
OBn
OBn
OMe
22a
170
160
150
140
130
120
110
100
90
80
70
60
50
40
30
20
f1 (ppm)

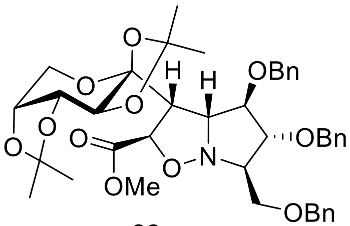

22a

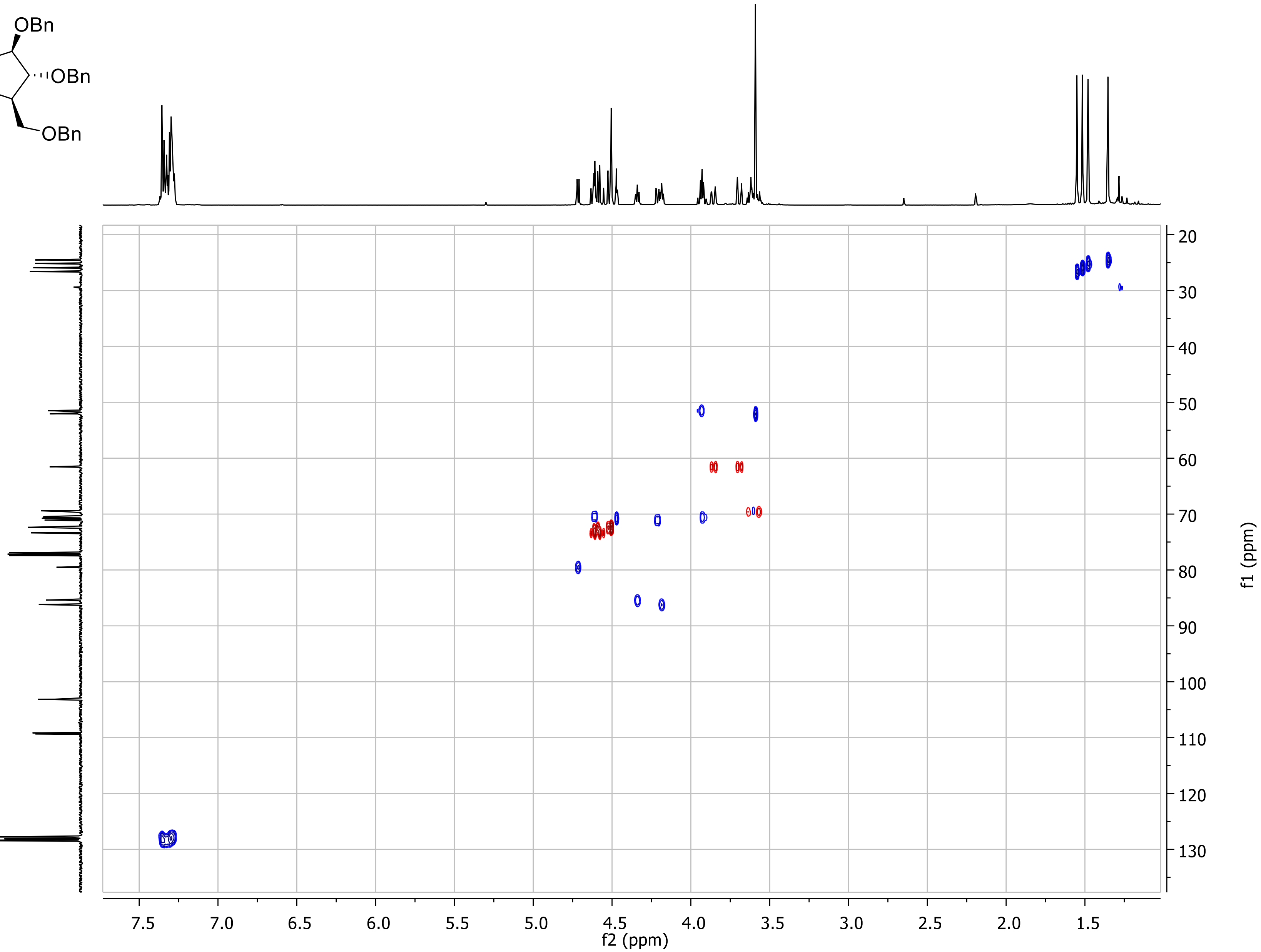

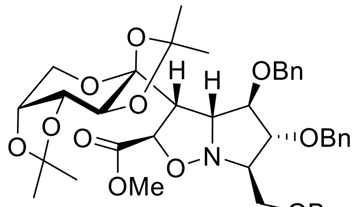

22a

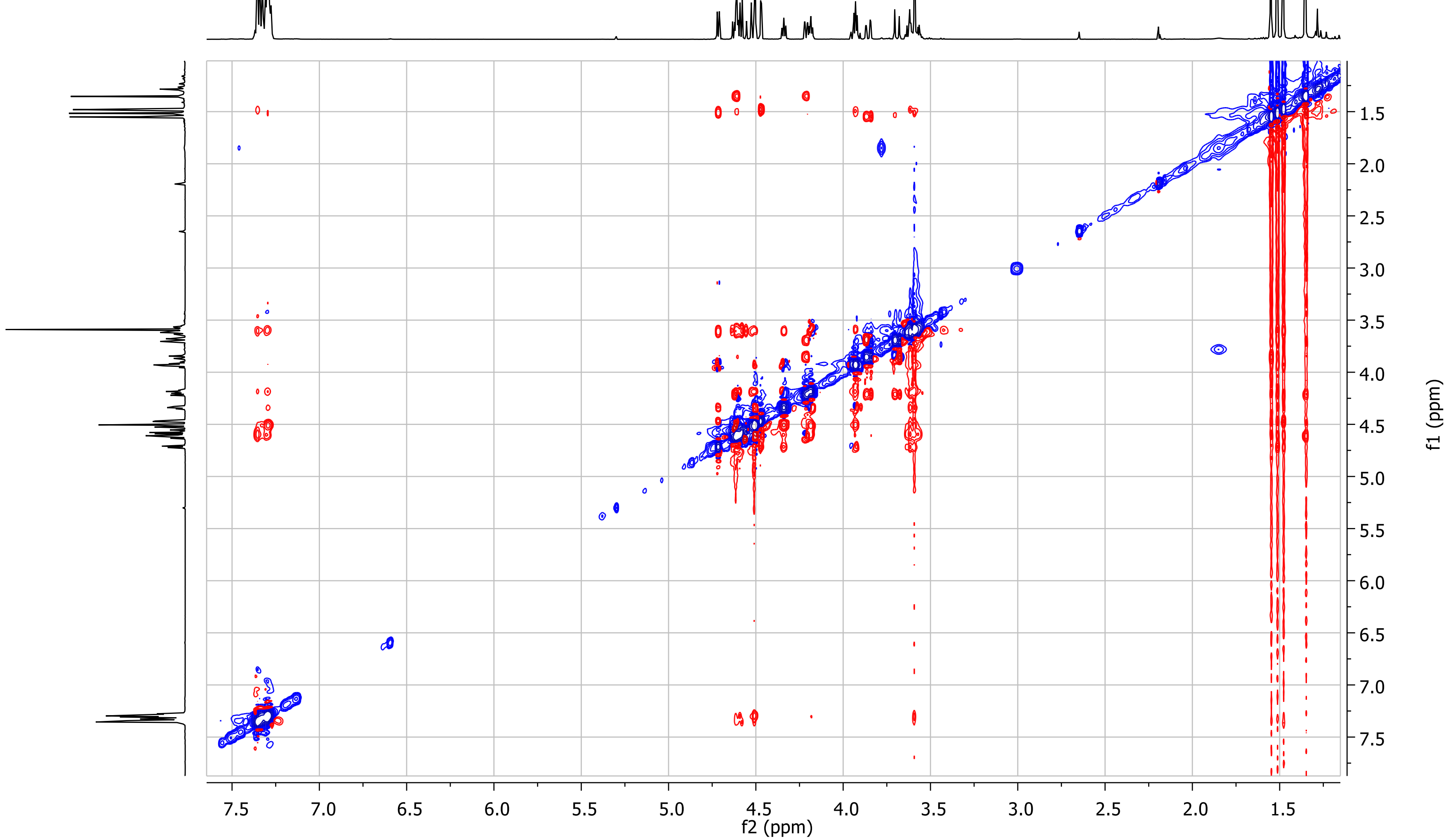

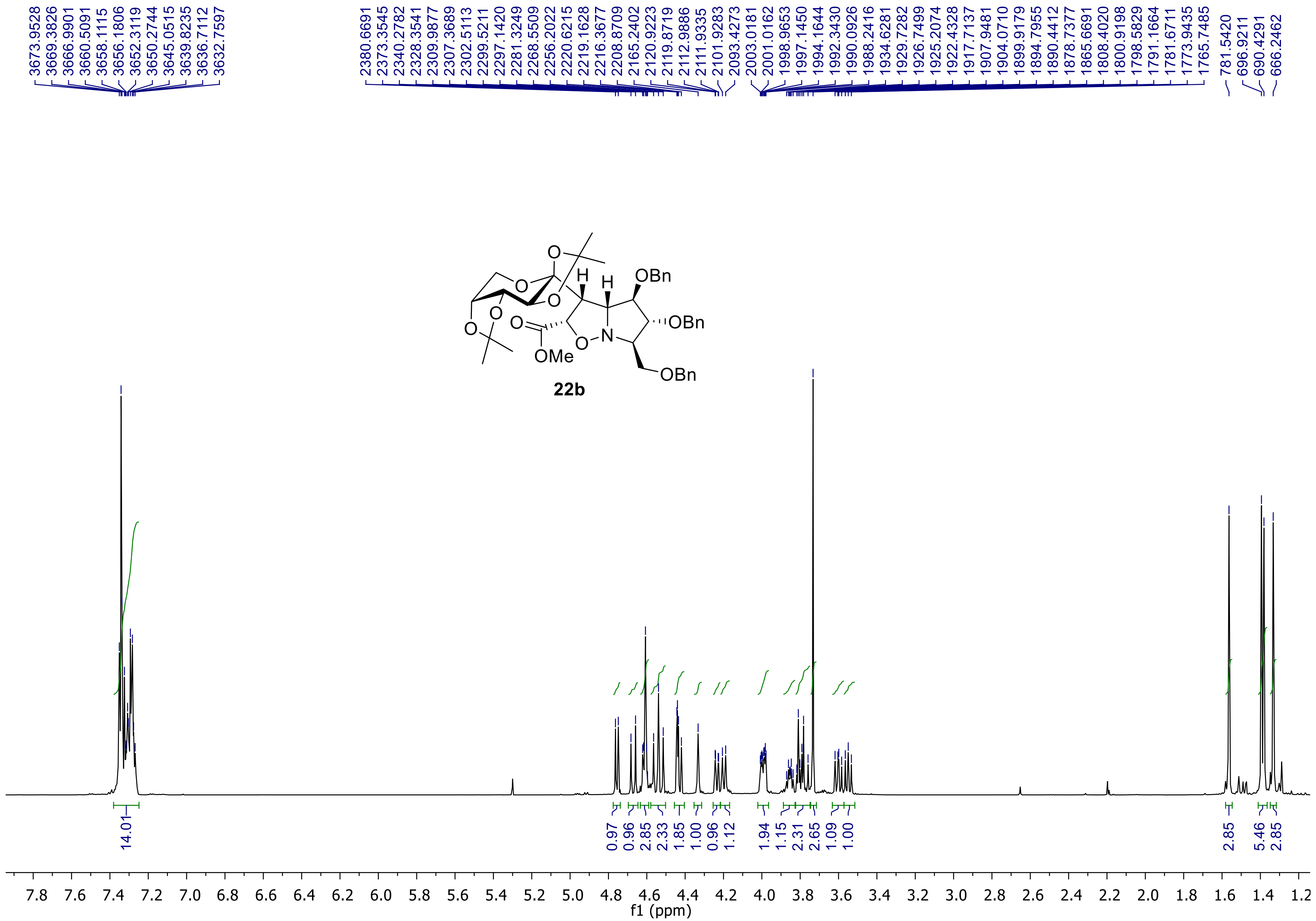
3673.9528
3669.3826
3666.9901
3660.5091
3658.1115
3656.1806
3652.3119
3650.2744
3645.0515
3639.8235
3636.7112
3632.7597
2380.6691
2373.3545
2340.2782
2328.3541
2309.9877
2307.3689
2302.5113
2299.5211
2297.1420
2281.3249
2268.5509
2256.2022
2220.6215
2219.1628
2216.3677
2208.8709
2165.2402
2120.9223
2119.8719
2112.9886
2111.9335
2101.9283
2093.4273
2003.0181
2001.0162
1998.9653
1997.1450
1994.1644
1992.3430
1990.0926
1988.2416
1934.6281
1929.7282
1926.7499
1925.2074
1922.4328
1917.7137
1907.9481
1904.0710
1899.9179
1894.7955
1890.4412
1878.7377
1865.6691
1808.4020
1800.9198
1798.5829
1791.1664
1781.6711
1773.9435
1765.7485
781.5420
696.9211
690.4291
666.2462
OBn
OBn
OBn
OMe
22b
14.01
0.97
0.96
2.85
2.33
1.85
1.00
0.96
1.12
1.94
1.15
2.31
2.65
1.09
1.00
2.85
5.46
2.85
7.8 7.6 7.4 7.2 7.0 6.8 6.6 6.4 6.2 6.0 5.8 5.6 5.4 5.2 5.0 4.8 4.6 4.4 4.2 4.0 3.8 3.6 3.4 3.2 3.0 2.8 2.6 2.4 2.2 2.0 1.8 1.6 1.4 1.2
f1 (ppm)

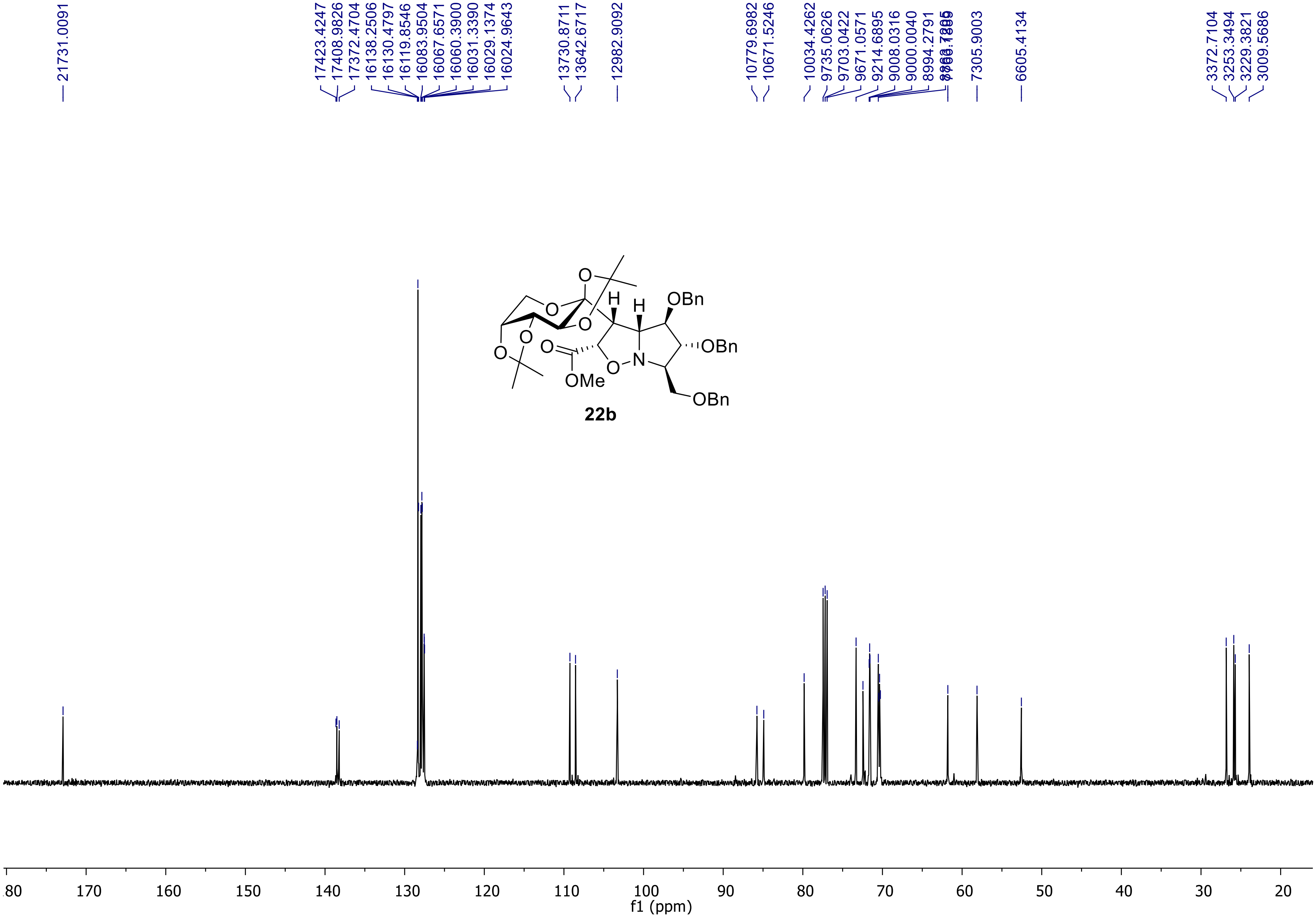
21731.0091
17423.4247
17408.9826
17372.4704
16138.2506
16130.4797
16119.8546
16083.9504
16067.6571
16060.3900
16031.3390
16029.1374
16024.9643
13730.8711
13642.6717
12982.9092
10779.6982
10671.5246
10034.4262
9735.0626
9703.0422
9671.0571
9214.6895
9008.0316
9000.0040
8994.2791
7305.9003
6605.4134
3372.7104
3253.3494
3229.3821
3009.5686
O
H
H
OBn
O
O
O
O
OBn
O
N
O
OMe
OBn
22b
80
170
160
150
140
130
120
110
100
90
80
70
60
50
40
30
20
f1 (ppm)

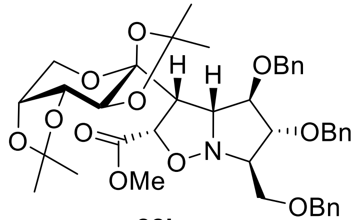

22b

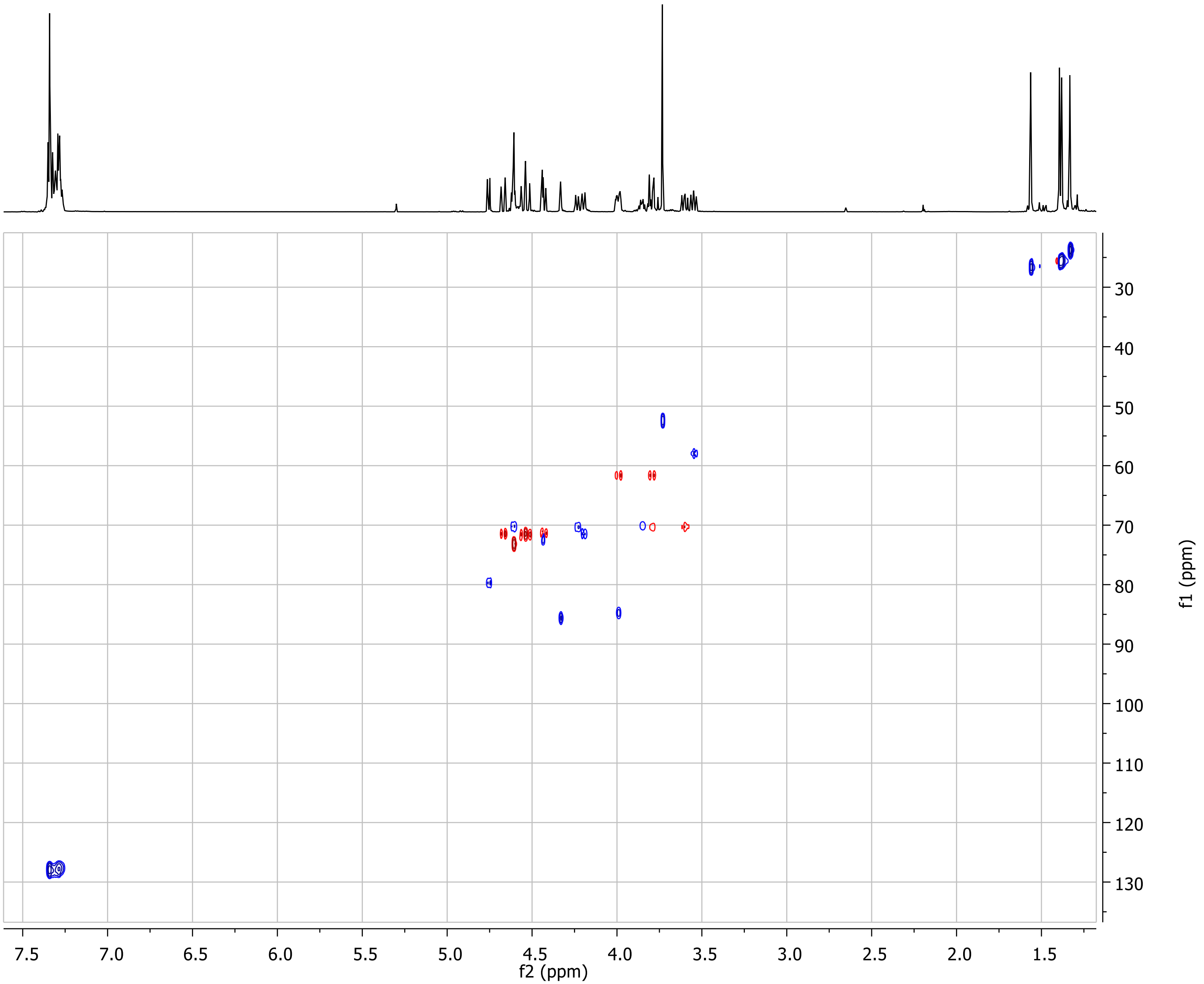

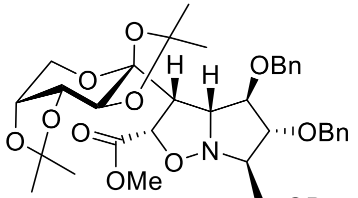

22b

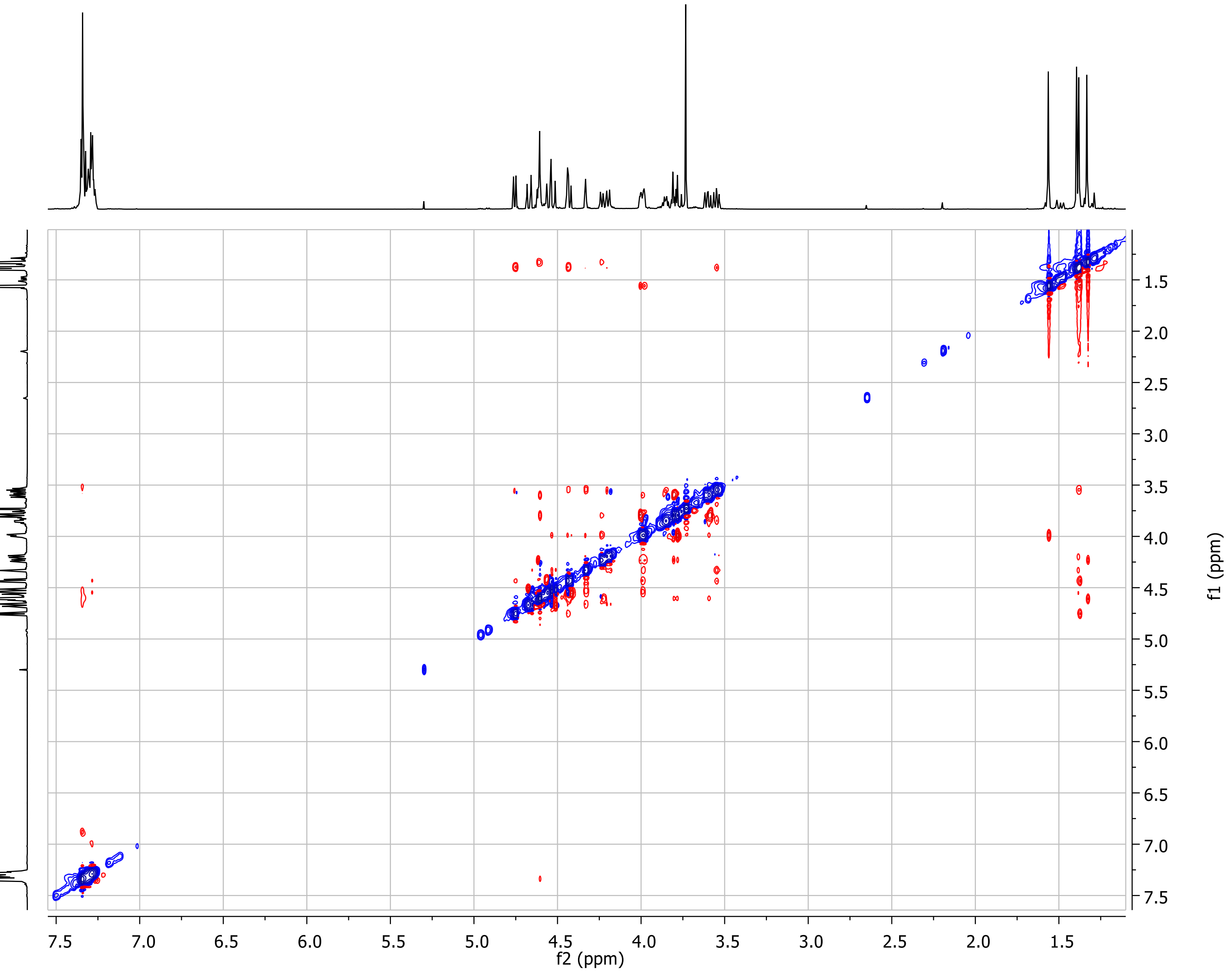

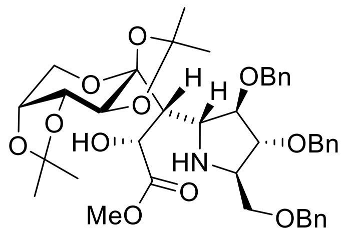
OBn
OBn
OBn
HO
HN
MeO
O
H
H

24

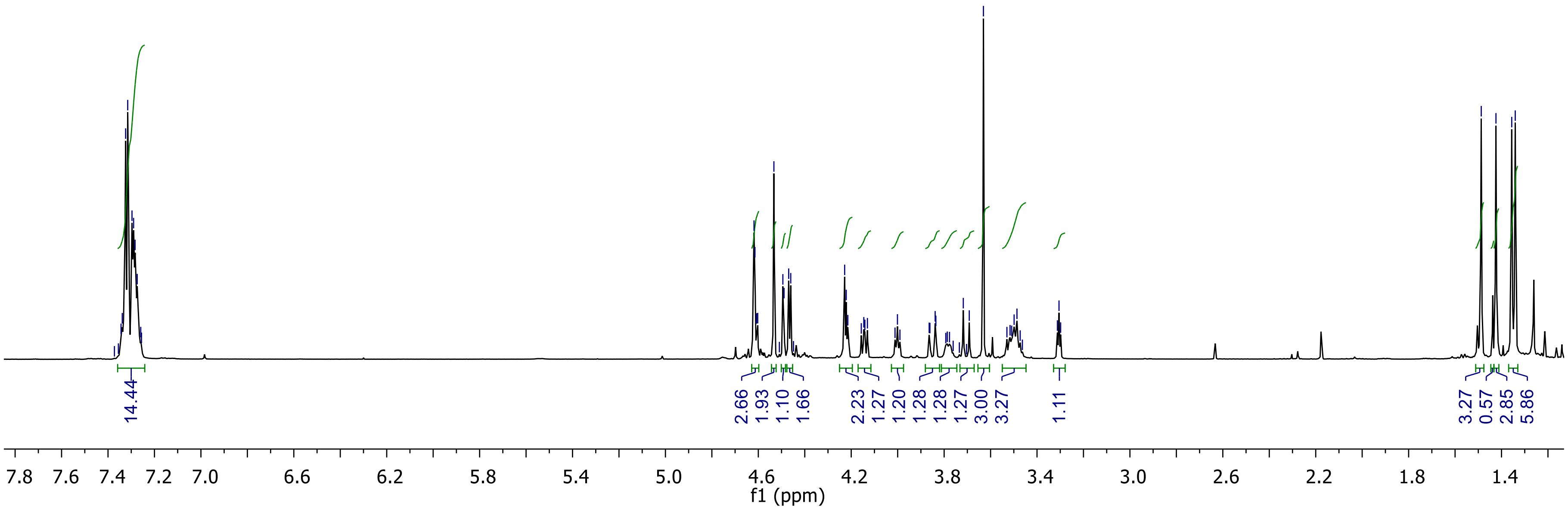
f1 (ppm)
7.8 7.6 7.4 7.2 7.0 6.6 6.2 5.8 5.4 5.0 4.6 4.2 3.8 3.4 3.0 2.6 2.2 1.8 1.4
14.44
2.66 1.93 1.10 1.66
2.23 1.27 1.20 1.28 1.28 1.27 3.00 3.27
1.11
3.27 0.57 2.85 5.86

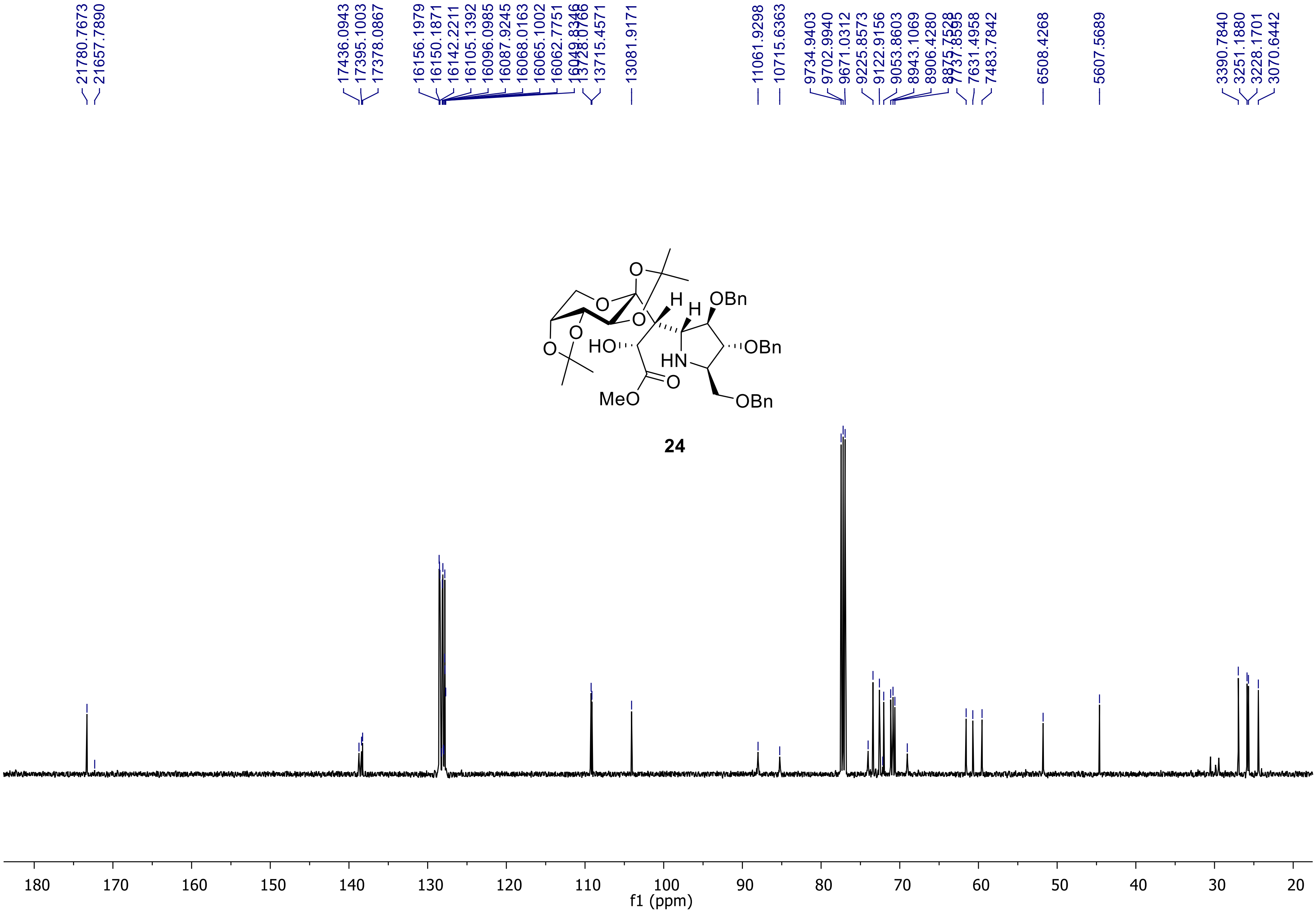
21780.7673
21657.7890
17436.0943
17395.1003
17378.0867
16156.1979
16150.1871
16142.2211
16105.1392
16096.0985
16087.9245
16068.0163
16065.1002
16062.7751
13715.4571
13081.9171
11061.9298
10715.6363
9734.9403
9702.9940
9671.0312
9225.8573
9122.9156
9053.8603
8943.1069
8906.4280
7631.4958
7483.7842
6508.4268
5607.5689
3390.7840
3251.1880
3228.1701
3070.6442
OBn
OBn
OBn
HO
HN
MeO
24
180 170 160 150 140 130 120 110 100 90 80 70 60 50 40 30 20
f1 (ppm)

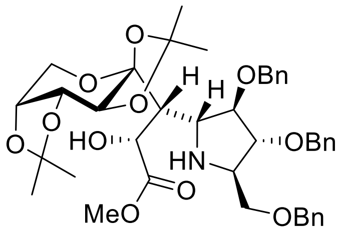

24

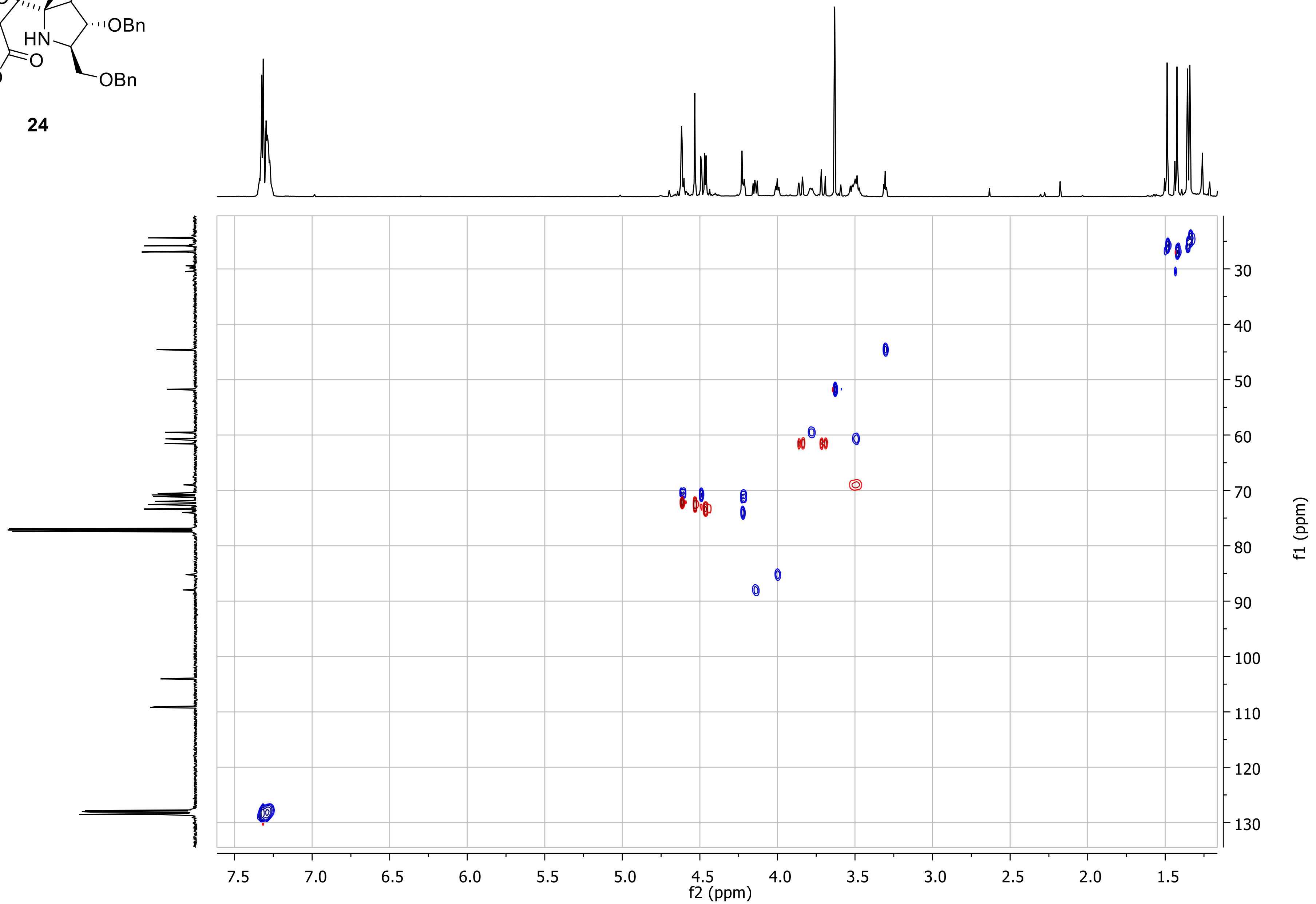

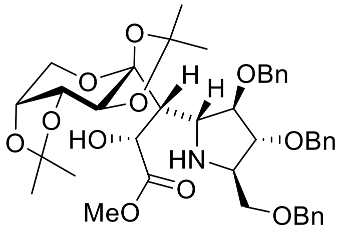

24

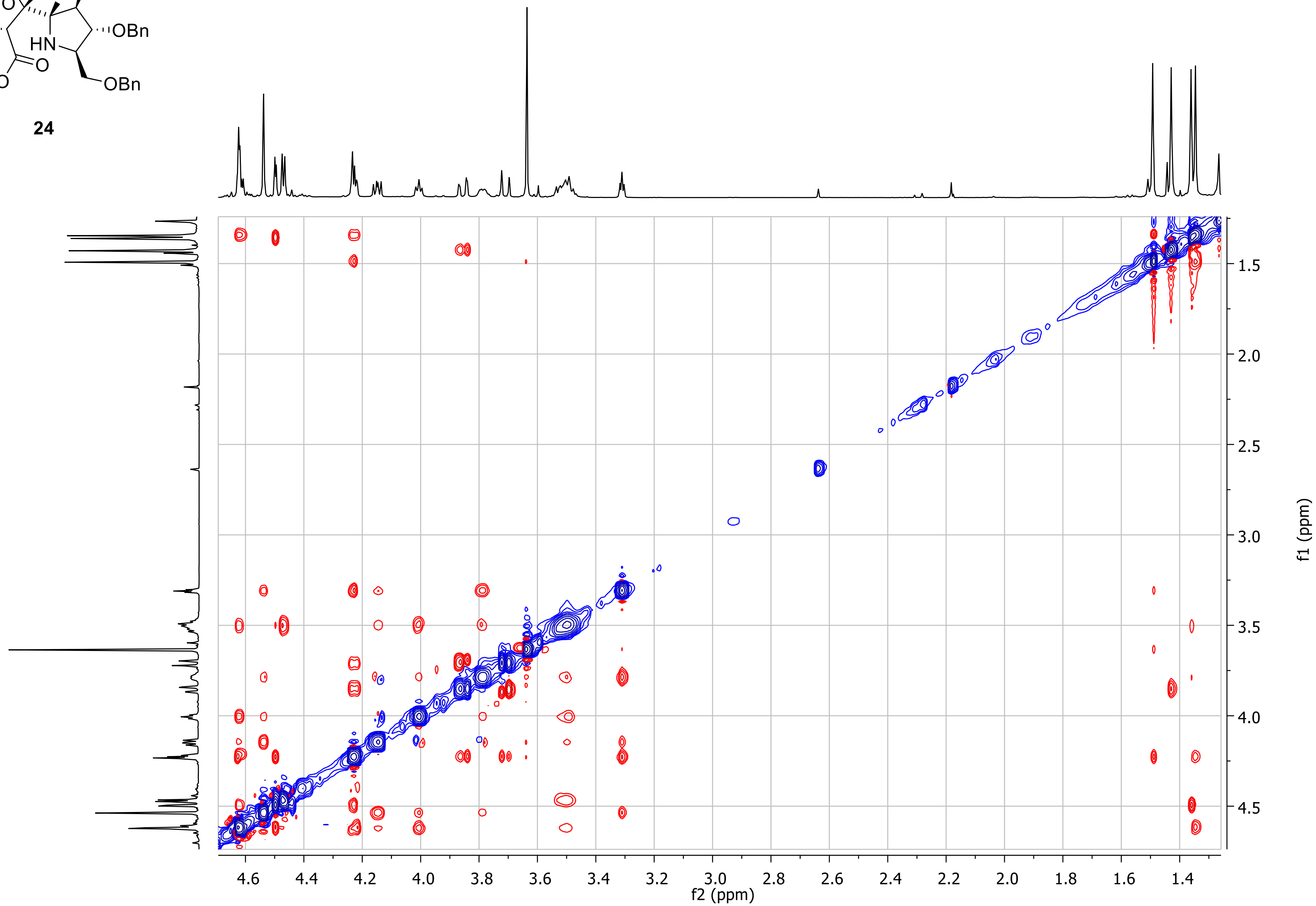

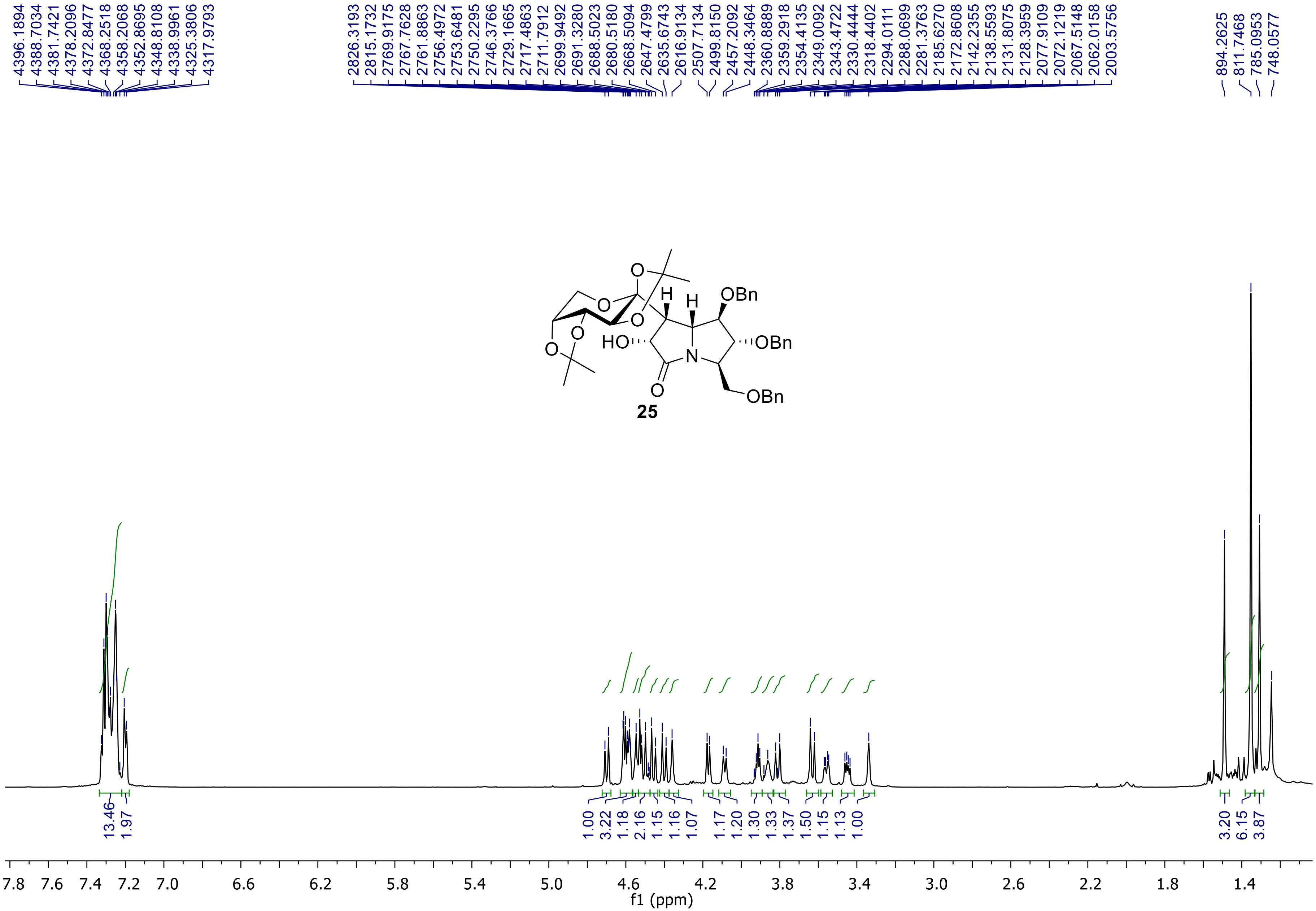
4396.1894
4388.7034
4381.7421
4378.2096
4372.8477
4368.2518
4358.2068
4352.8695
4348.8108
4338.9961
4325.3806
4317.9793
2826.3193
2815.1732
2769.9175
2767.7628
2761.8863
2756.4972
2753.6481
2750.2295
2746.3766
2729.1665
2717.4863
2711.7912
2699.9492
2691.3280
2688.5023
2680.5180
2668.5094
2647.4799
2635.6743
2616.9134
2507.7134
2499.8150
2457.2092
2448.3464
2360.8889
2359.2918
2354.4135
2349.0092
2343.4722
2330.4444
2318.4402
2294.0111
2288.0699
2281.3763
2185.6270
2172.8608
2142.2355
2138.5593
2131.8075
2128.3959
2077.9109
2072.1219
2067.5148
2062.0158
2003.5756
894.2625
811.7468
785.0953
748.0577
HO
OBn
OBn
OBn
N
H
H
O
O
O
O
O
O
25
13.46
1.97
1.00
3.22
1.18
2.16
1.15
1.16
1.07
1.17
1.20
1.30
1.33
1.37
1.50
1.15
1.13
1.00
3.20
6.15
3.87
7.8
7.6
7.4
7.2
7.0
6.6
6.2
5.8
5.4
5.0
4.6
4.2
3.8
3.4
3.0
2.6
2.2
1.8
1.4
f1 (ppm)

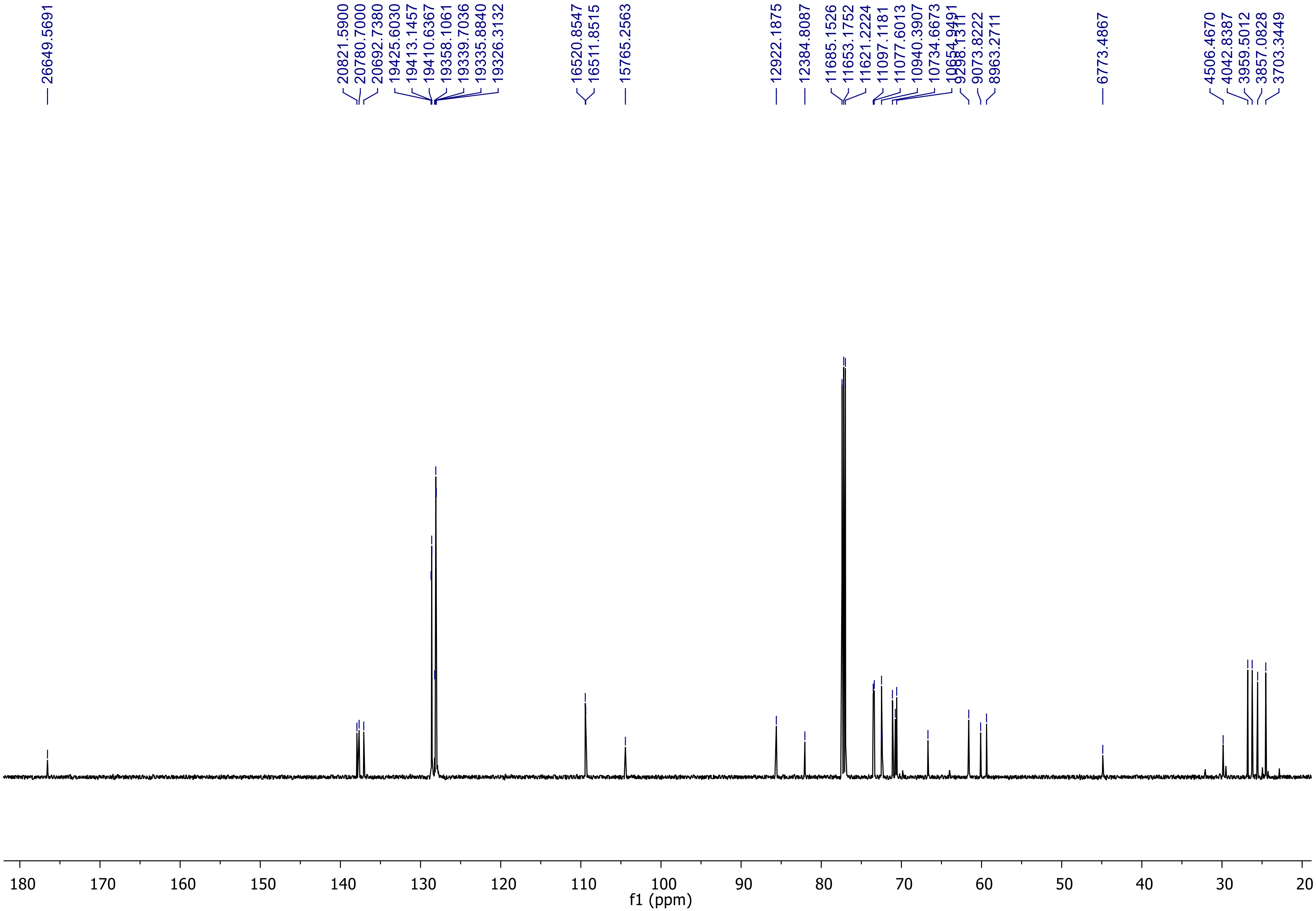
26649.5691
20821.5900
20780.7000
20692.7380
19425.6030
19413.1457
19410.6367
19358.1061
19339.7036
19335.8840
19326.3132
16520.8547
16511.8515
15765.2563
12922.1875
12384.8087
11685.1526
11653.1752
11621.2224
11097.1181
11077.6013
10940.3907
10734.6673
9073.8222
8963.2711
6773.4867
4506.4670
4042.8387
3959.5012
3857.0828
3703.3449
180
170
160
150
140
130
120
110
100
90
80
70
60
50
40
30
20
f1 (ppm)

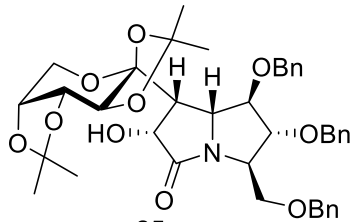

25

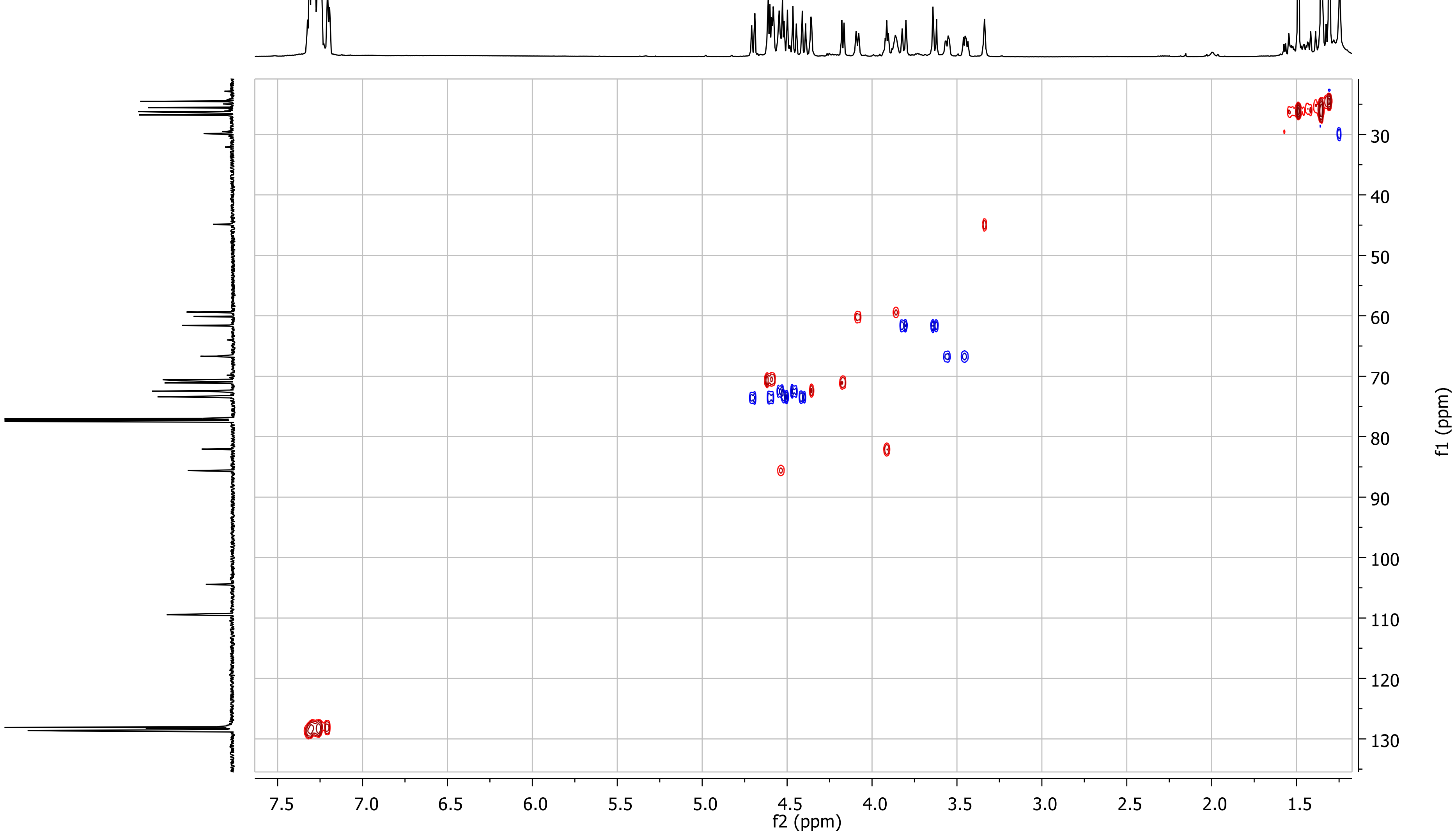

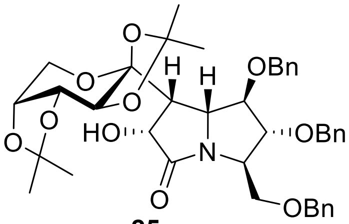
OBn
HO
OBn
N
OBn

25

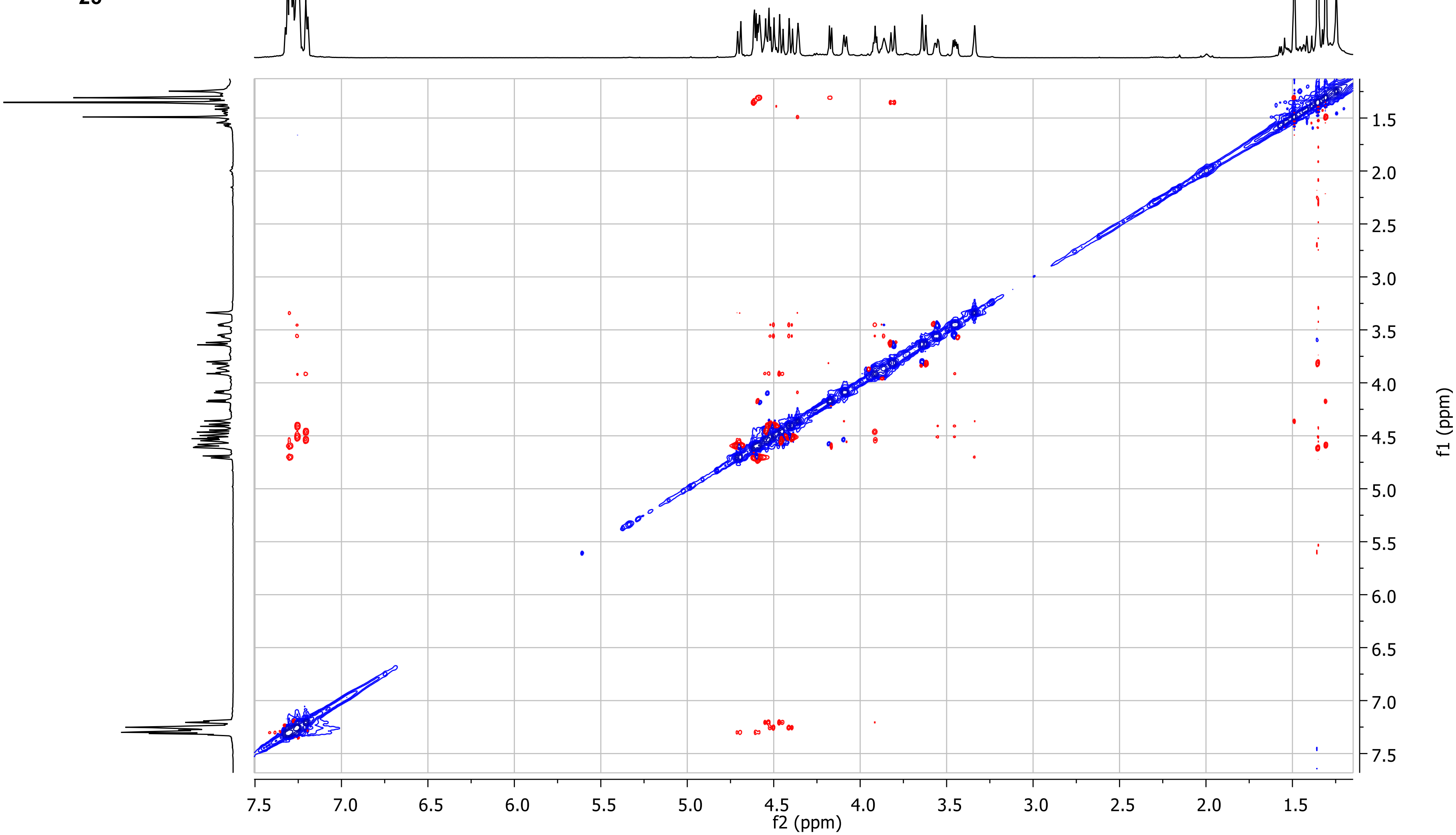
f2 (ppm)
f1 (ppm)
7.5 7.0 6.5 6.0 5.5 5.0 4.5 4.0 3.5 3.0 2.5 2.0 1.5
1.5 2.0 2.5 3.0 3.5 4.0 4.5 5.0 5.5 6.0 6.5 7.0 7.5

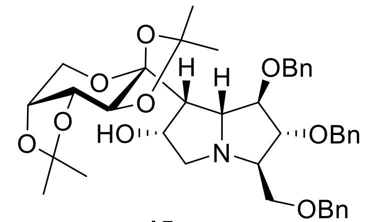

15

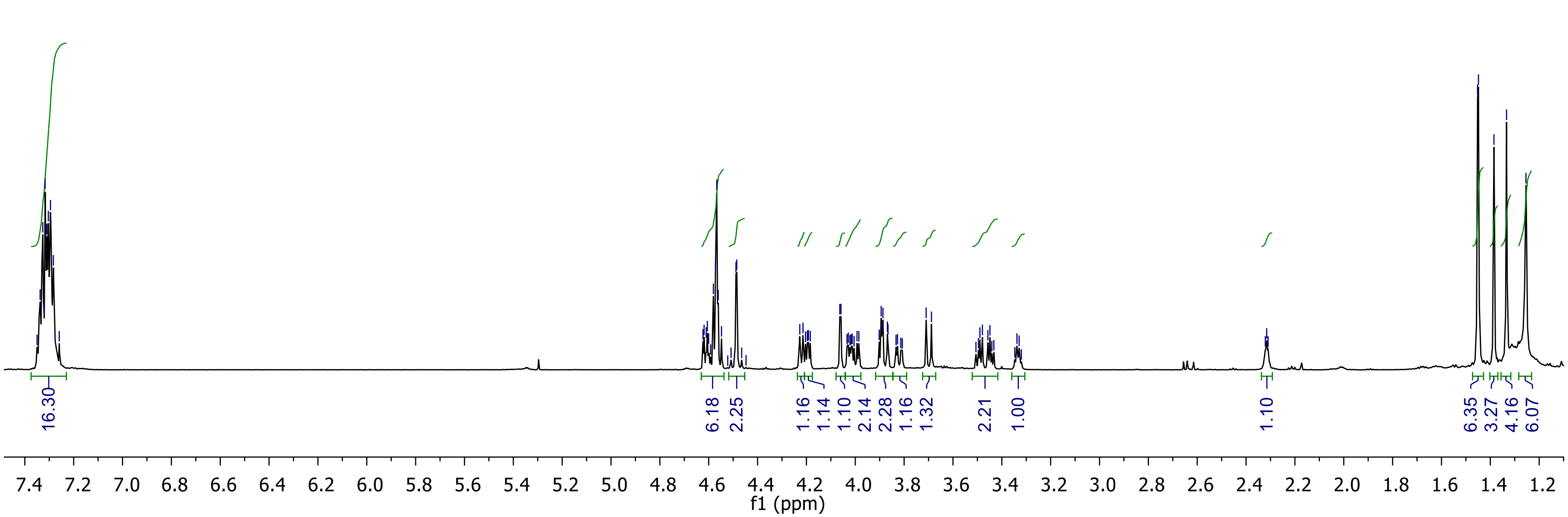

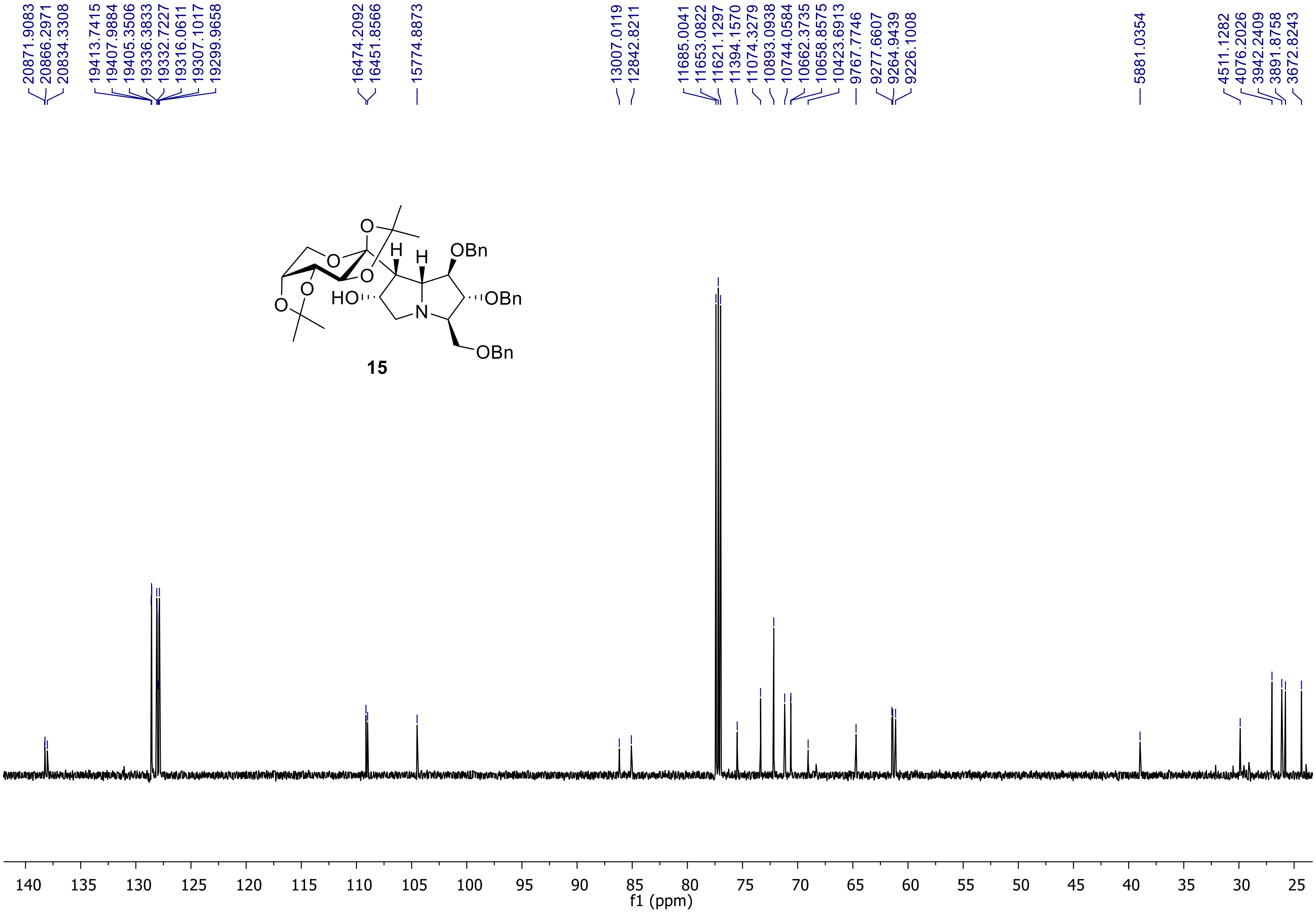
20871.9083
20866.2971
20834.3308
19413.7415
19407.9884
19405.3506
19336.3833
19332.7227
19316.0611
19307.1017
19299.9658
16474.2092
16451.8566
15774.8873
13007.0119
12842.8211
11685.0041
11653.0822
11621.1297
11394.1570
11074.3279
10893.0938
10744.0584
10662.3735
10658.8575
10423.6913
9767.7746
9277.6607
9264.9439
9226.1008
5881.0354
4511.1282
4076.2026
3942.2409
3891.8758
3672.8243
H
H
OBn
HO
OBn
N
OBn
15
140
135
130
125
120
115
110
105
100
95
90
85
80
75
70
65
60
55
50
45
40
35
30
25
f1 (ppm)

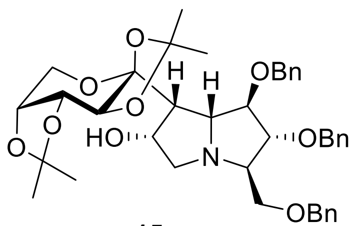

O
O
O
O
O
H
H
OBn
HO
OBn
N
OBn
15

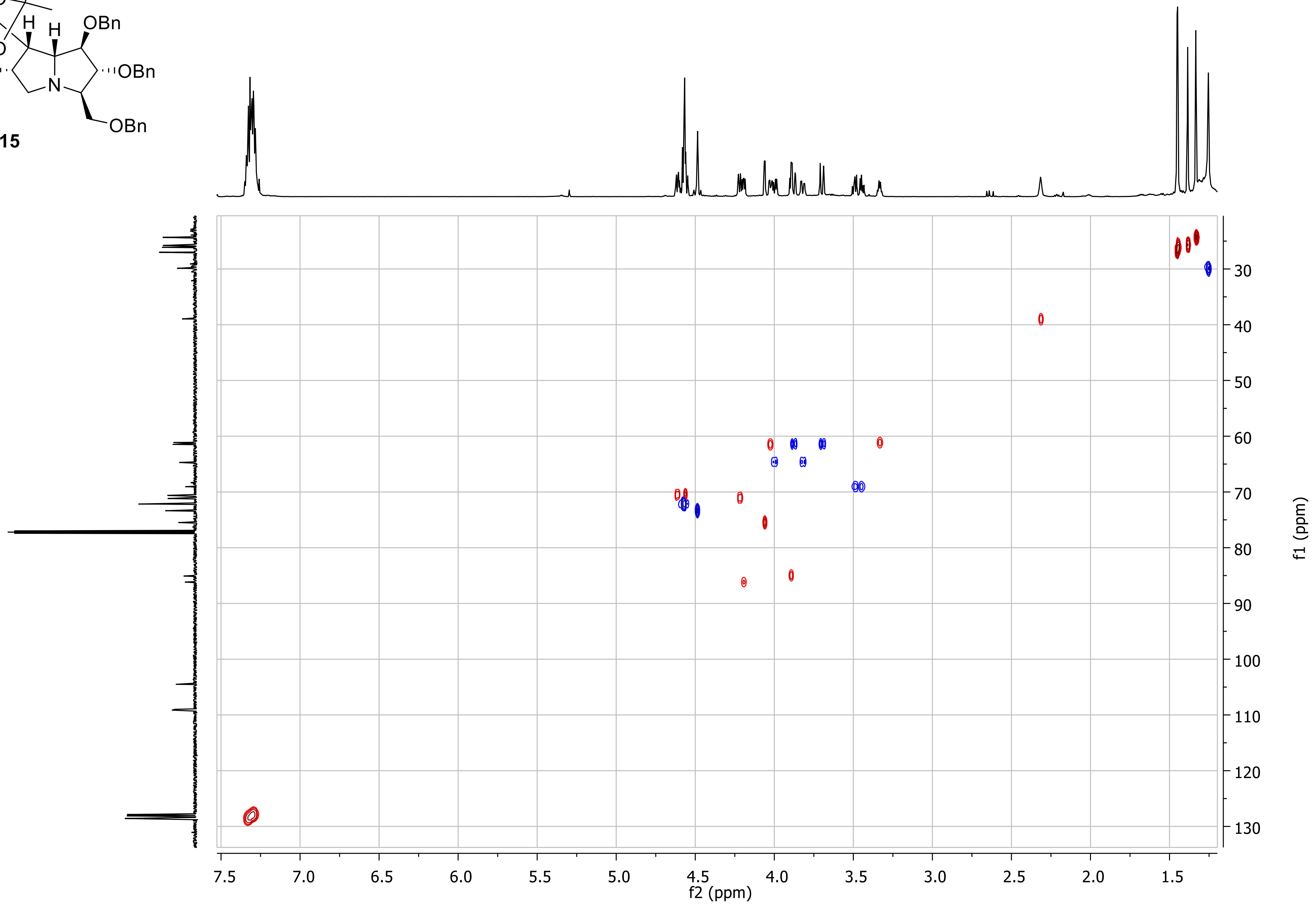

7.5
7.0
6.5
6.0
5.5
5.0
4.5
4.0
3.5
3.0
2.5
2.0
1.5
f2 (ppm)
30
40
50
60
70
80
90
100
110
120
130
f1 (ppm)

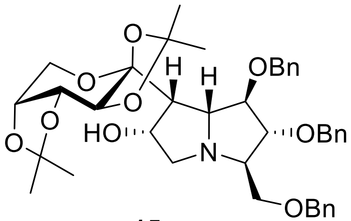

15

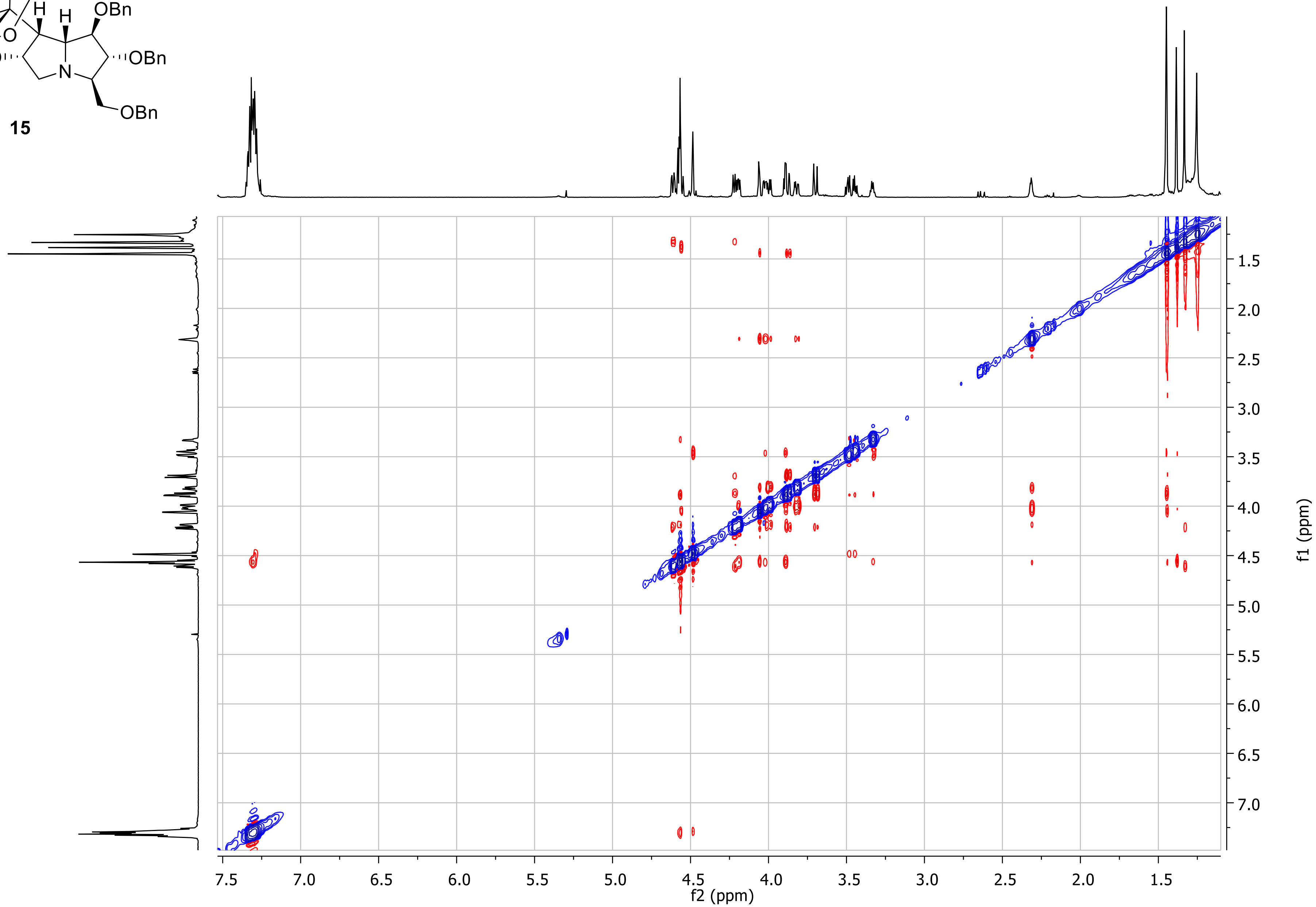

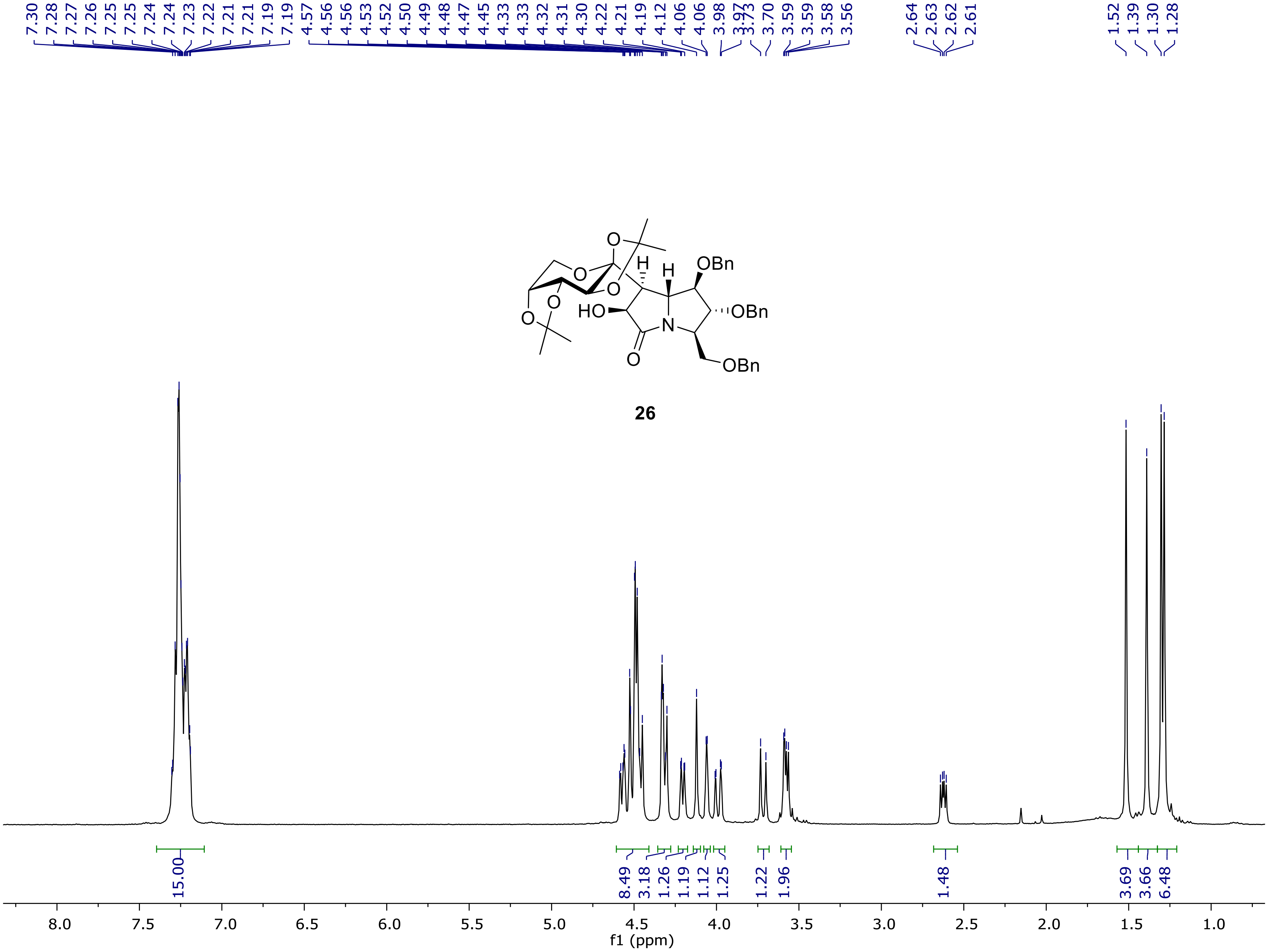
7.30 7.28 7.27 7.26 7.25 7.25 7.24 7.24 7.23 7.22 7.21 7.21 7.19 7.19 4.57 4.56 4.56 4.53 4.52 4.50 4.49 4.48 4.47 4.45 4.33 4.33 4.32 4.31 4.30 4.22 4.21 4.19 4.12 4.06 4.06 3.98 3.97 3.73 3.70 3.59 3.59 3.58 3.56 2.64 2.63 2.62 2.61 1.52 1.39 1.30 1.28
OBn
OBn
OBn
HO
N
O
H
H
26
15.00
8.49 3.18 1.26 1.19 1.12 1.25 1.22 1.96 1.48 3.69 3.66 6.48
8.0 7.5 7.0 6.5 6.0 5.5 5.0 4.5 4.0 3.5 3.0 2.5 2.0 1.5 1.0
f1 (ppm)

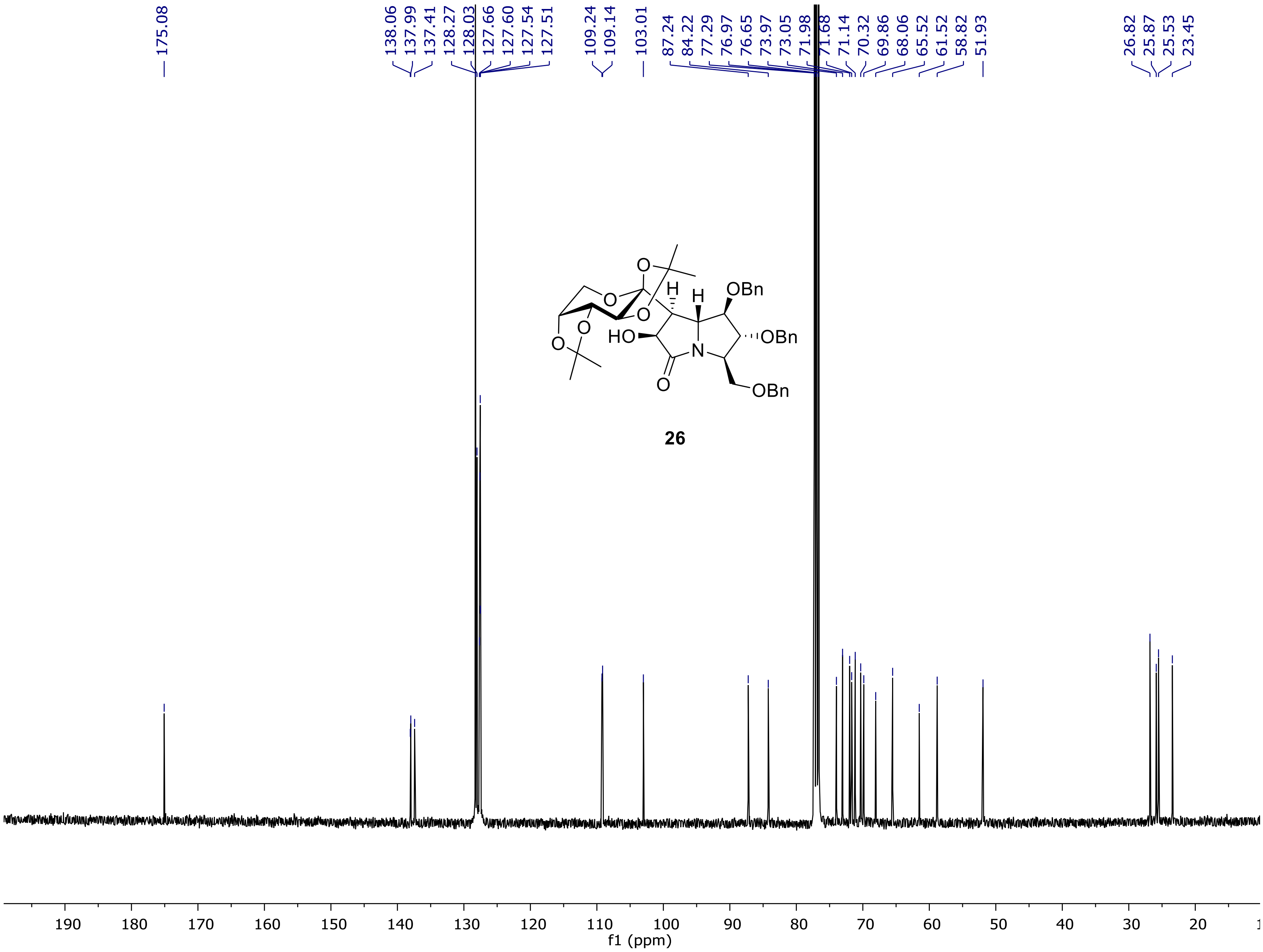
175.08
138.06
137.99
137.41
128.27
128.03
127.66
127.60
127.54
127.51
109.24
109.14
103.01
87.24
84.22
77.29
76.97
76.65
73.97
73.05
71.98
71.68
71.14
70.32
69.86
68.06
65.52
61.52
58.82
51.93
26.82
25.87
25.53
23.45
O
O
O
O
O
H
H
OBn
HO
N
OBn
O
OBn
f1 (ppm)

26

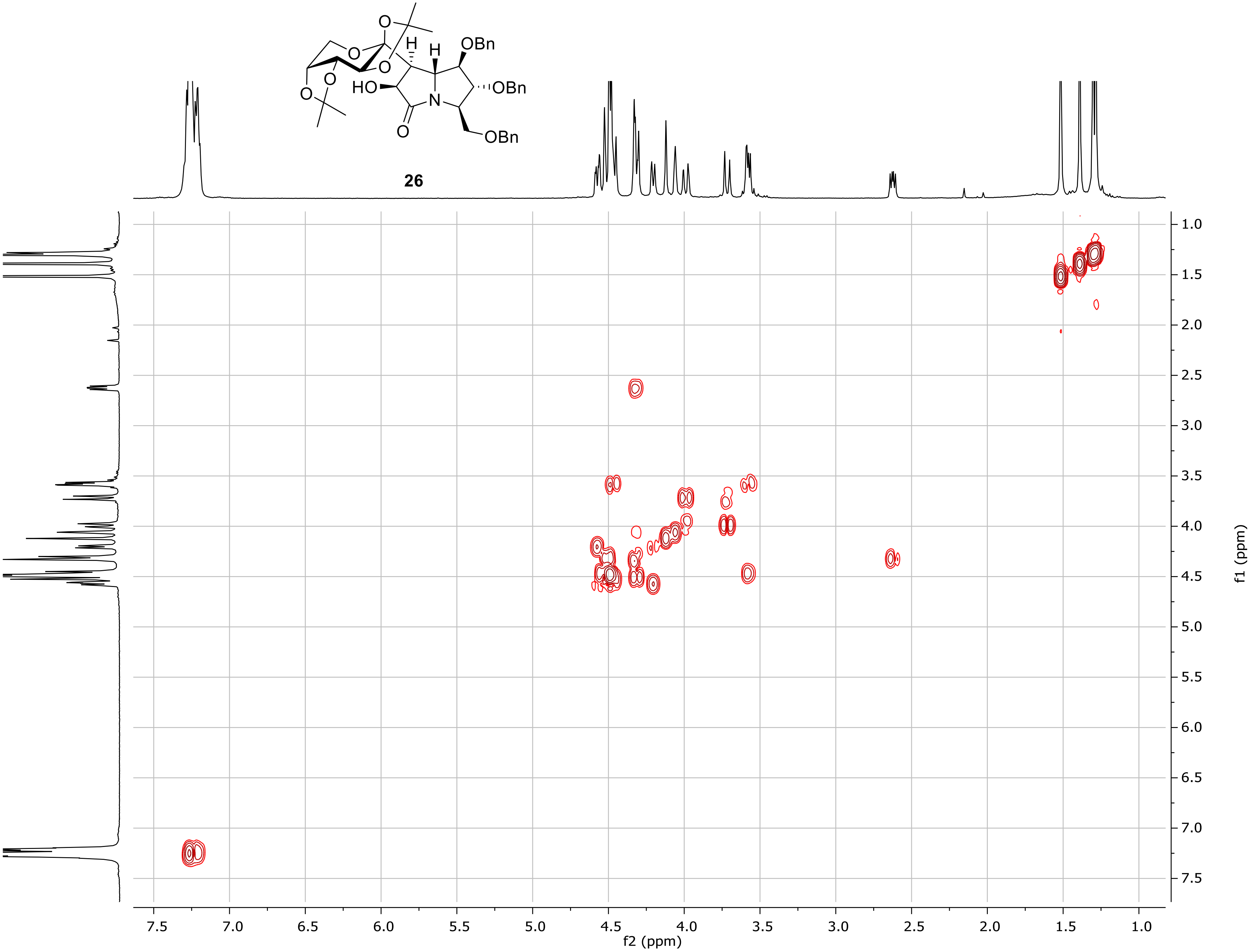
OBn
OBn
OBn
HO
O
N
H
H
26
f2 (ppm)
f1 (ppm)

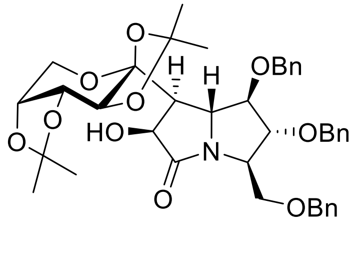

26

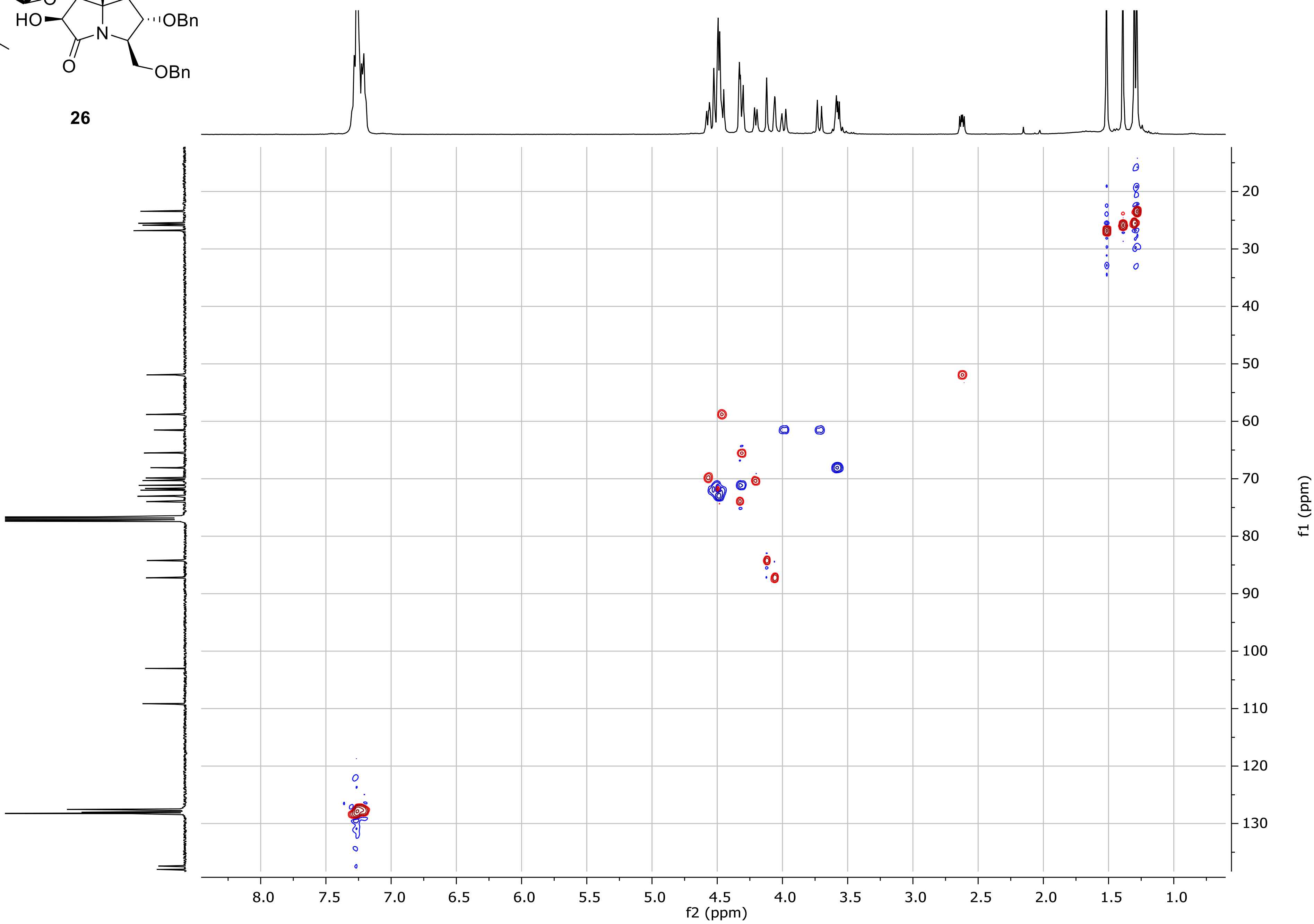

f2 (ppm)
f1 (ppm)
8.0
7.5
7.0
6.5
6.0
5.5
5.0
4.5
4.0
3.5
3.0
2.5
2.0
1.5
1.0
20
30
40
50
60
70
80
90
100
110
120
130

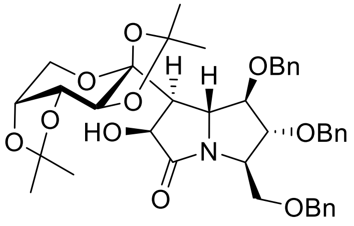

26

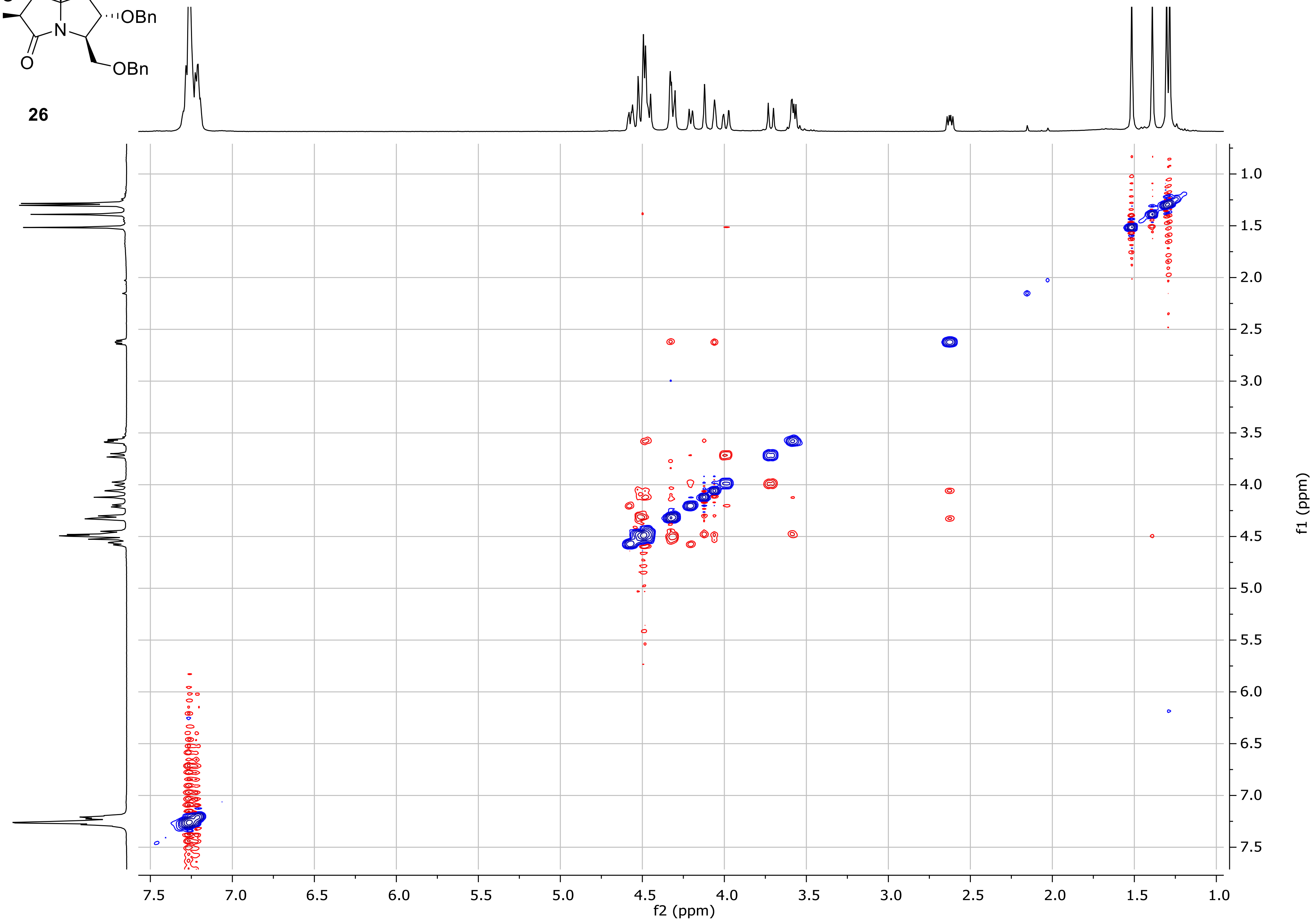

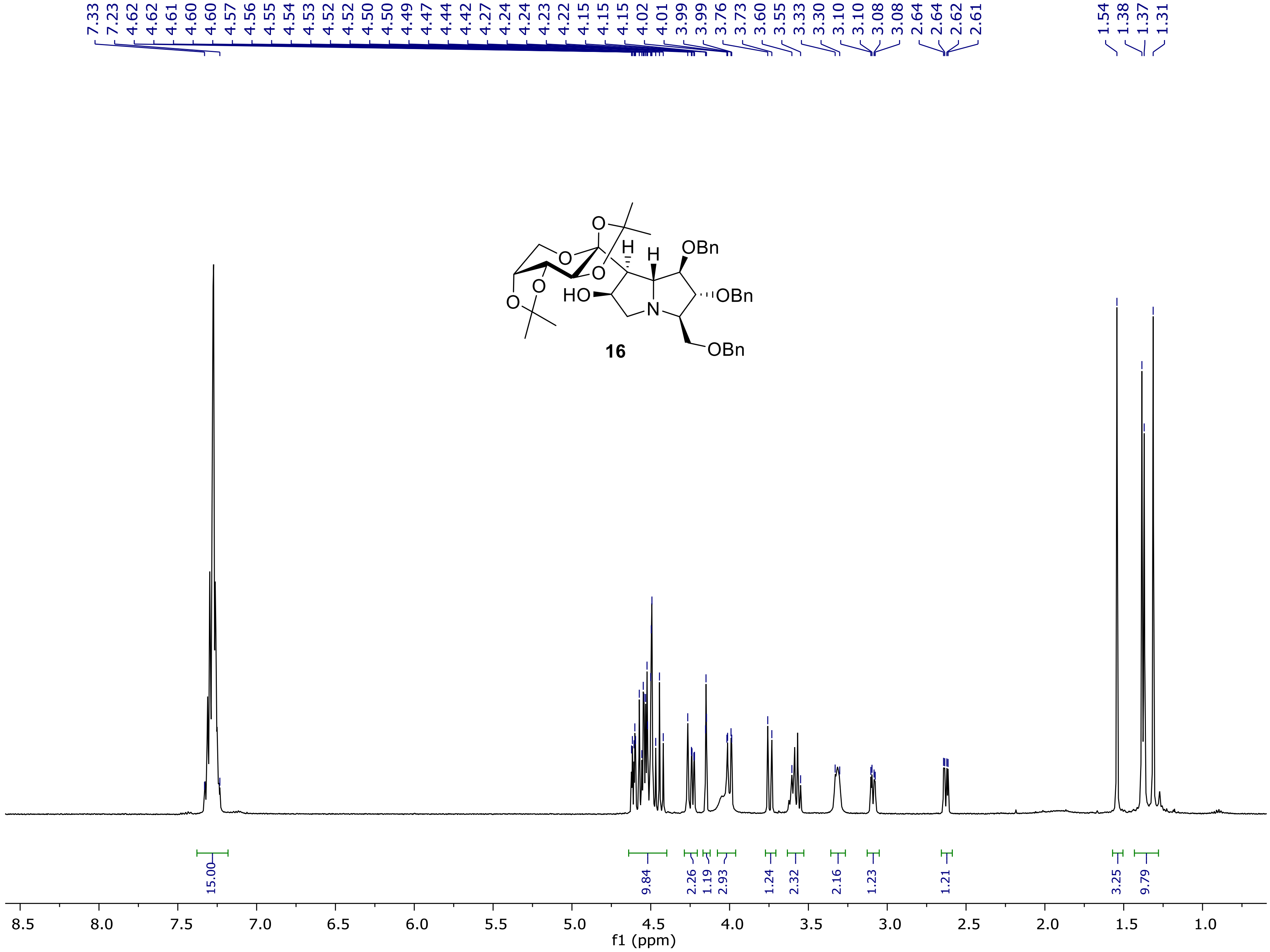
7.33 7.23 4.62 4.62 4.61 4.60 4.60 4.57 4.56 4.55 4.54 4.53 4.52 4.52 4.50 4.50 4.49 4.47 4.44 4.42 4.27 4.24 4.24 4.23 4.22 4.15 4.15 4.15 4.02 4.01 3.99 3.99 3.76 3.73 3.60 3.55 3.33 3.30 3.10 3.10 3.08 3.08 2.64 2.64 2.62 2.61 1.54 1.38 1.37 1.31
OBn
OBn
OBn
HO
N
H
H
O
O
O
O
O
16
15.00 9.84 2.26 1.19 2.93 1.24 2.32 2.16 1.23 1.21 3.25 9.79
8.5 8.0 7.5 7.0 6.5 6.0 5.5 5.0 4.5 4.0 3.5 3.0 2.5 2.0 1.5 1.0
f1 (ppm)

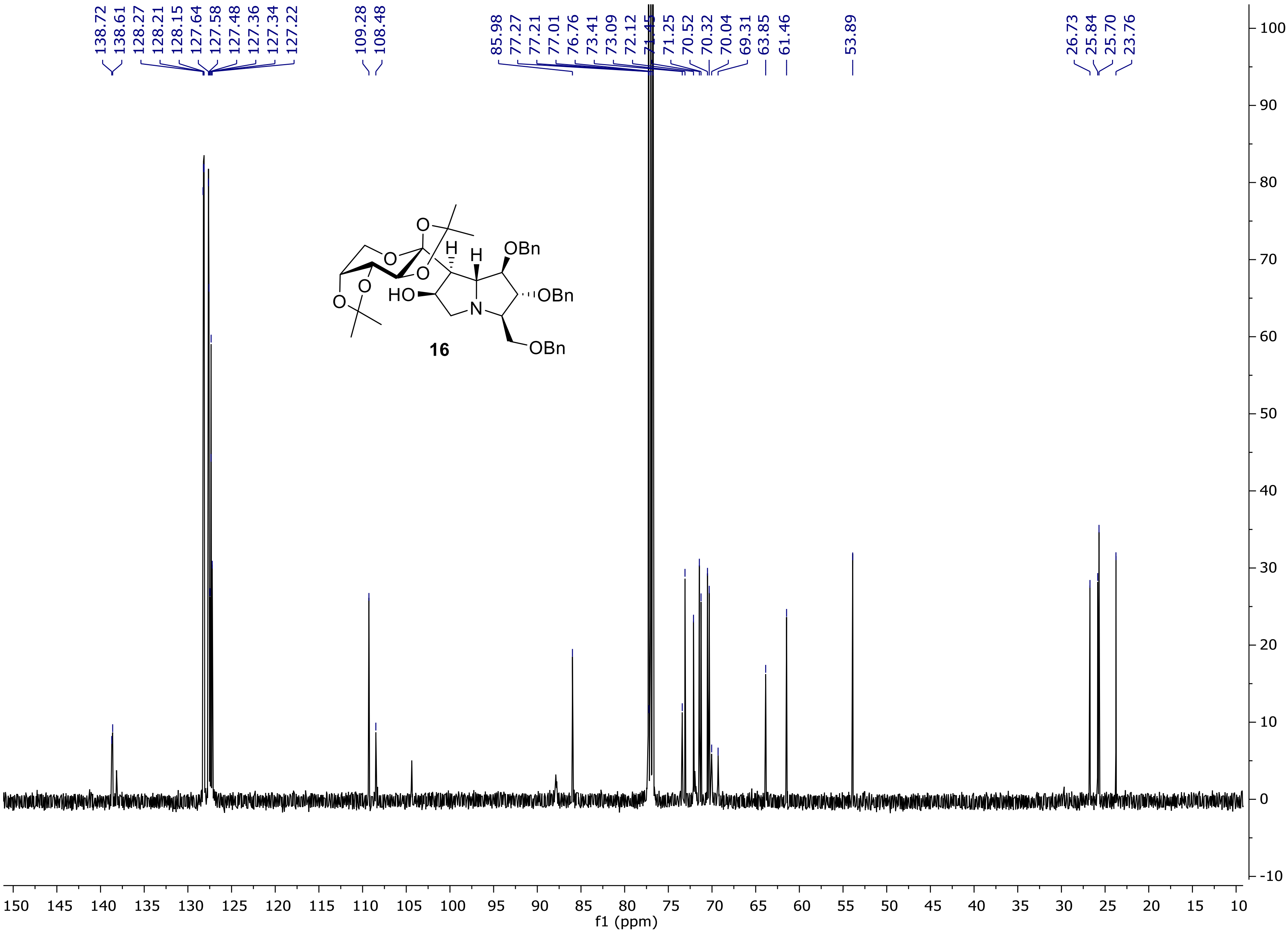
138.72
138.61
128.27
128.21
128.15
127.64
127.58
127.48
127.36
127.34
127.22
109.28
108.48
85.98
77.27
77.21
77.01
76.76
73.41
73.09
72.12
71.45
71.25
70.52
70.32
70.04
69.31
63.85
61.46
53.89
26.73
25.84
25.70
23.76
OBn
OBn
OBn
HO
H
H
N
O
16
f1 (ppm)

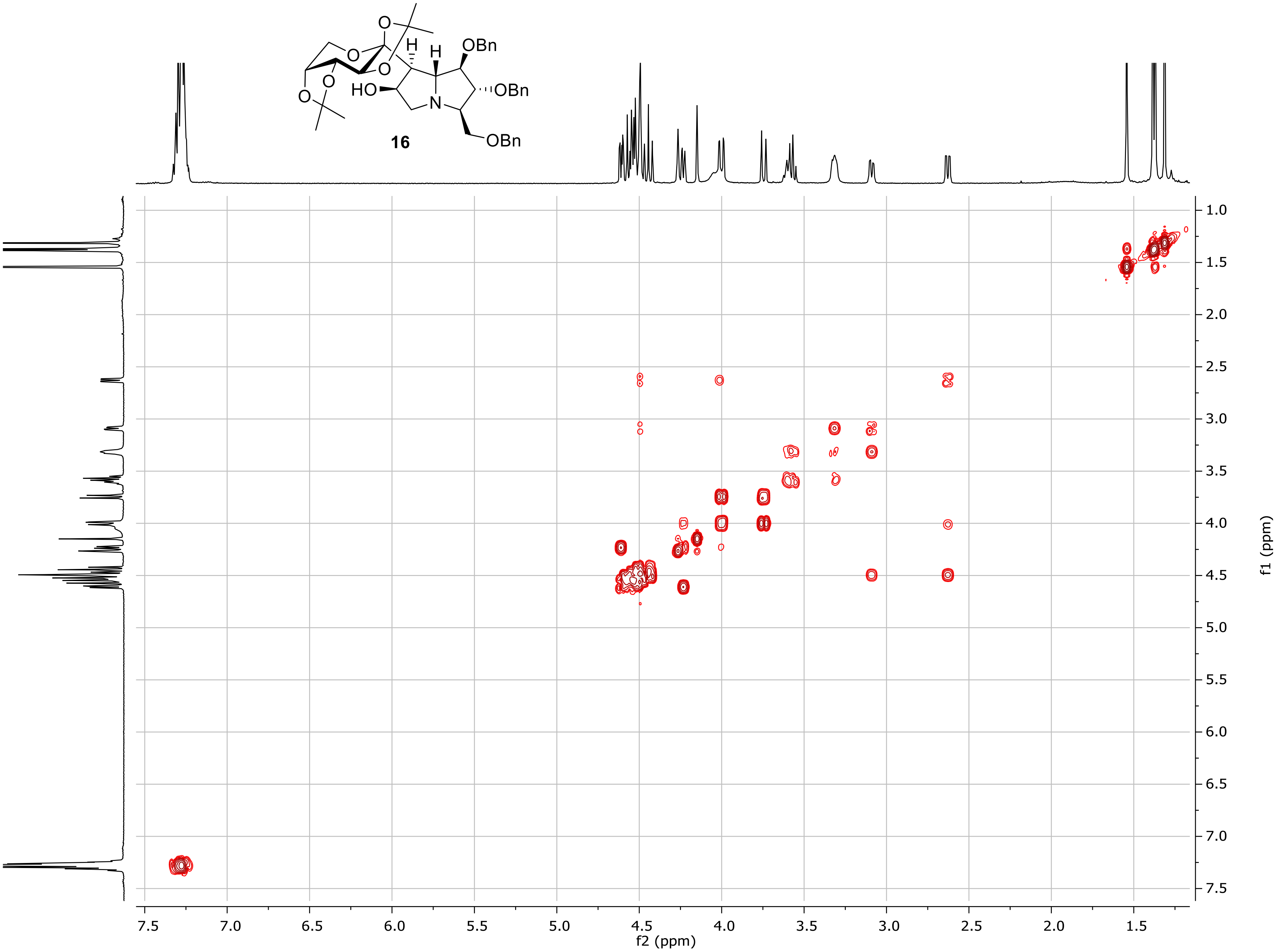

OBn
OBn
HO
N
OBn
H
H
O
O
O
O
O
16
f2 (ppm)
f1 (ppm)

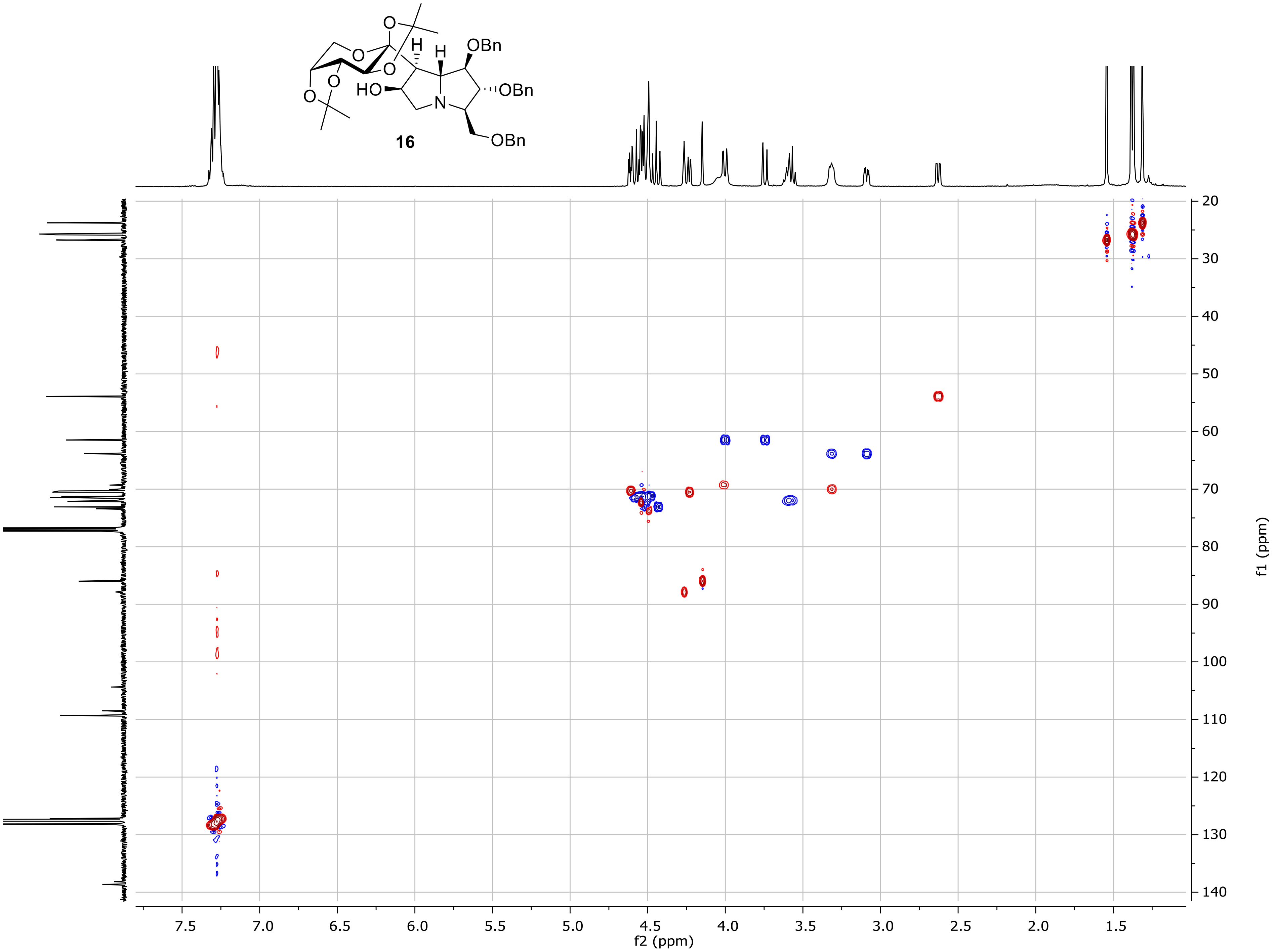
O
O
O
H
H
OBn
O
HO
OBn
O
N
16
OBn
7.5
7.0
6.5
6.0
5.5
5.0
4.5
4.0
3.5
3.0
2.5
2.0
1.5
f2 (ppm)
20
30
40
50
60
70
80
90
100
110
120
130
140
f1 (ppm)

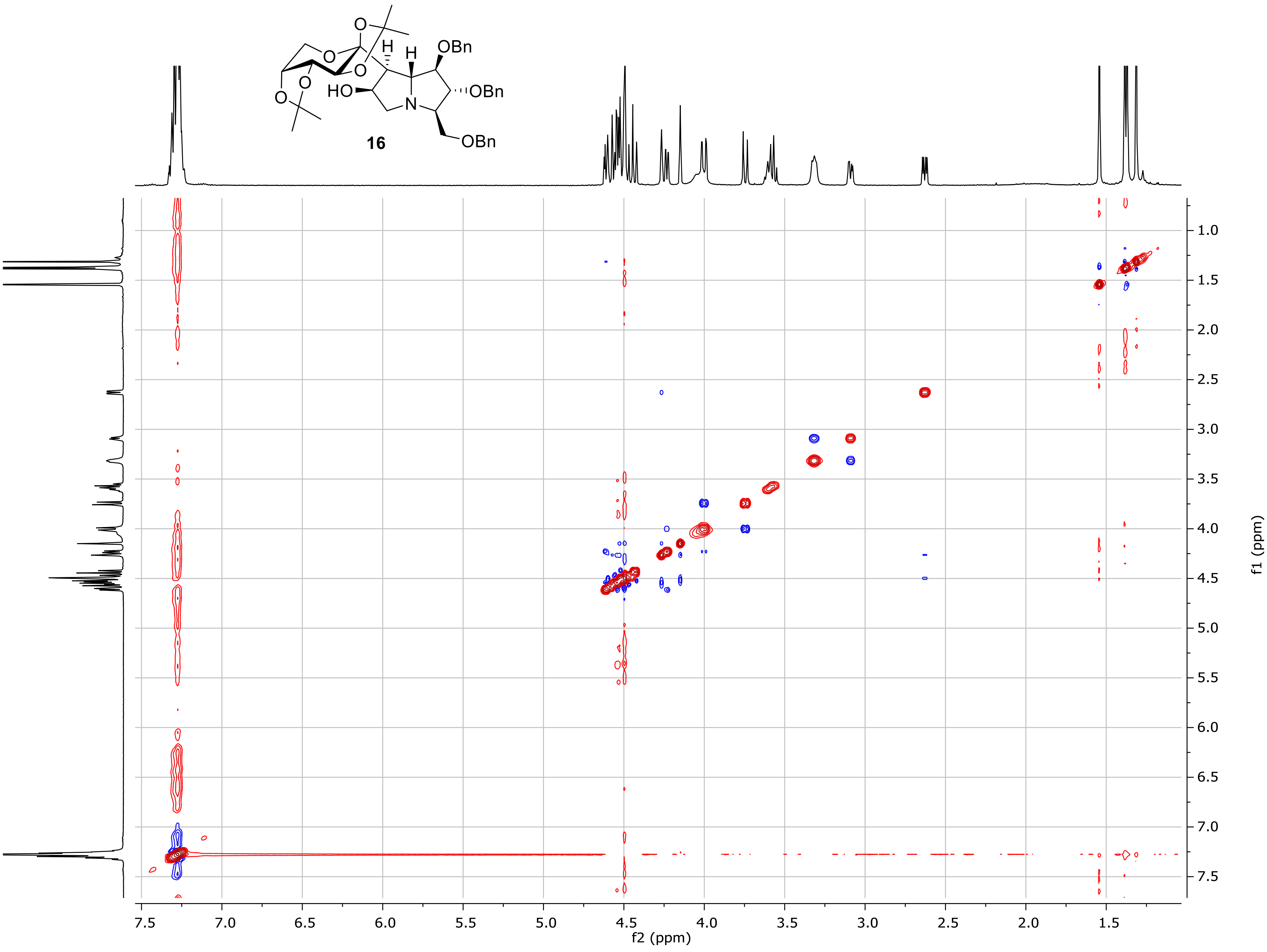
16
f2 (ppm)
f1 (ppm)

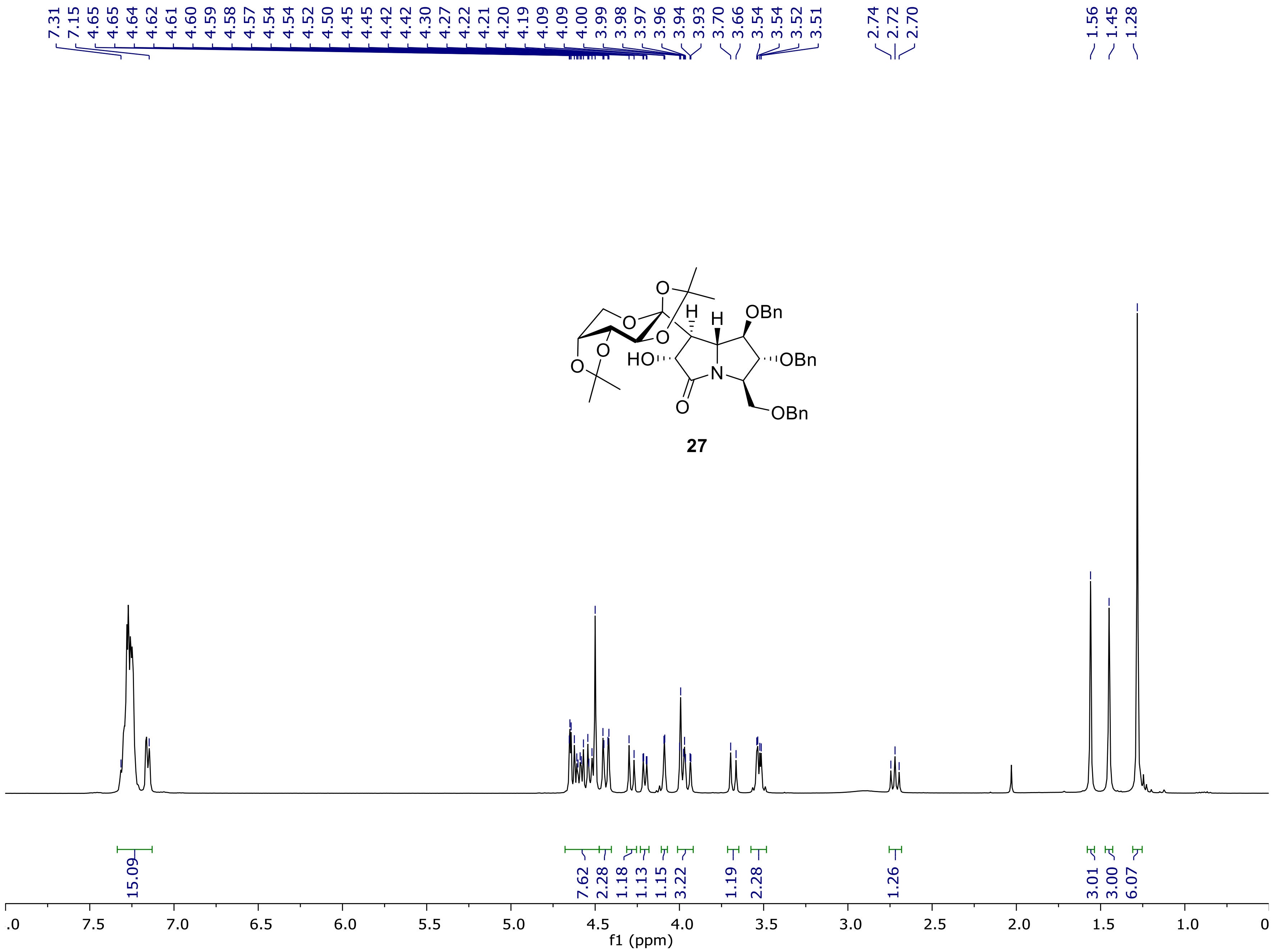
7.31 7.15 4.65 4.65 4.64 4.62 4.61 4.60 4.59 4.58 4.57 4.54 4.54 4.52 4.50 4.45 4.45 4.42 4.42 4.30 4.27 4.22 4.21 4.20 4.19 4.09 4.09 4.00 3.99 3.98 3.97 3.96 3.94 3.93 3.70 3.66 3.54 3.54 3.52 3.51
2.74 2.72 2.70
1.56 1.45 1.28
OBn
HO
N
OBn
O
OBn
27
15.09
7.62 2.28 1.18 1.13 1.15 3.22
1.19 2.28
1.26
3.01 3.00 6.07
f1 (ppm)

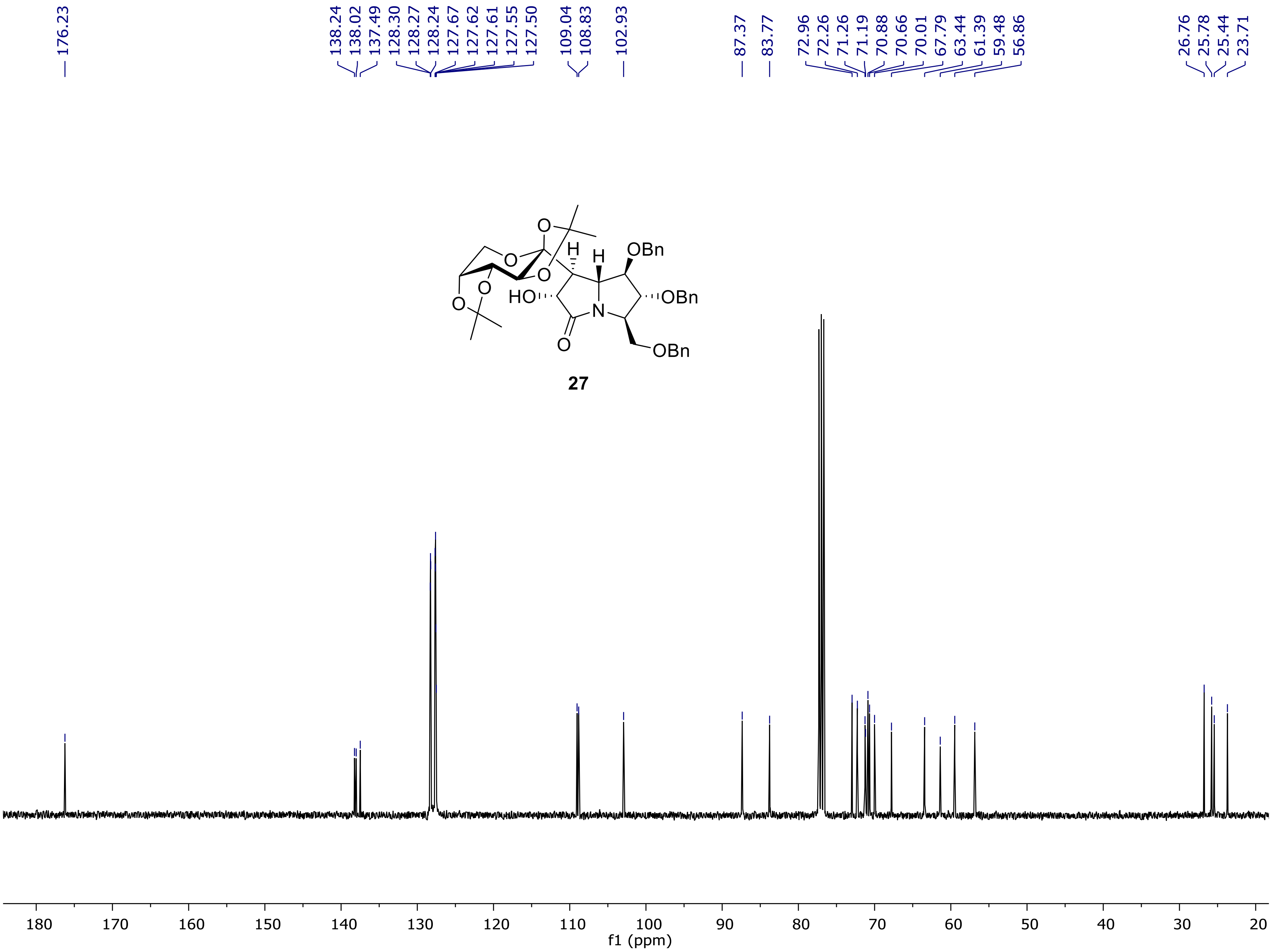
176.23
138.24
138.02
137.49
128.30
128.27
128.24
127.67
127.62
127.61
127.55
127.50
109.04
108.83
102.93
87.37
83.77
72.96
72.26
71.26
71.19
70.88
70.66
70.01
67.79
63.44
61.39
59.48
56.86
26.76
25.78
25.44
23.71
OBn
HO
OBn
OBn
N
27
180
170
160
150
140
130
120
110
100
90
80
70
60
50
40
30
20
f1 (ppm)

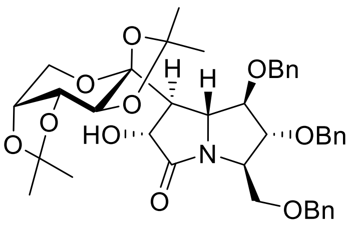

27

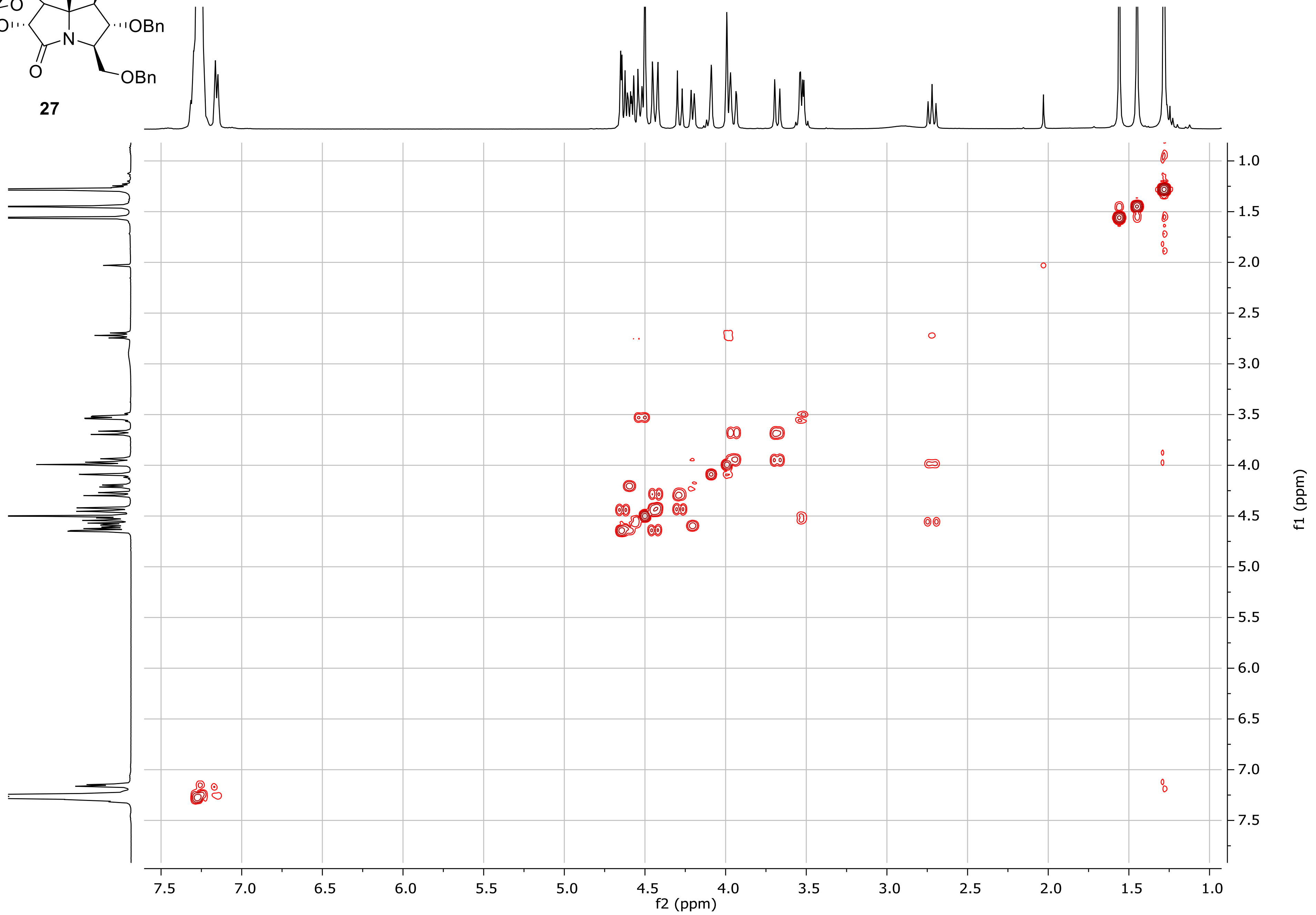

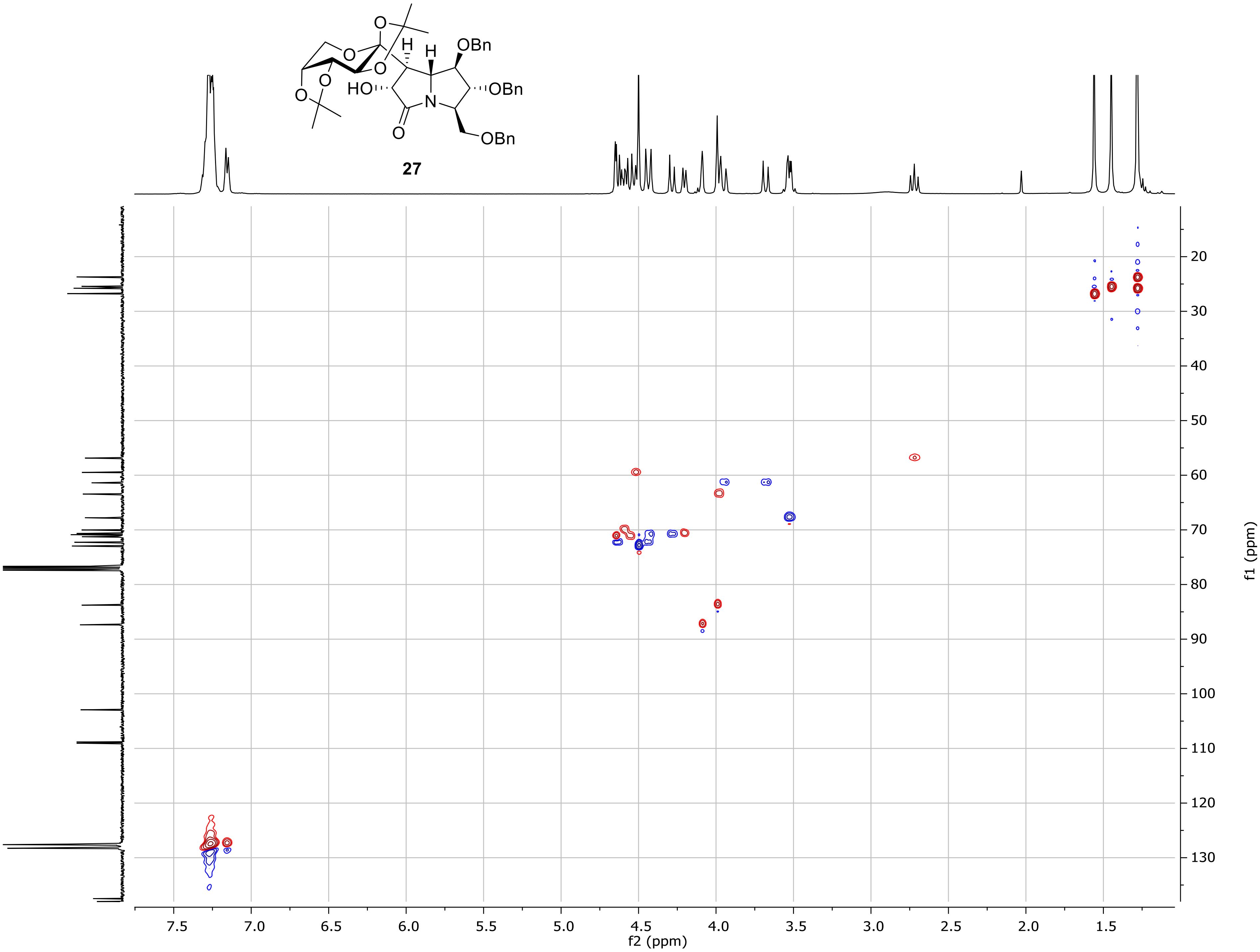
O
O
O
O
O
H
H
OBn
HO
OBn
N
O
OBn
27
f2 (ppm)
f1 (ppm)

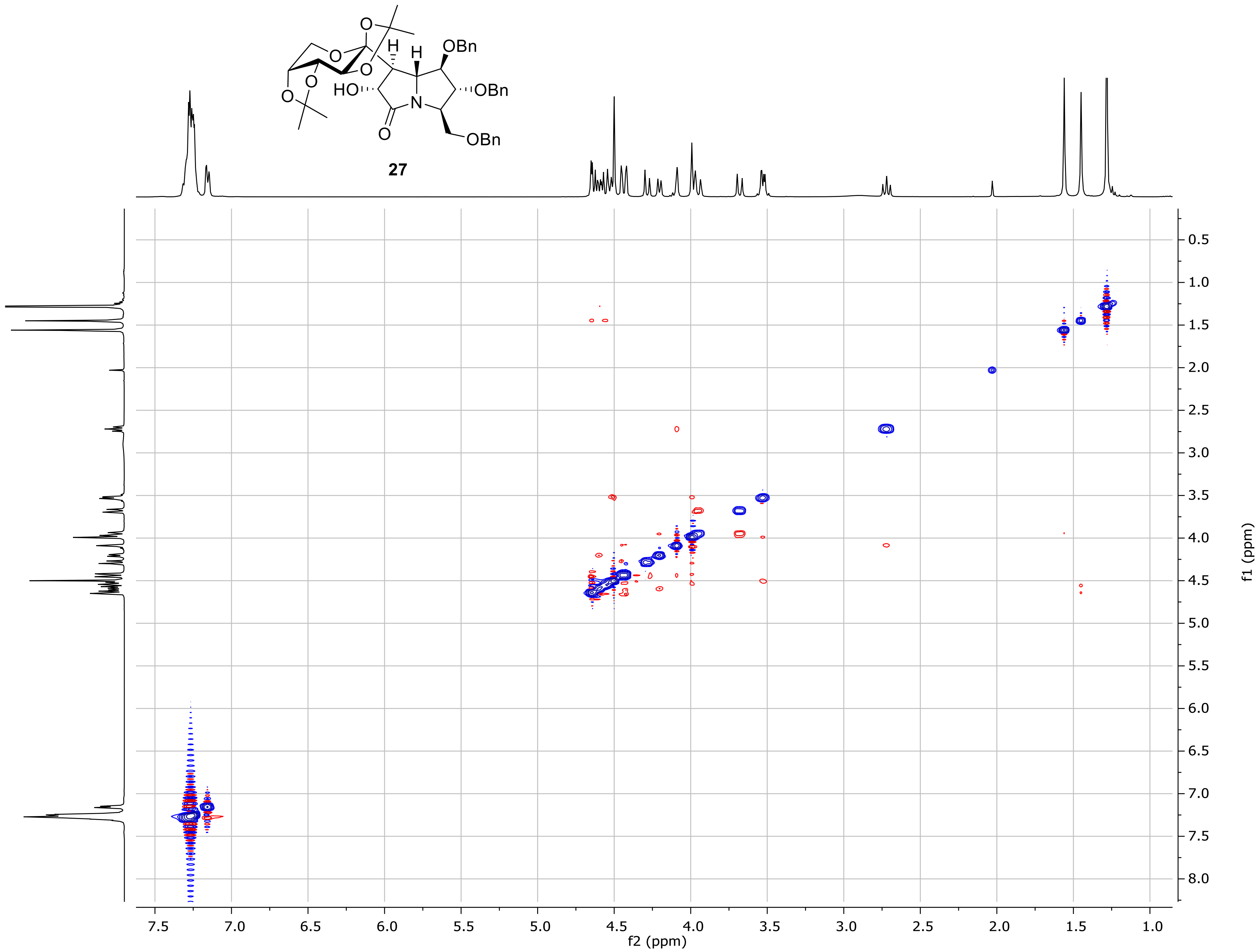

27

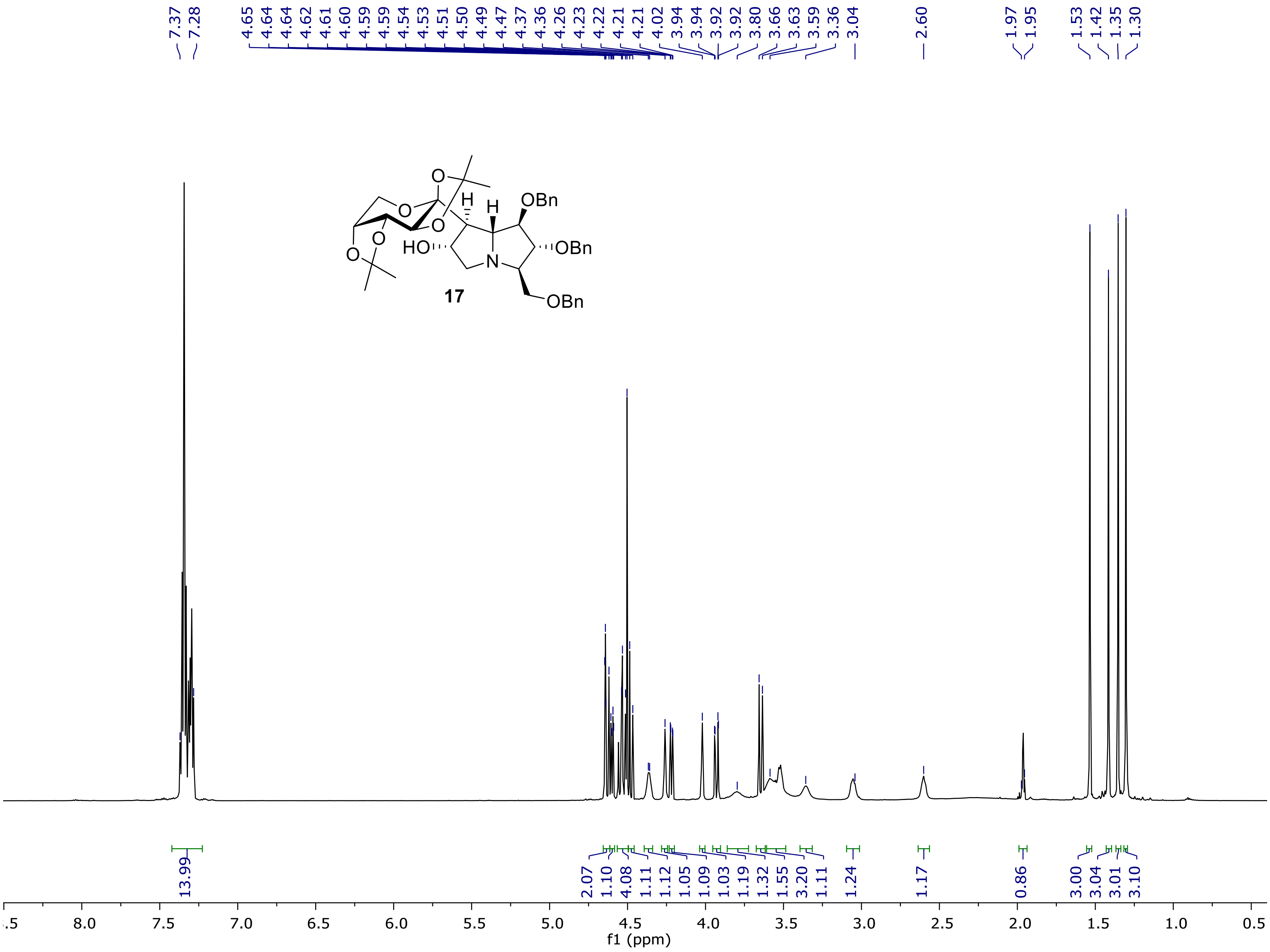

7.37
7.28
4.65
4.64
4.64
4.62
4.61
4.60
4.59
4.59
4.54
4.53
4.51
4.50
4.49
4.47
4.37
4.36
4.26
4.23
4.22
4.21
4.21
4.21
4.02
3.94
3.94
3.92
3.92
3.80
3.66
3.63
3.59
3.36
3.04
2.60
1.97
1.95
1.53
1.42
1.35
1.30
OBn
OBn
OBn
HO
N
H
H
O
O
O
O
O
17
13.99
2.07
1.10
4.08
1.11
1.12
1.05
1.09
1.03
1.19
1.32
1.55
3.20
1.11
1.24
1.17
0.86
3.00
3.04
3.01
3.10
8.0
7.5
7.0
6.5
6.0
5.5
5.0
4.5
4.0
3.5
3.0
2.5
2.0
1.5
1.0
0.5
f1 (ppm)

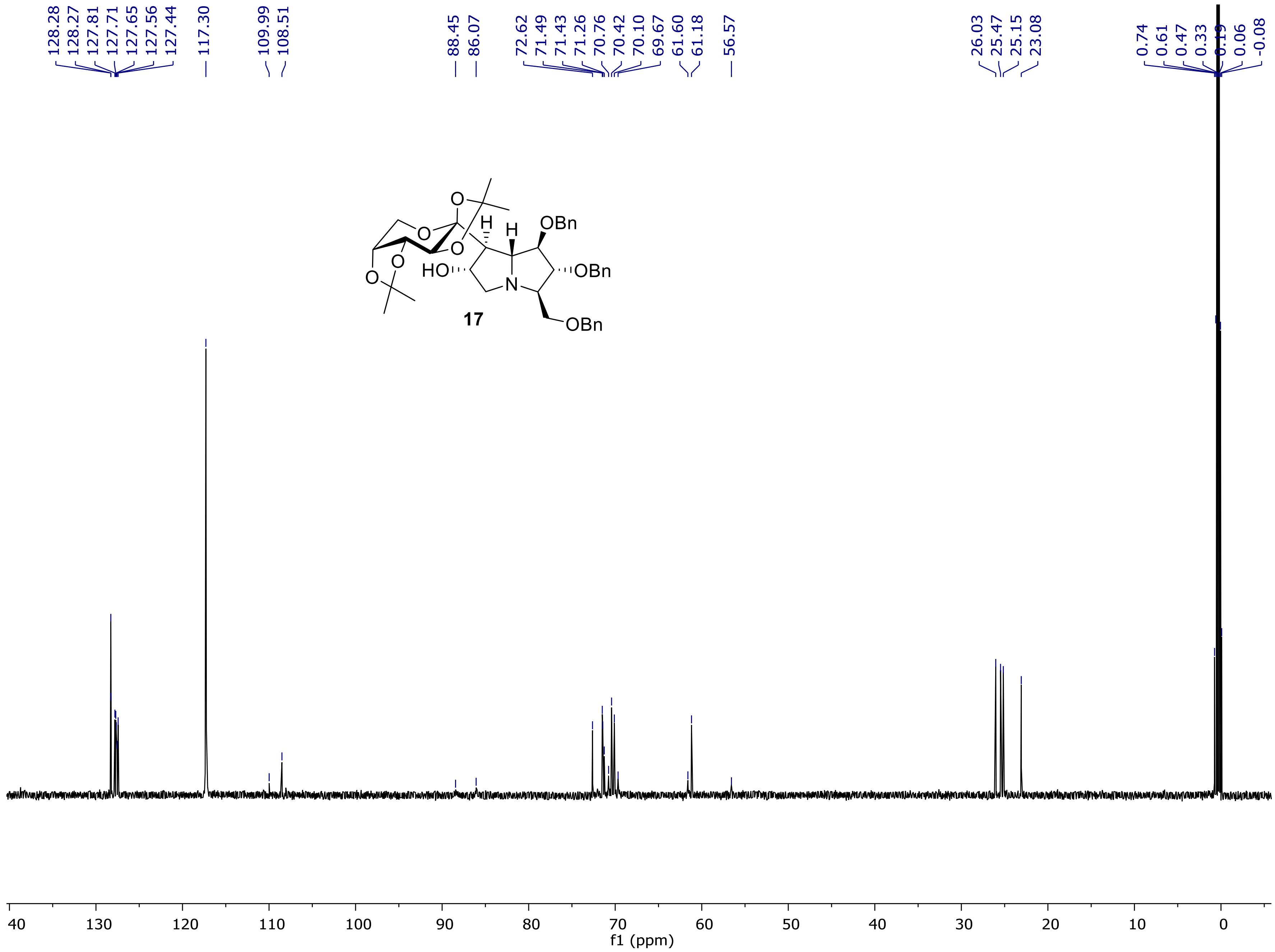
128.28
128.27
127.81
127.71
127.65
127.56
127.44
117.30
109.99
108.51
88.45
86.07
72.62
71.49
71.43
71.26
70.76
70.42
70.10
69.67
61.60
61.18
56.57
26.03
25.47
25.15
23.08
0.74
0.61
0.47
0.33
0.19
0.06
-0.08
OBn
OBn
OBn
HO
H
H
N
O
O
O
O
O
17
f1 (ppm)

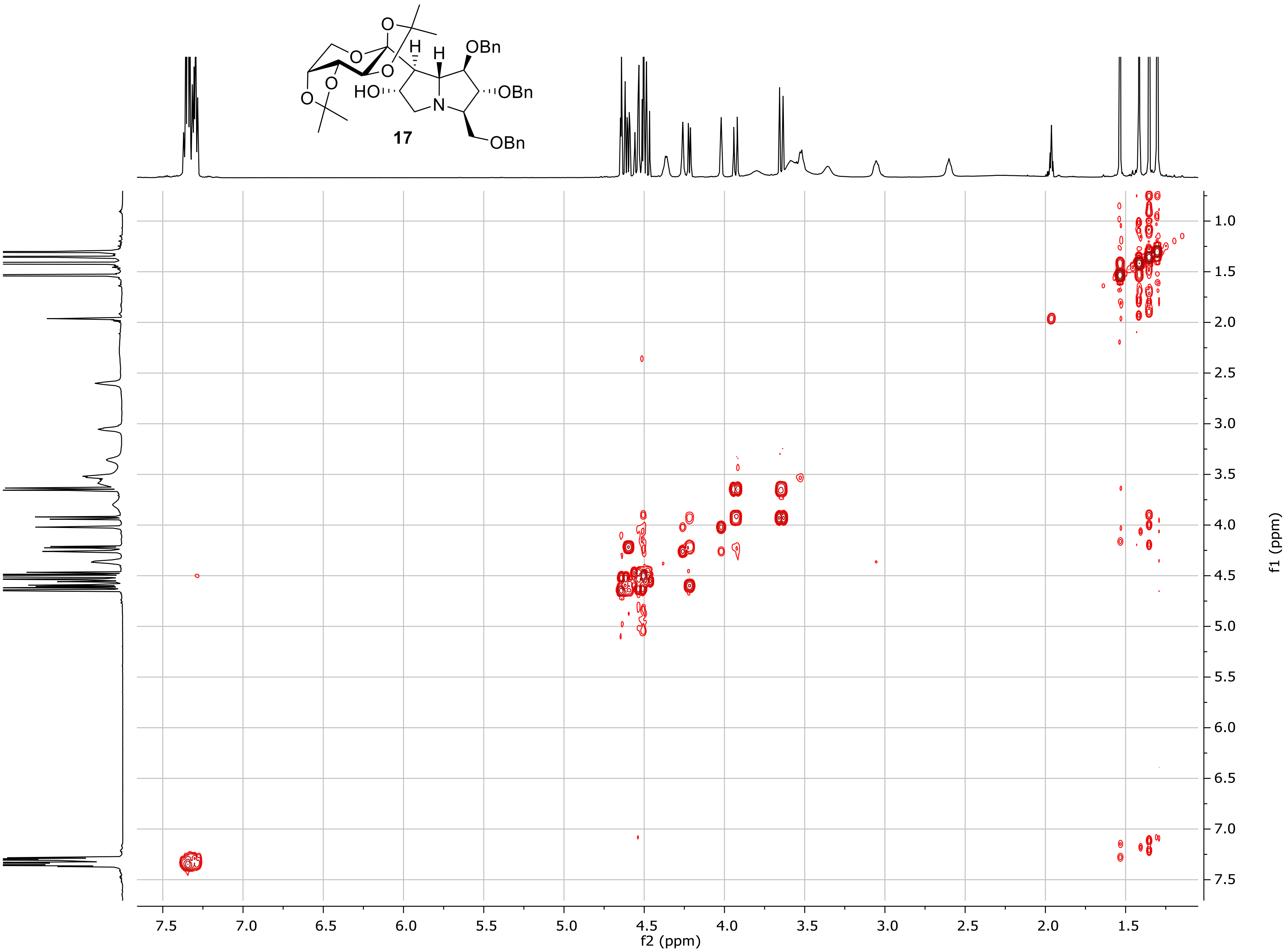

17
OBn
HO
N
f2 (ppm)
f1 (ppm)

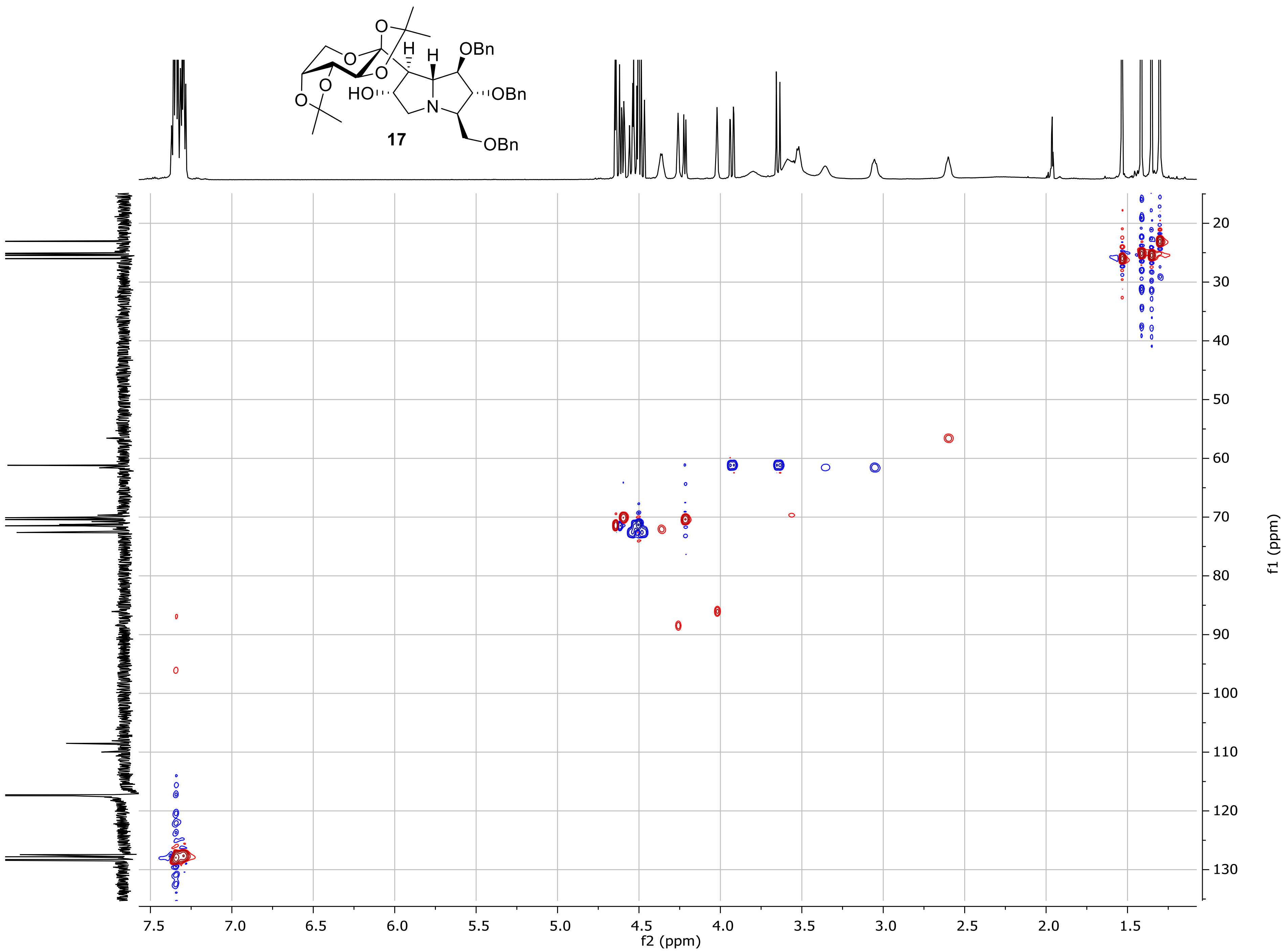

OBn
HO
N
OBn
OBn
17
f2 (ppm)
f1 (ppm)

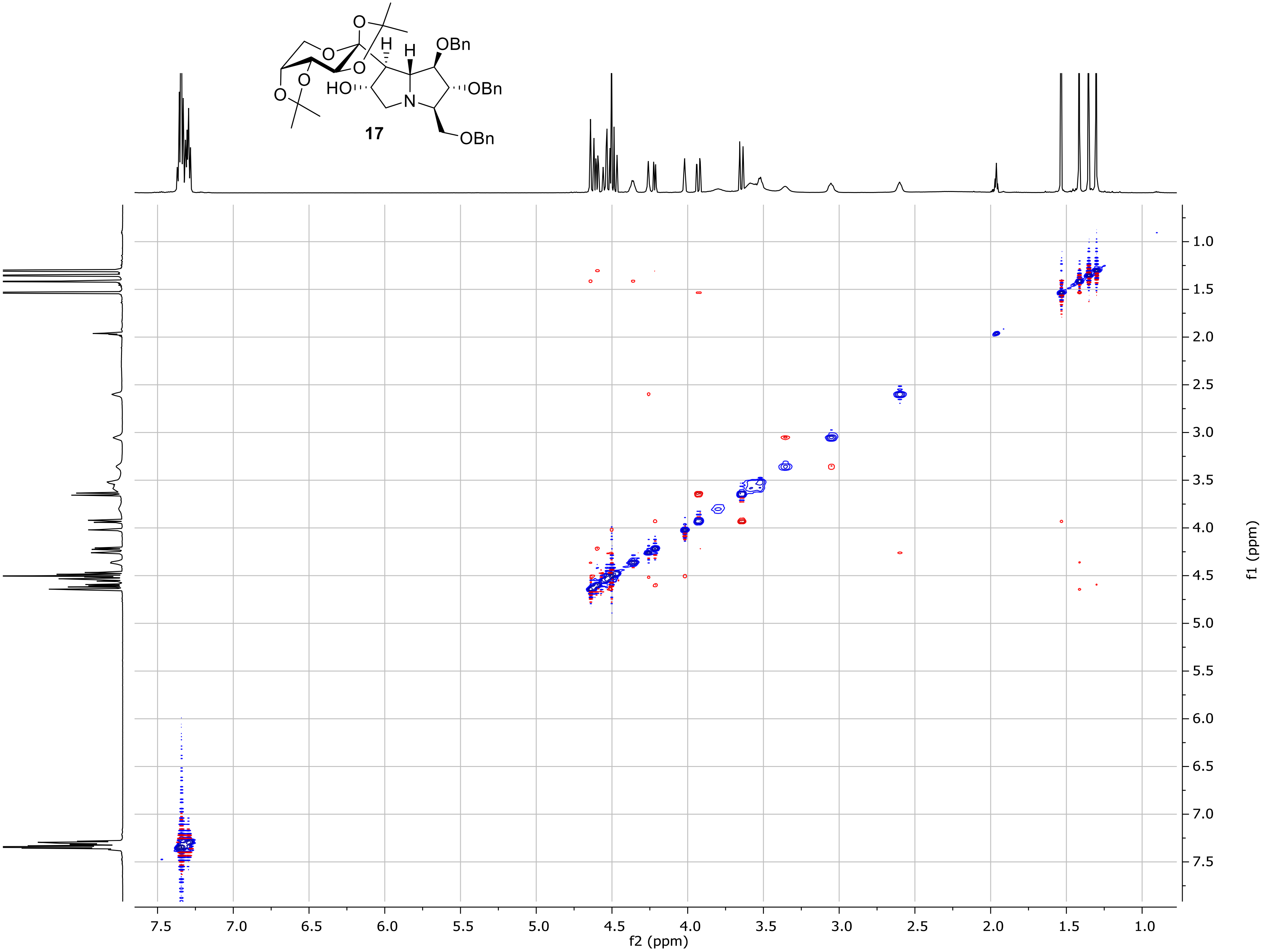
17
f2 (ppm)
f1 (ppm)

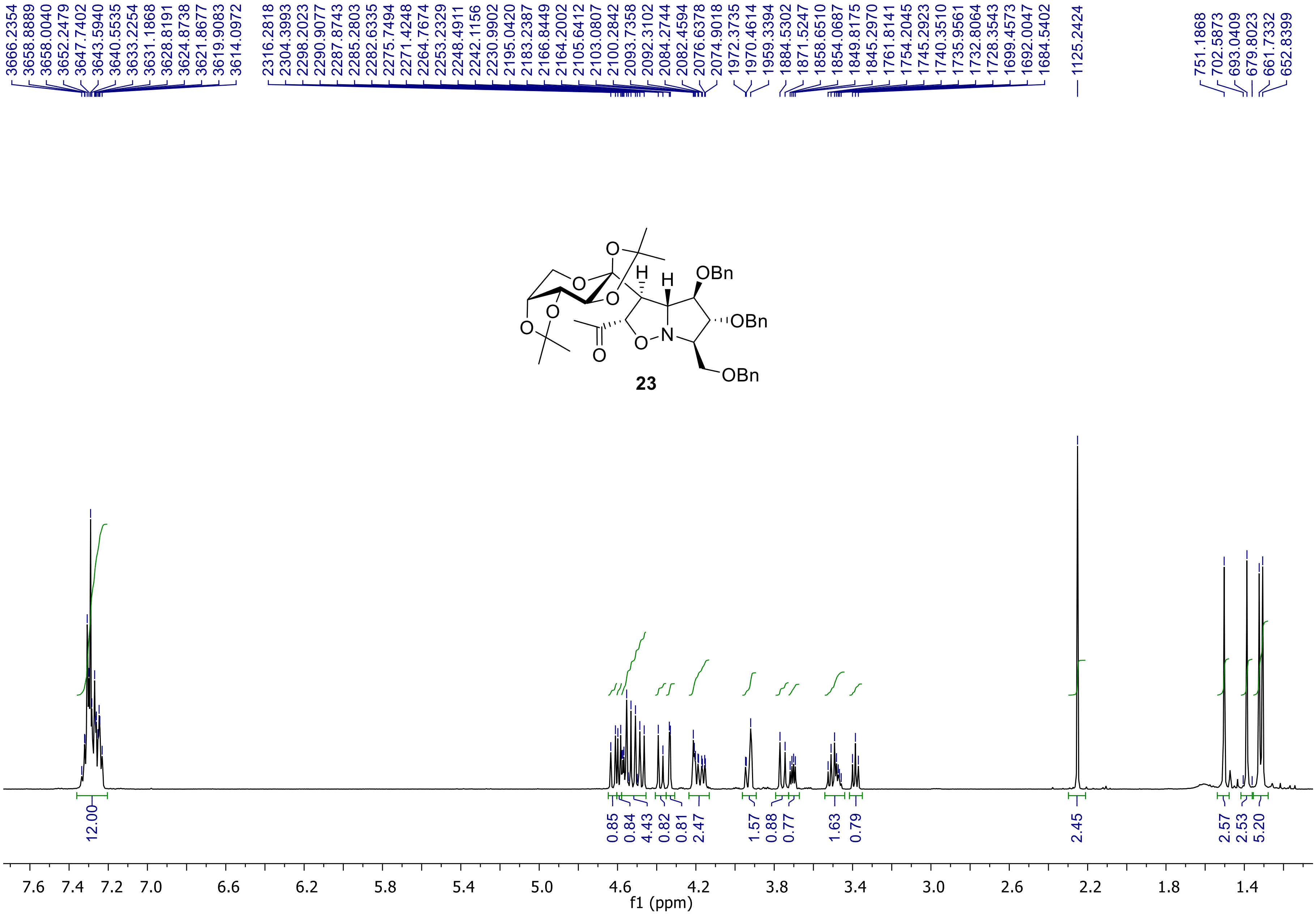
3666.2354
3658.8889
3658.0040
3652.2479
3647.7402
3643.5940
3640.5535
3633.2254
3631.1868
3628.8191
3624.8738
3621.8677
3619.9083
3614.0972
2316.2818
2304.3993
2298.2023
2290.9077
2287.8743
2285.2803
2282.6335
2275.7494
2271.4248
2264.7674
2253.2329
2248.4911
2242.1156
2230.9902
2195.0420
2183.2387
2166.8449
2164.2002
2105.6412
2103.0807
2100.2842
2093.7358
2092.3102
2084.2744
2082.4594
2076.6378
2074.9018
1972.3735
1970.4614
1959.3394
1884.5302
1871.5247
1858.6510
1854.0687
1849.8175
1845.2970
1761.8141
1754.2045
1745.2923
1740.3510
1735.9561
1732.8064
1728.3543
1699.4573
1692.0047
1684.5402
1125.2424
751.1868
702.5873
693.0409
679.8023
661.7332
652.8399
OBn
OBn
OBn
H
H
O
O
O
O
O
O
N
23
12.00
0.85
0.84
4.43
0.82
0.81
2.47
1.57
0.88
0.77
1.63
0.79
2.45
2.57
2.53
5.20
7.6 7.4 7.2 7.0 6.6 6.2 5.8 5.4 5.0 4.6 4.2 3.8 3.4 3.0 2.6 2.2 1.8 1.4
f1 (ppm)

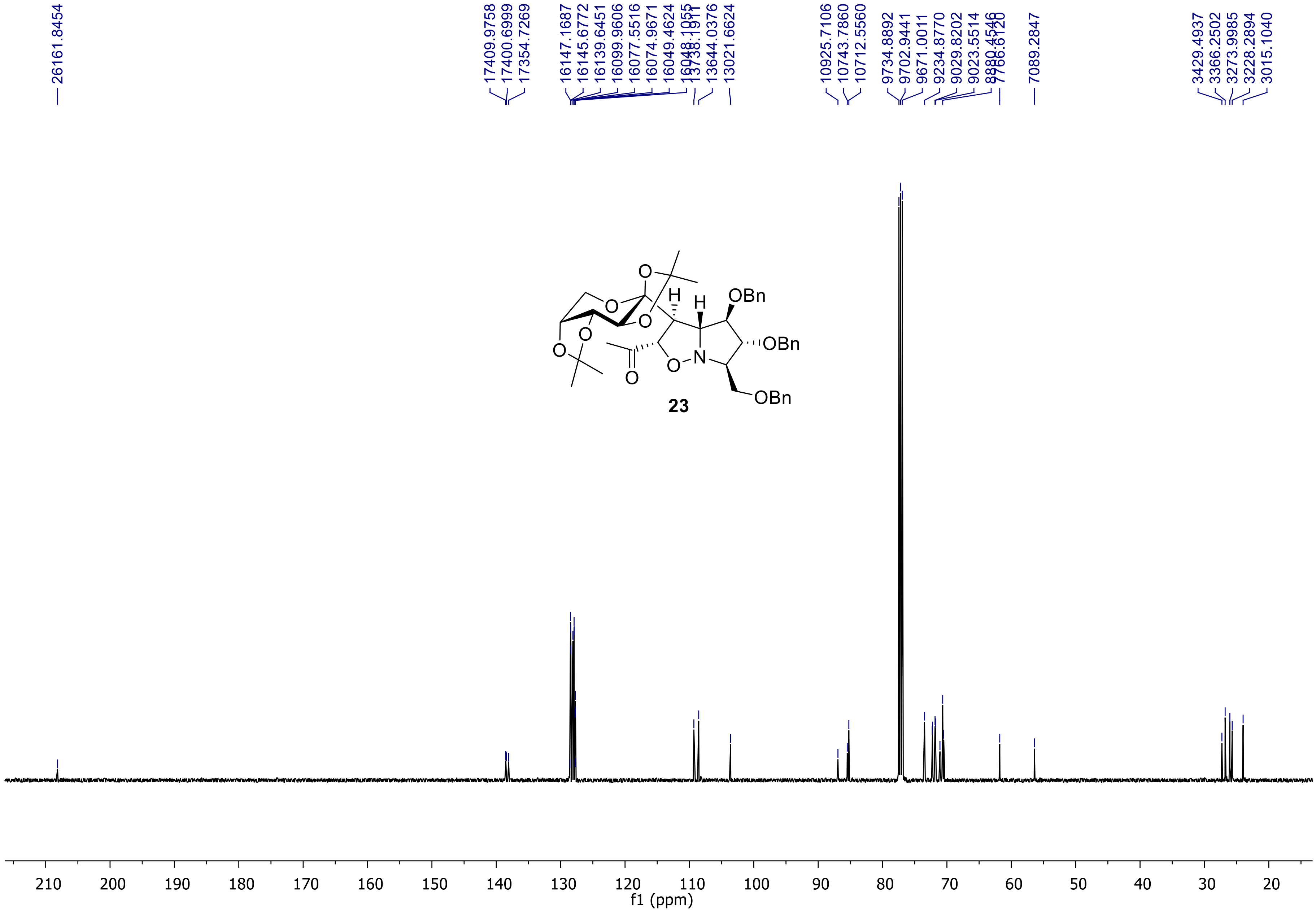
26161.8454
17409.9758
17400.6999
17354.7269
16147.1687
16145.6772
16139.6451
16099.9606
16077.5516
16074.9671
16049.4624
16048.1055
13644.0376
13021.6624
10925.7106
10743.7860
10712.5560
9734.8892
9702.9441
9671.0011
9234.8770
9029.8202
9023.5514
7089.2847
3429.4937
3366.2502
3273.9985
3228.2894
3015.1040
OBn
OBn
OBn
23
f1 (ppm)

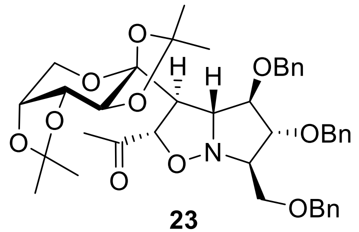

23

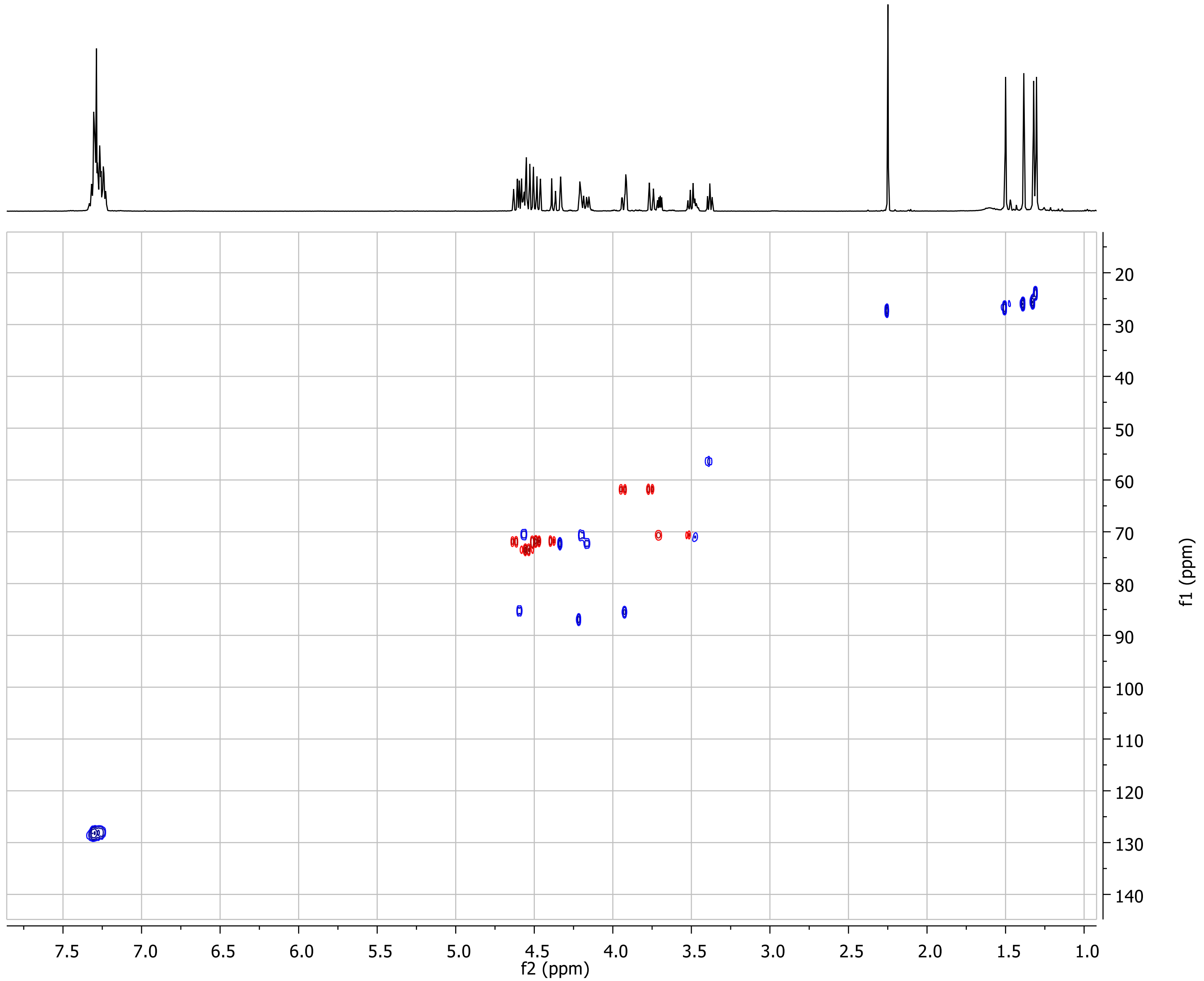

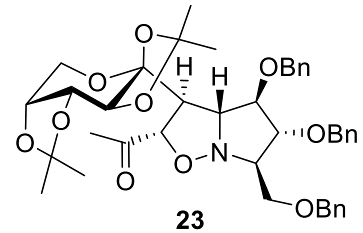

23

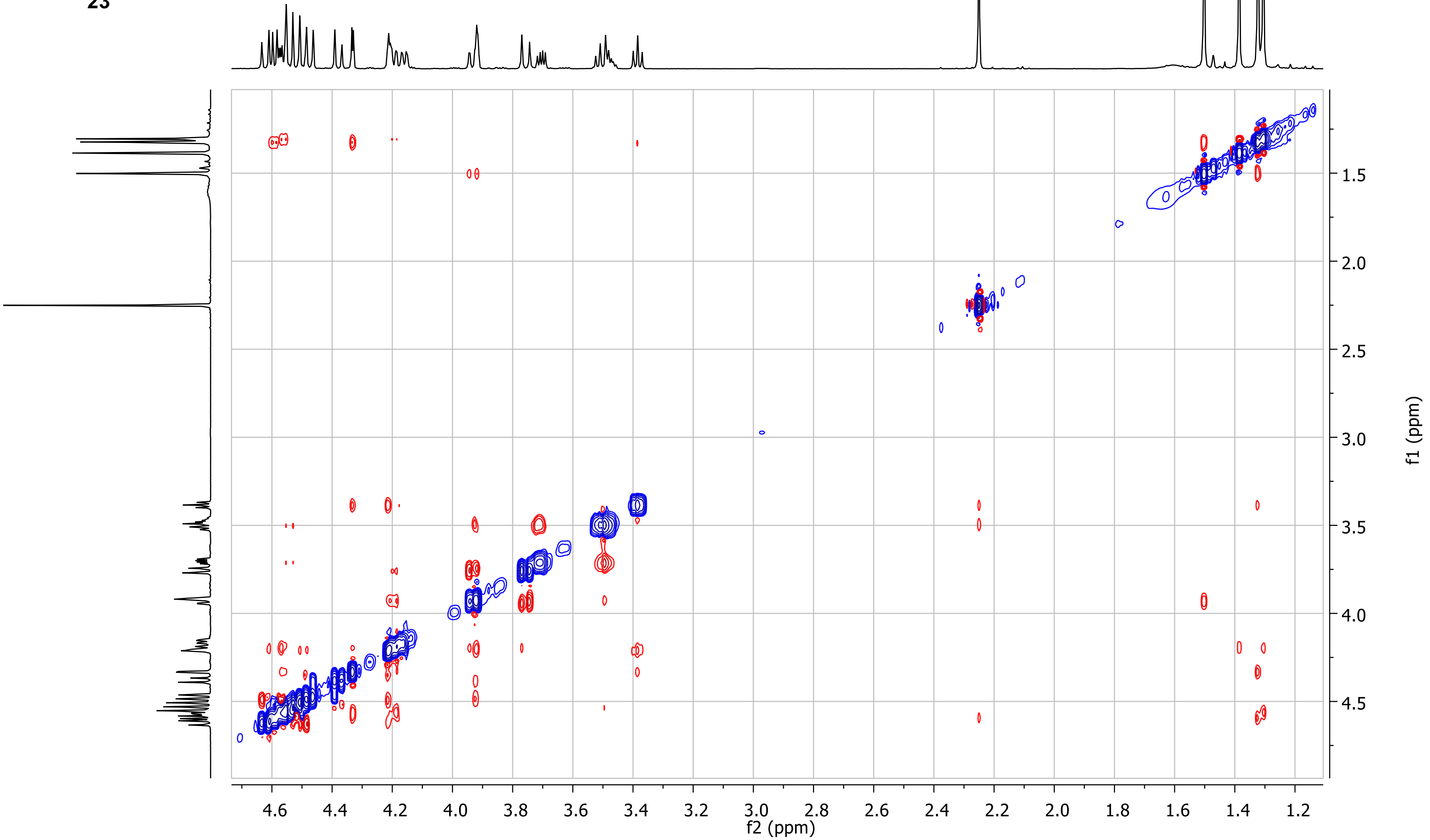

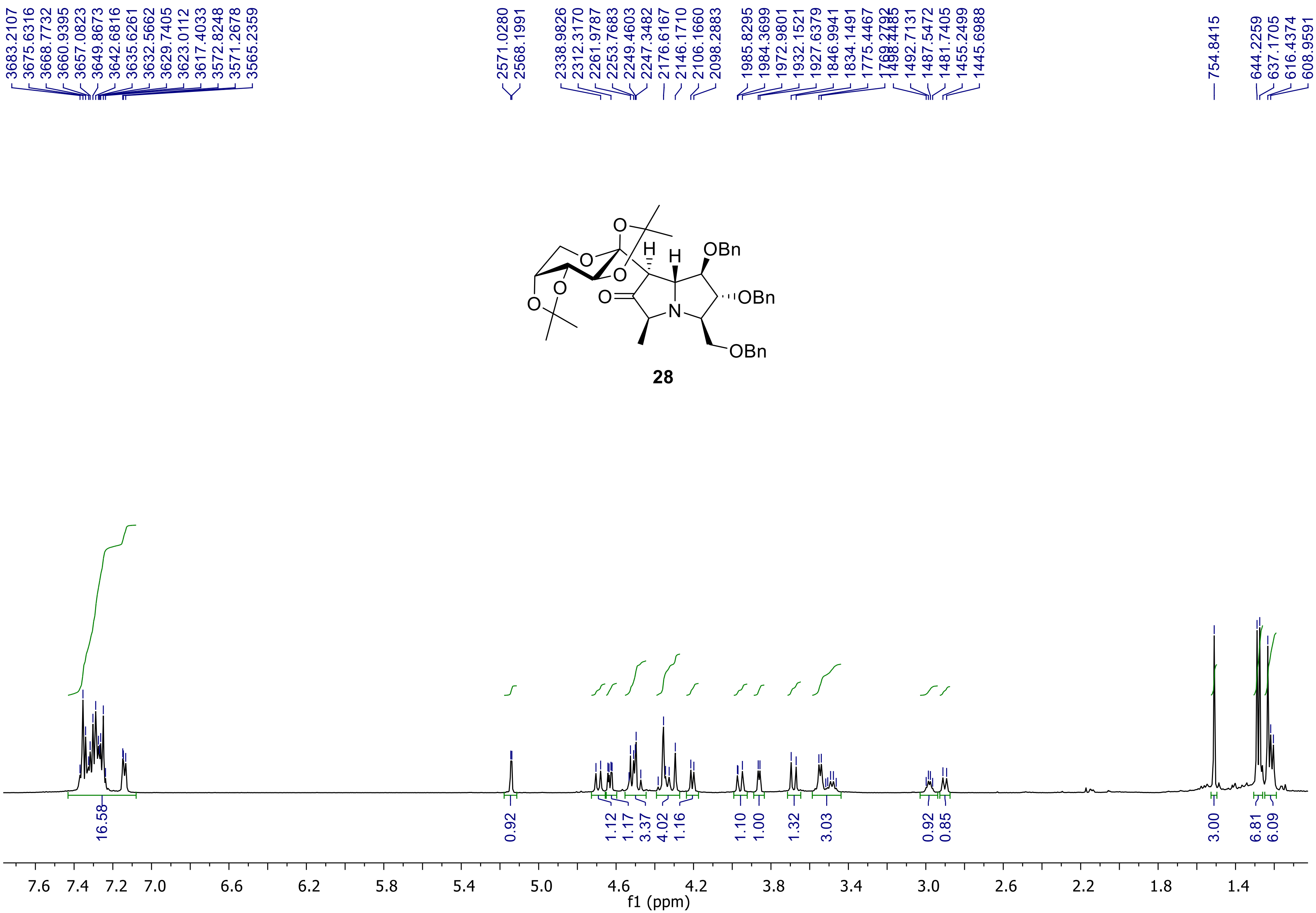

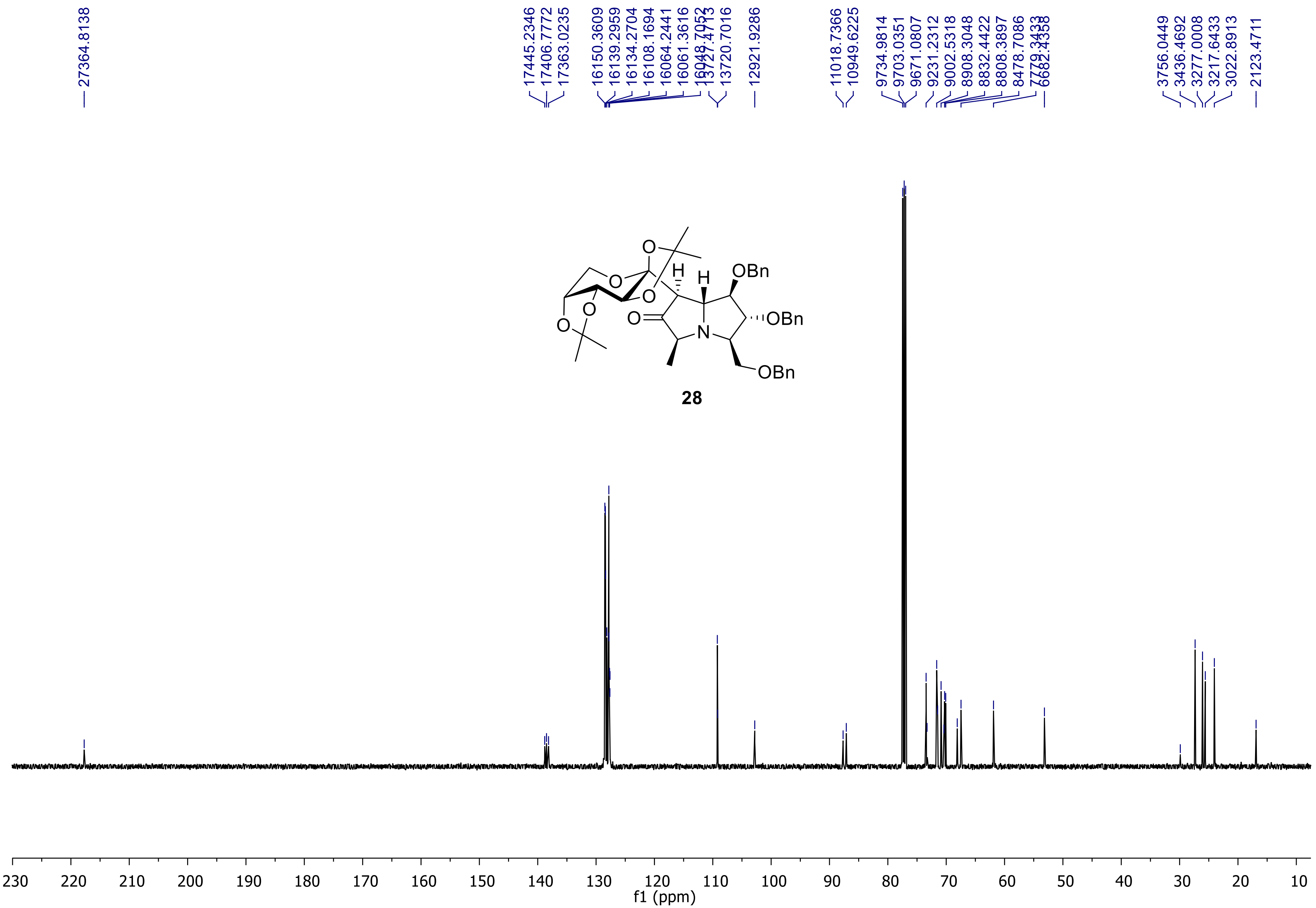
27364.8138
17445.2346
17406.7772
17363.0235
16150.3609
16139.2959
16134.2704
16108.1694
16064.2441
16061.3616
13720.7016
12921.9286
11018.7366
10949.6225
9734.9814
9703.0351
9671.0807
9231.2312
9002.5318
8908.3048
8832.4422
8808.3897
8478.7086
7779.3433
6682.4358
3756.0449
3436.4692
3277.0008
3217.6433
3022.8913
2123.4711
OBn
OBn
OBn
28
f1 (ppm)

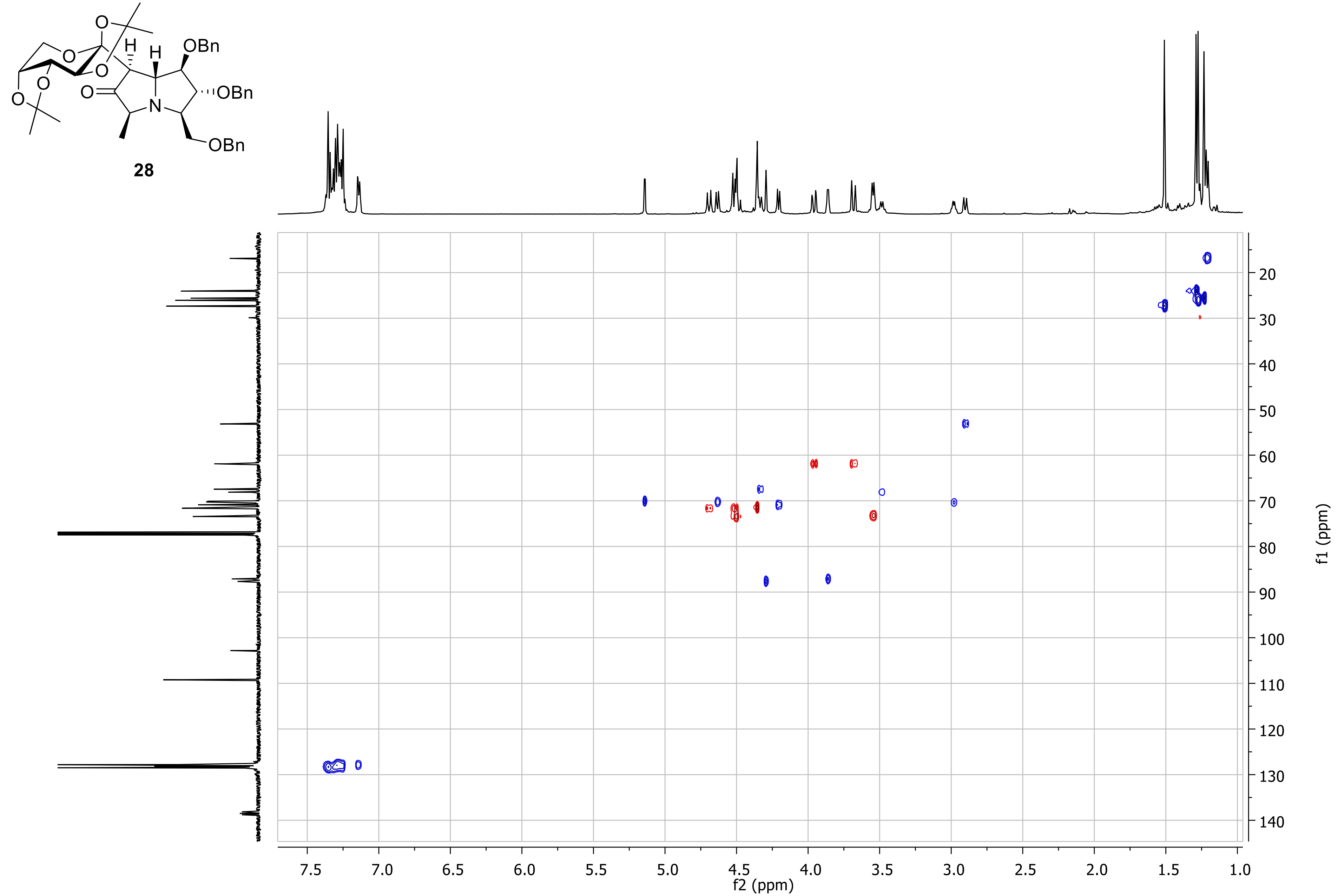

O
O
O
O
O
H
H
OBn
O
N
OBn
OBn
28
f2 (ppm)
f1 (ppm)

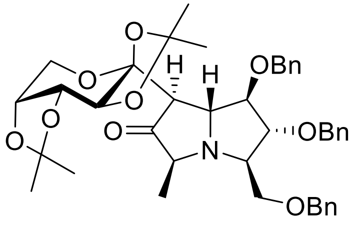

28

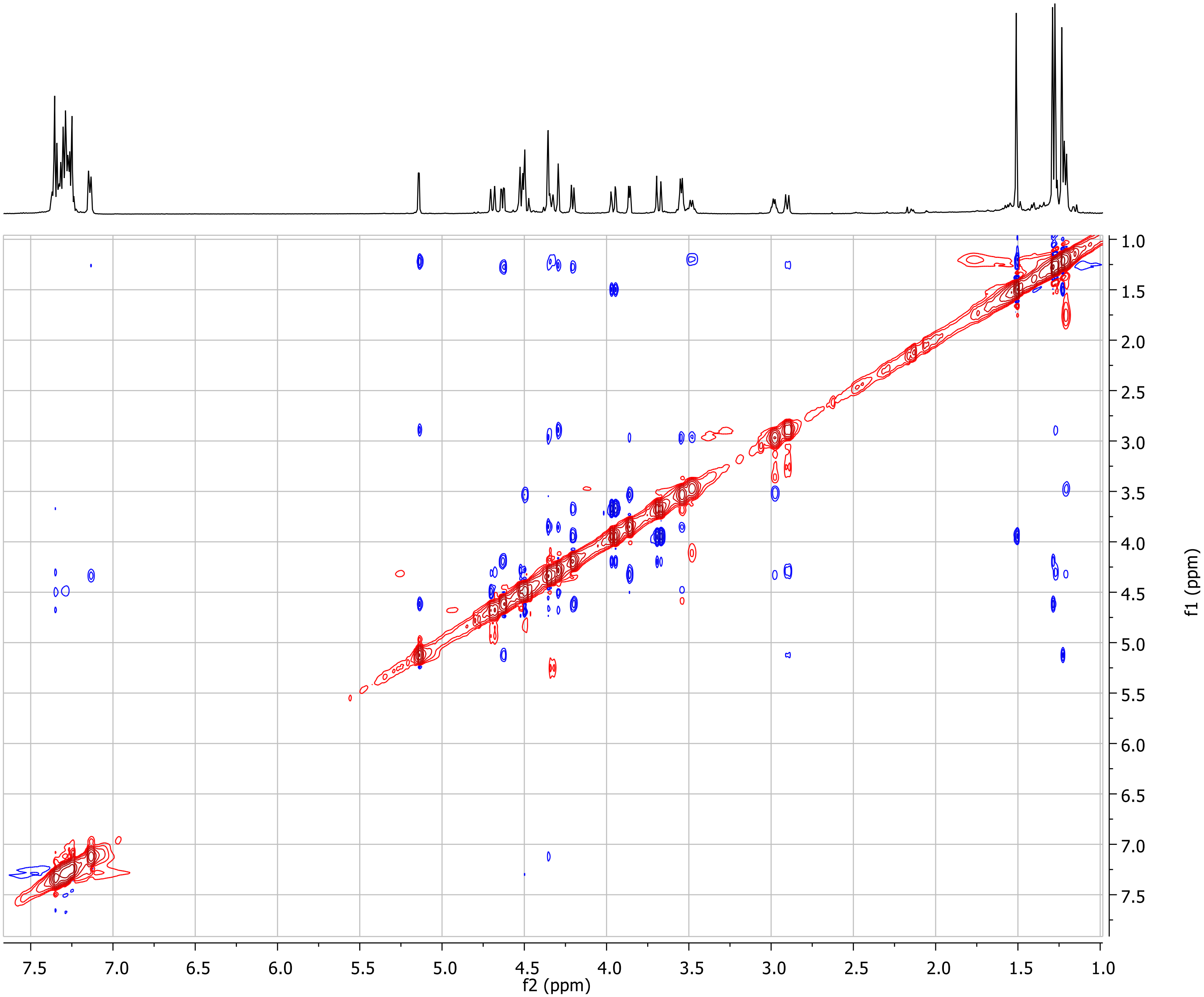

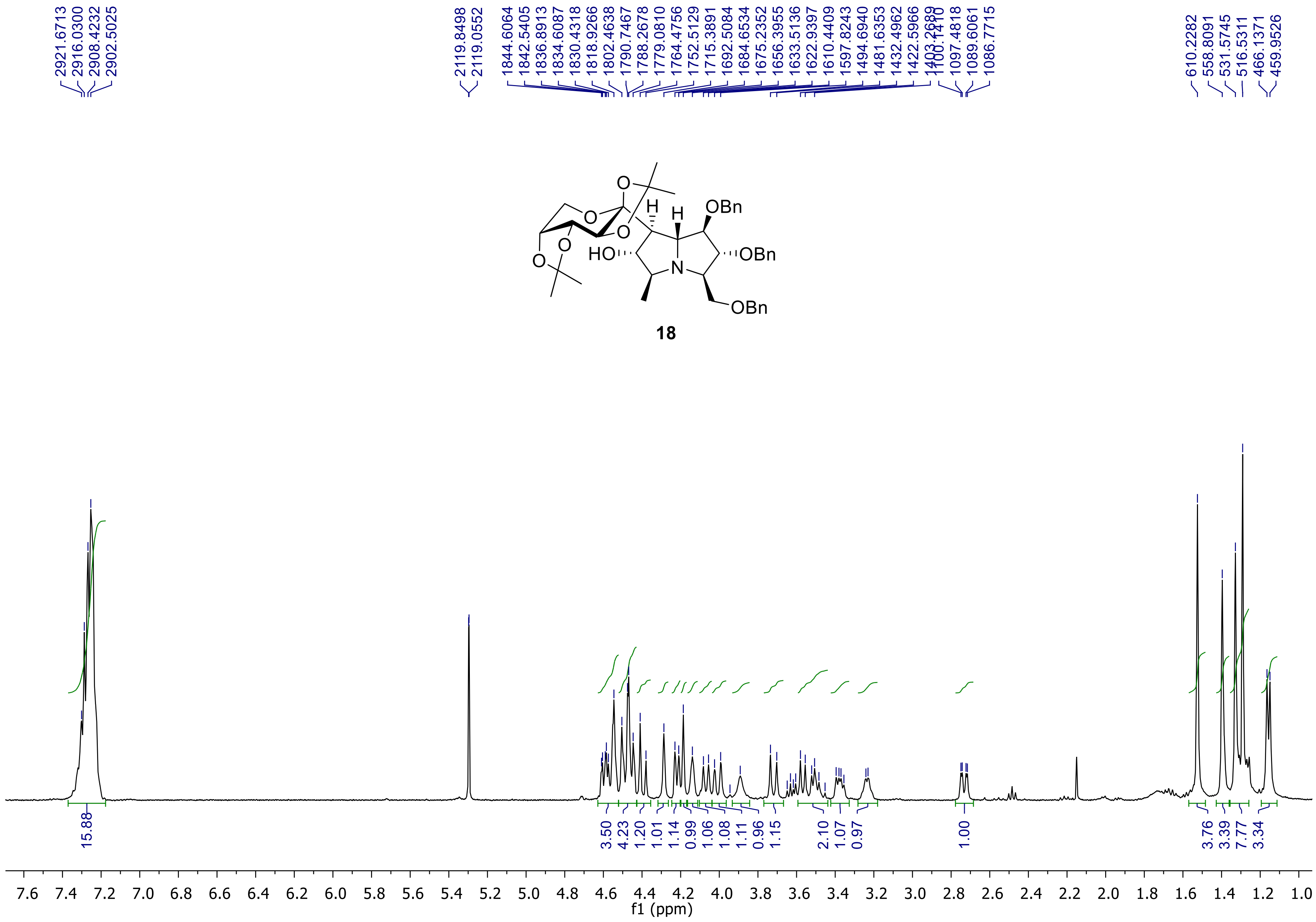
2921.6713
2916.0300
2908.4232
2902.5025
2119.8498
2119.0552
1844.6064
1842.5405
1836.8913
1834.6087
1830.4318
1818.9266
1802.4638
1790.7467
1788.2678
1779.0810
1764.4756
1752.5129
1715.3891
1692.5084
1684.6534
1675.2352
1656.3955
1633.5136
1622.9397
1610.4409
1597.8243
1494.6940
1481.6353
1432.4962
1422.5966
1097.4818
1089.6061
1086.7715
610.2282
558.8091
531.5745
516.5311
466.1371
459.9526
OBn
HO
OBn
N
OBn
18
15.88
3.50
4.23
1.20
1.01
1.14
0.99
1.06
1.08
1.11
0.96
1.15
2.10
1.07
0.97
1.00
3.76
3.39
7.77
3.34
7.6 7.4 7.2 7.0 6.8 6.6 6.4 6.2 6.0 5.8 5.6 5.4 5.2 5.0 4.8 4.6 4.4 4.2 4.0 3.8 3.6 3.4 3.2 3.0 2.8 2.6 2.4 2.2 2.0 1.8 1.6 1.4 1.2 1.0
f1 (ppm)

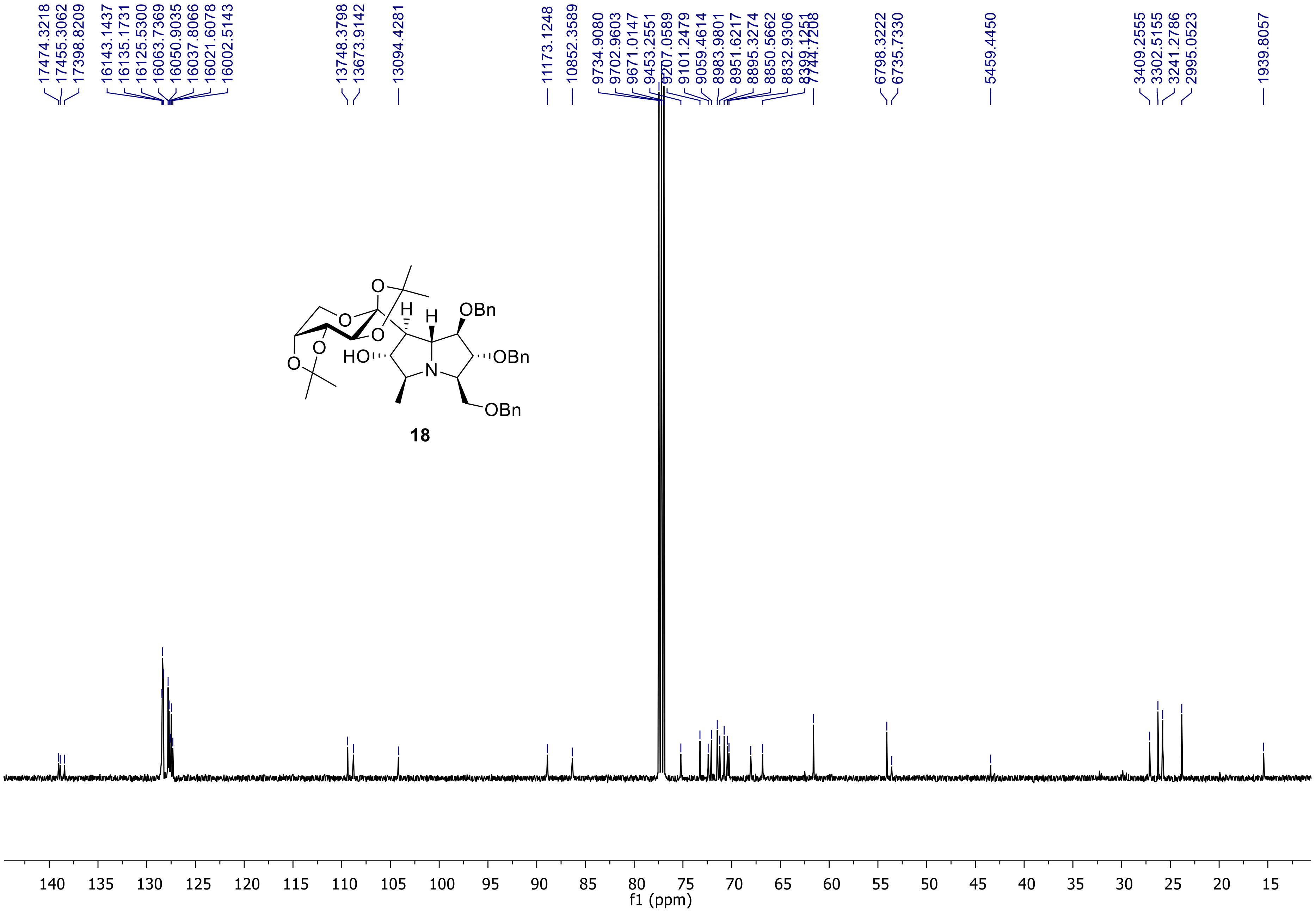
17474.3218
17455.3062
17398.8209
16143.1437
16135.1731
16125.5300
16063.7369
16050.9035
16037.8066
16021.6078
16002.5143
13748.3798
13673.9142
13094.4281
11173.1248
10852.3589
9734.9080
9702.9603
9671.0147
9453.2551
9207.0589
9101.2479
9059.4614
8983.9801
8951.6217
8895.3274
8850.5662
8832.9306
6798.3222
6735.7330
5459.4450
3409.2555
3302.5155
3241.2786
2995.0523
1939.8057
OBn
OBn
OBn
HO
N
H
H
O
O
O
O
O
18
140 135 130 125 120 115 110 105 100 95 90 85 80 75 70 65 60 55 50 45 40 35 30 25 20 15
f1 (ppm)

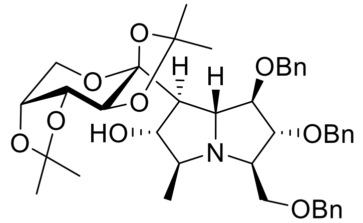
O
O
O
O
H
H
OBn
HO
OBn
N
OBn

18

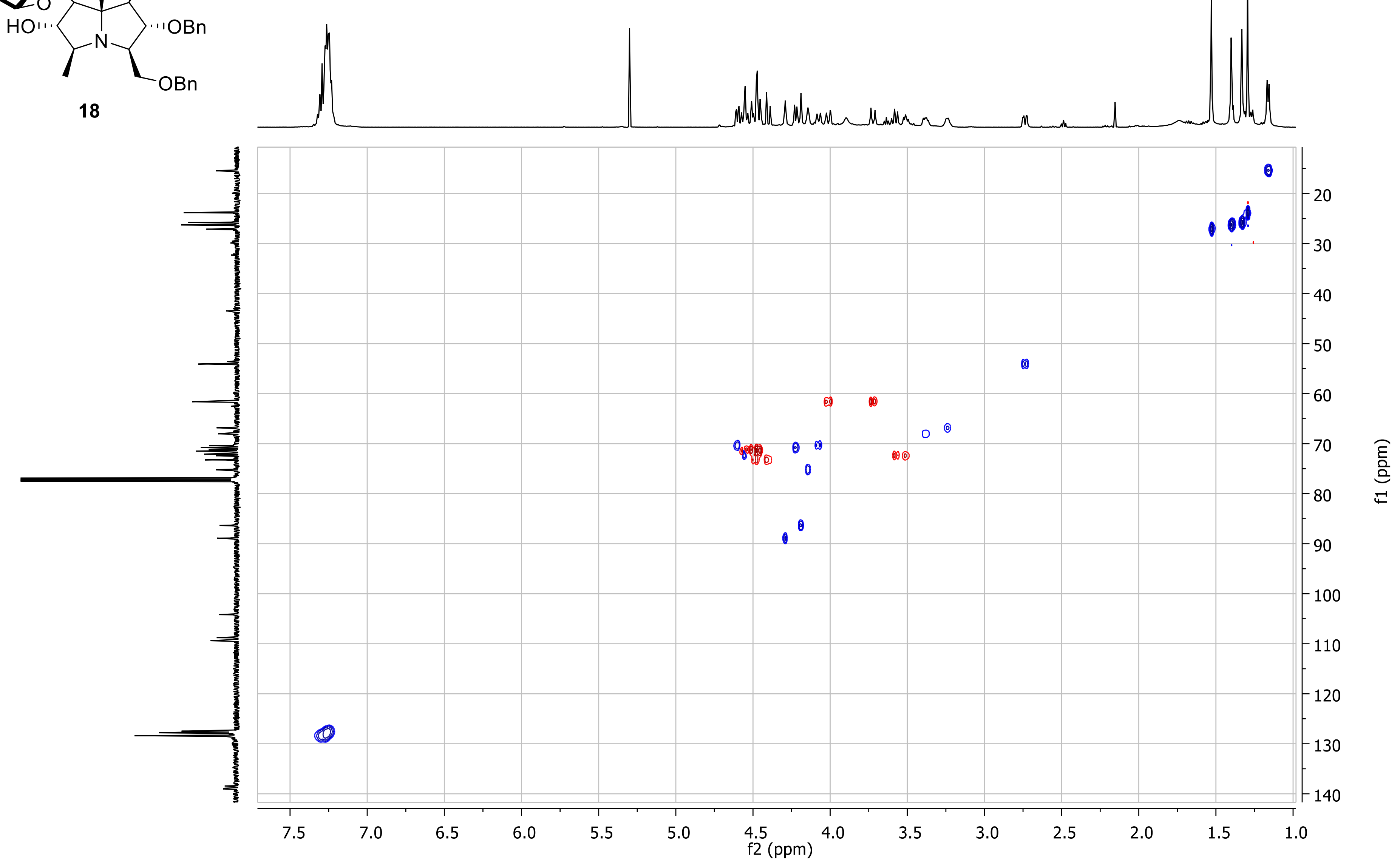
7.5
7.0
6.5
6.0
5.5
5.0
4.5
4.0
3.5
3.0
2.5
2.0
1.5
1.0
f2 (ppm)
20
30
40
50
60
70
80
90
100
110
120
130
140
f1 (ppm)

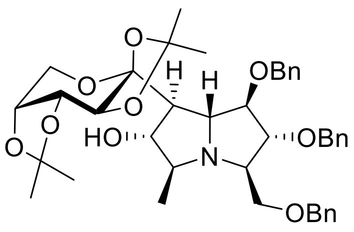

18

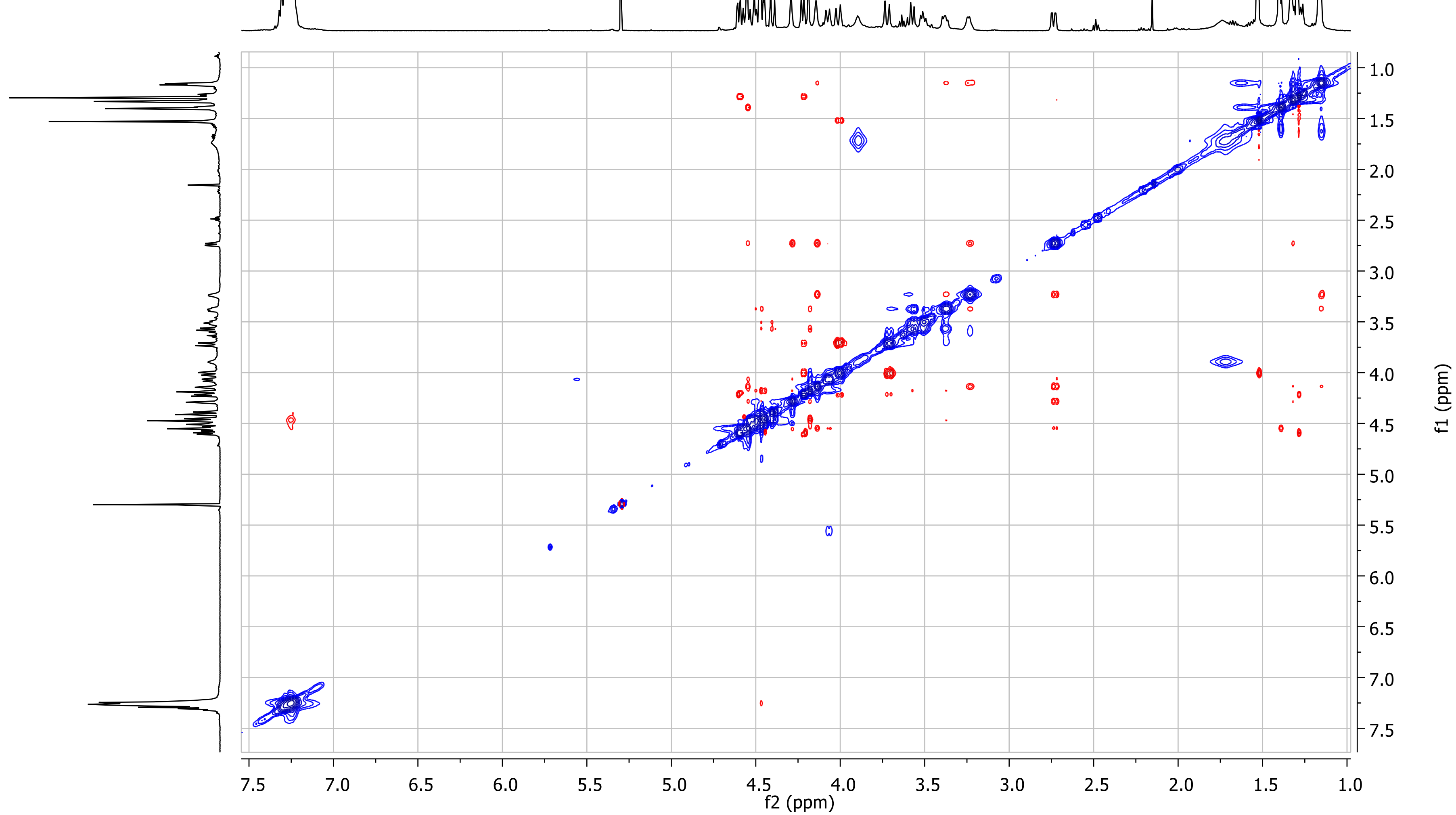

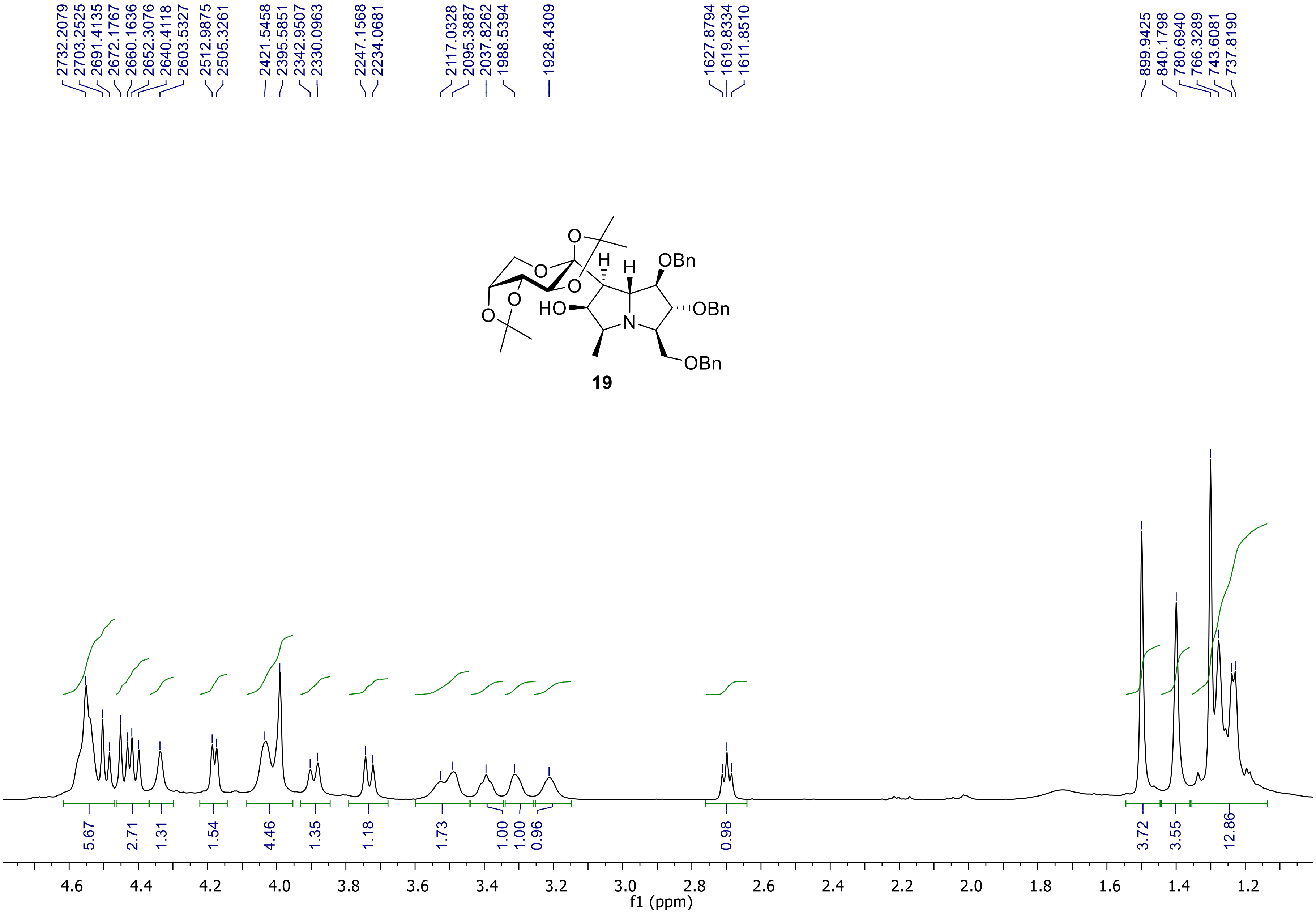

2732.2079
2703.2525
2691.4135
2672.1767
2660.1636
2652.3076
2640.4118
2603.5327
2512.9875
2505.3261
2421.5458
2395.5851
2342.9507
2330.0963
2247.1568
2234.0681
2117.0328
2095.3887
2037.8262
1988.5394
1928.4309
1627.8794
1619.8334
1611.8510
899.9425
840.1798
780.6940
766.3289
743.6081
737.8190
OBn
OBn
OBn
HO
N
H
H
O
O
O
O
O
O
19
5.67
2.71
1.31
1.54
4.46
1.35
1.18
1.73
1.00
1.00
0.96
0.98
3.72
3.55
12.86
4.6
4.4
4.2
4.0
3.8
3.6
3.4
3.2
3.0
2.8
2.6
2.4
2.2
2.0
1.8
1.6
1.4
1.2
f1 (ppm)

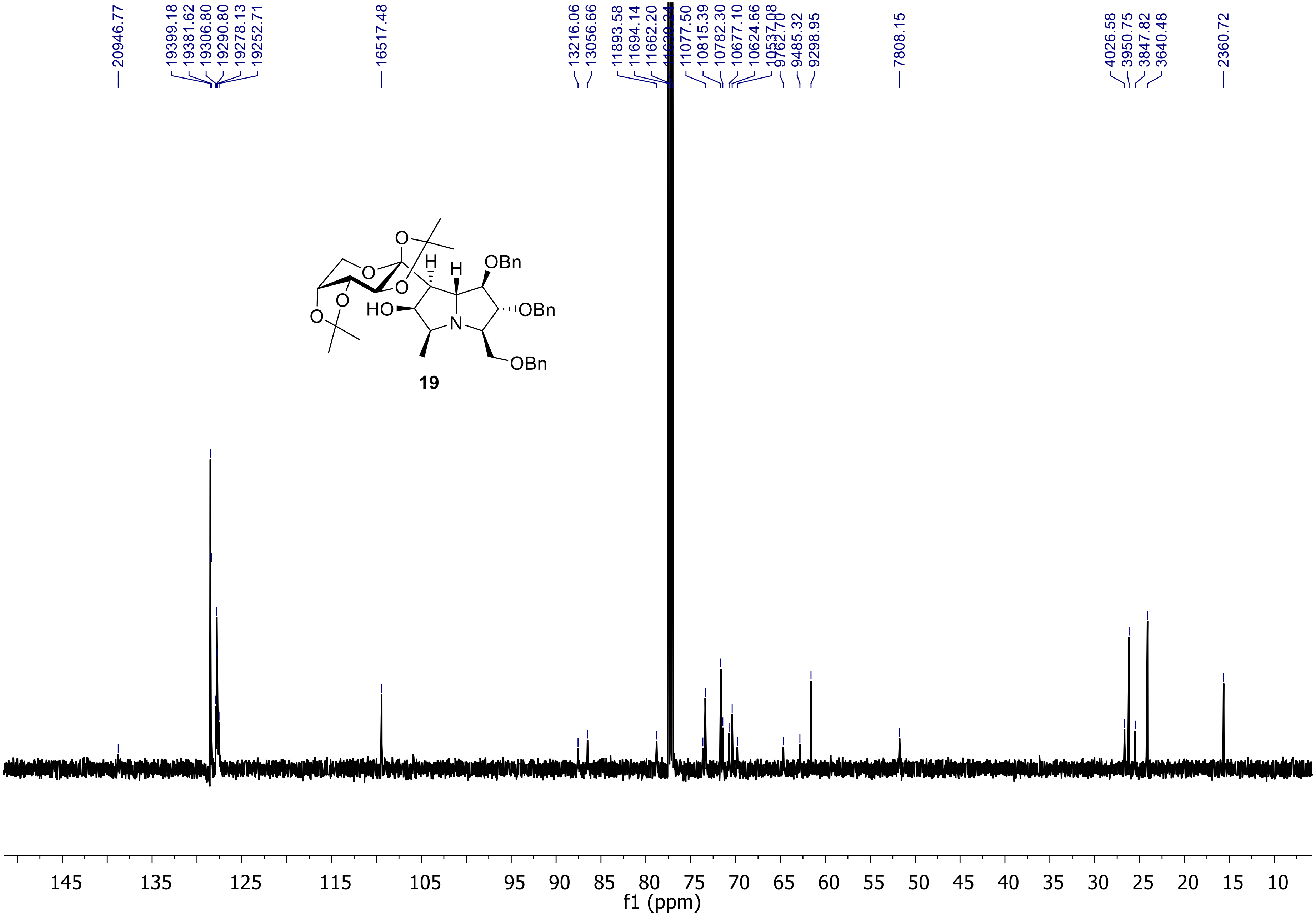
20946.77
19399.18
19381.62
19306.80
19290.80
19278.13
19252.71
16517.48
13216.06
13056.66
11893.58
11694.14
11662.20
11077.50
10815.39
10782.30
10677.10
10624.66
9485.32
9298.95
7808.15
4026.58
3950.75
3847.82
3640.48
2360.72
OBn
OBn
OBn
HO
N
H
H
19
145
135
125
115
105
95
90
85
80
75
70
65
60
55
50
45
40
35
30
25
20
15
10
f1 (ppm)

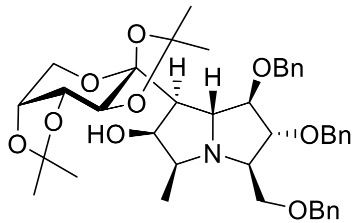
OBn
OBn
OBn
HO
N
H
H
O
O
O
O
O
O

19

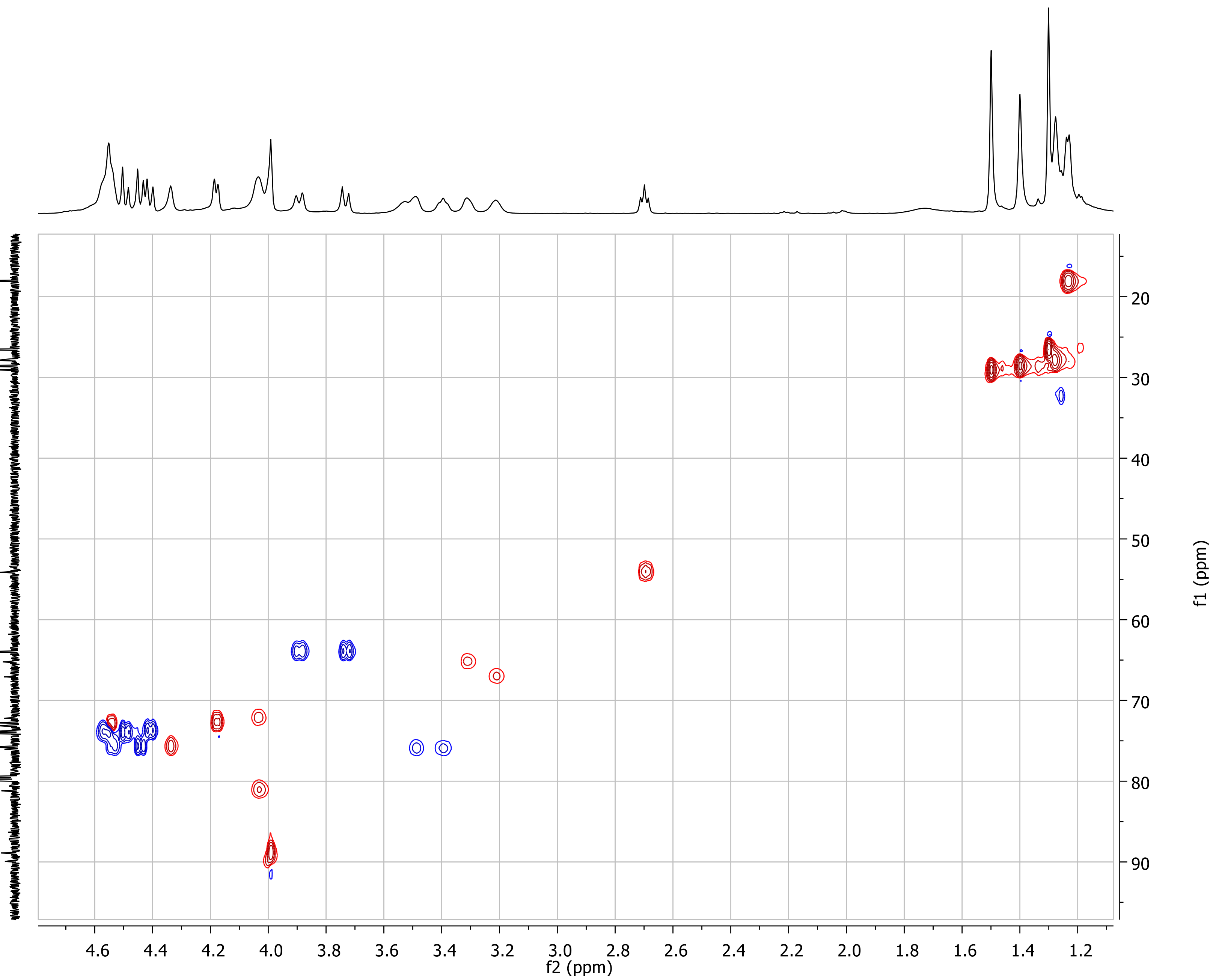
f2 (ppm)
f1 (ppm)
4.6
4.4
4.2
4.0
3.8
3.6
3.4
3.2
3.0
2.8
2.6
2.4
2.2
2.0
1.8
1.6
1.4
1.2
20
30
40
50
60
70
80
90

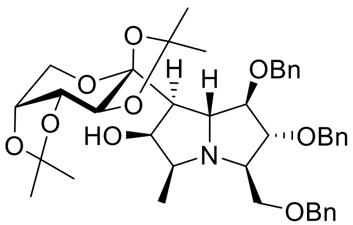
O
O
O
O
O
H
H
OBn
HO
OBn
N
OBn

19

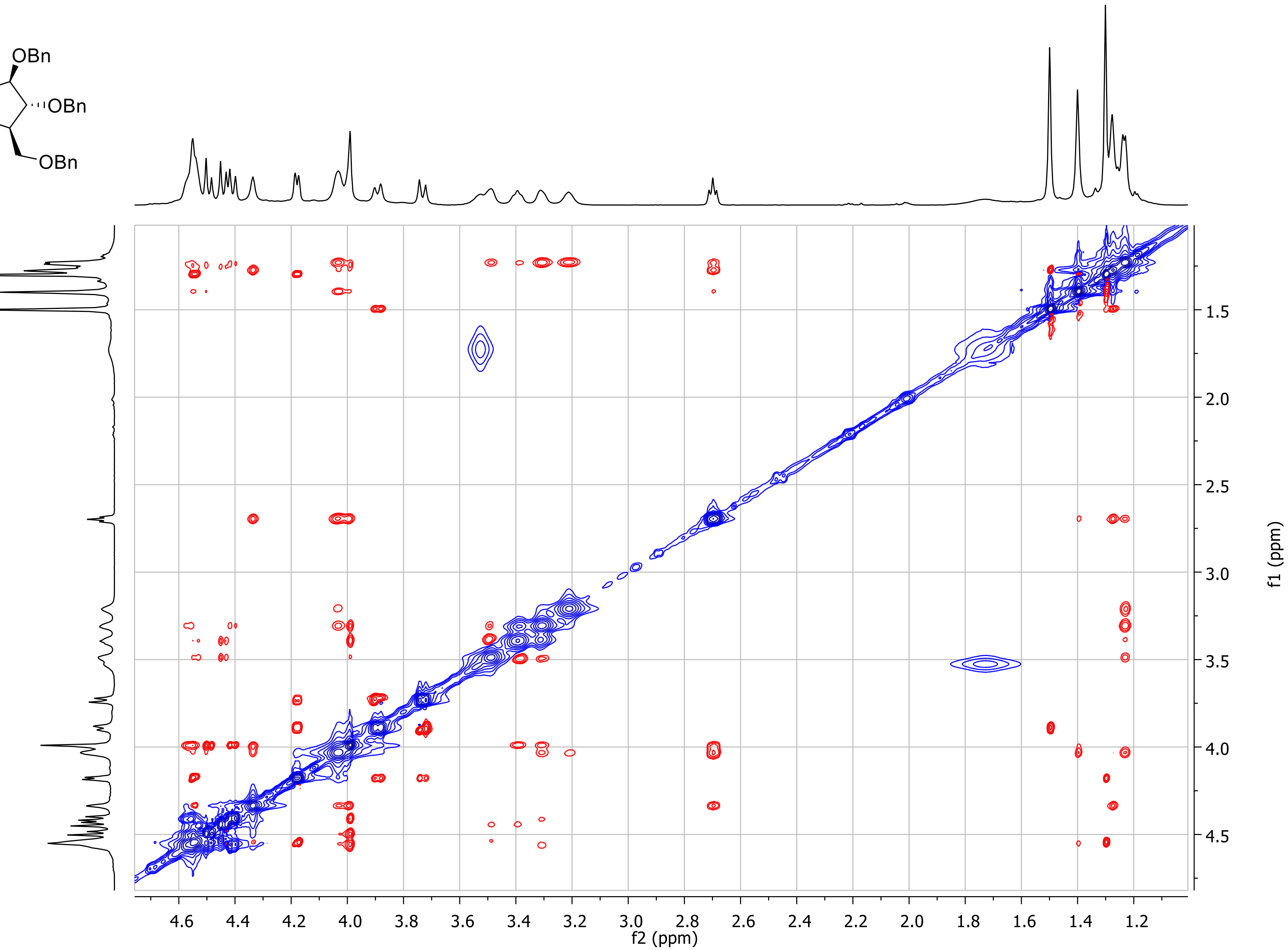
4.6
4.4
4.2
4.0
3.8
3.6
3.4
3.2
3.0
2.8
2.6
2.4
2.2
2.0
1.8
1.6
1.4
1.2
f2 (ppm)
1.5
2.0
2.5
3.0
3.5
4.0
4.5
f1 (ppm)

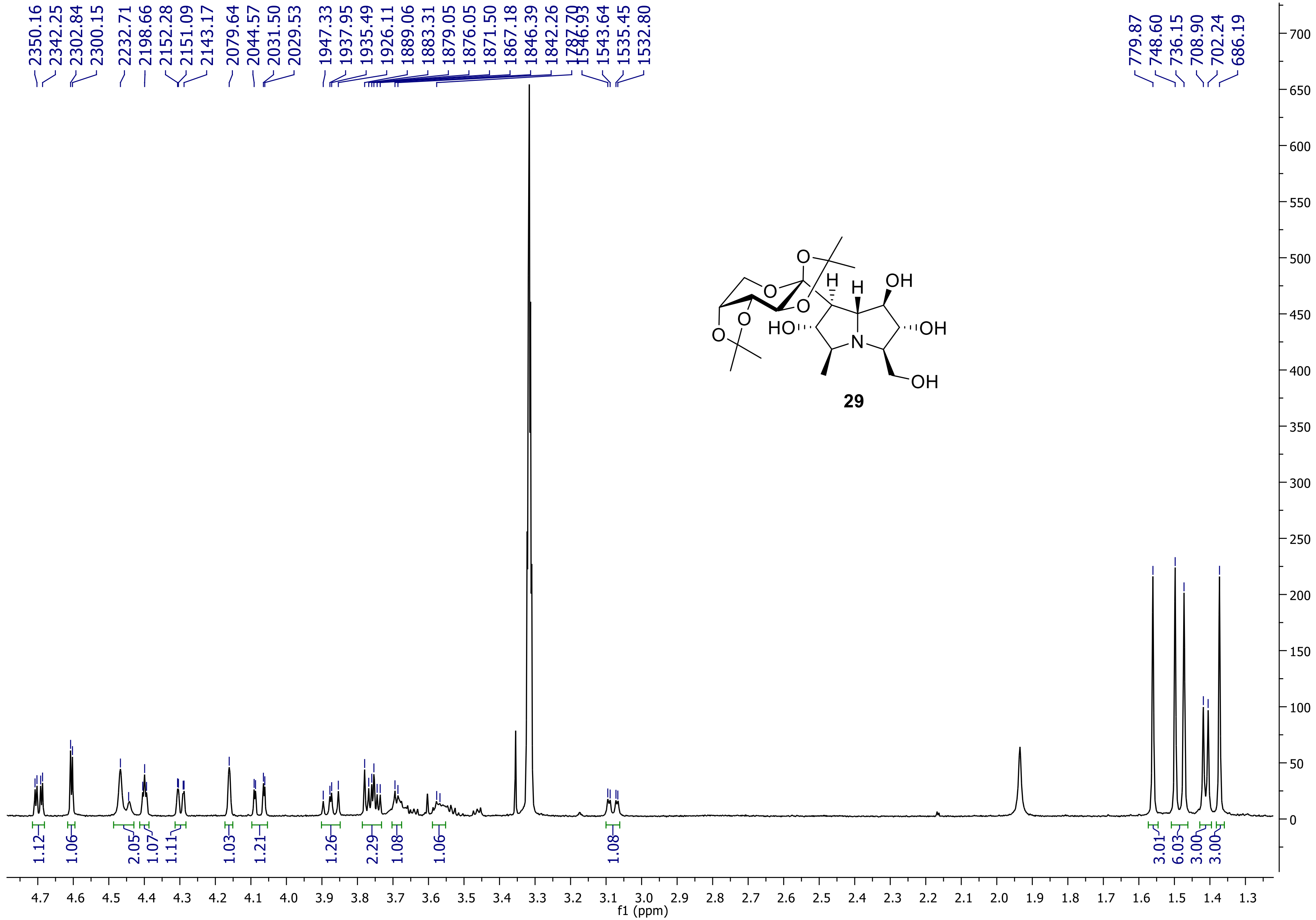

29

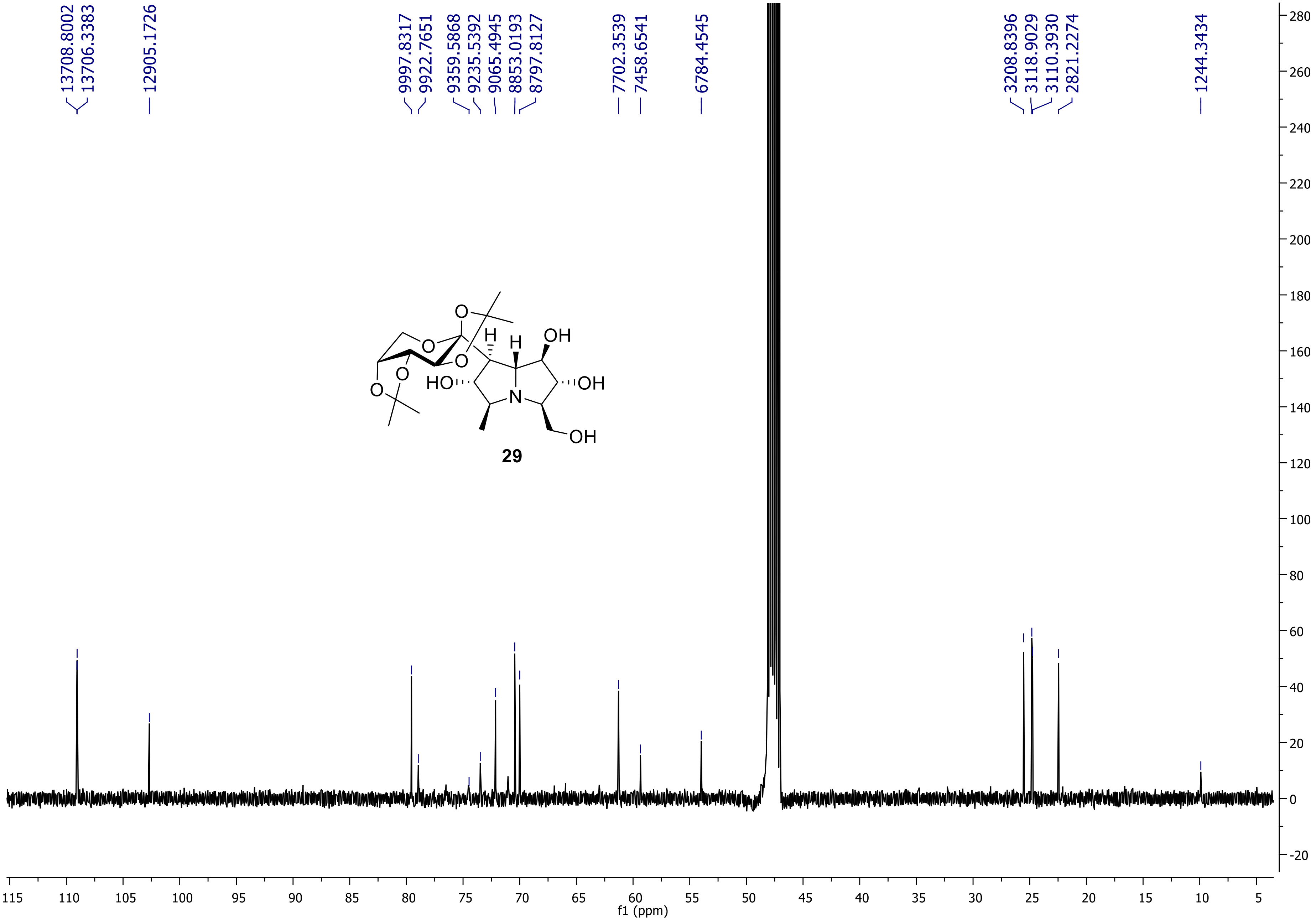
13708.8002
13706.3383
12905.1726
9997.8317
9922.7651
9359.5868
9235.5392
9065.4945
8853.0193
8797.8127
7702.3539
7458.6541
6784.4545
3208.8396
3118.9029
3110.3930
2821.2274
1244.3434
OH
H
H
O
O
O
O
O
HO
OH
N
OH
29
f1 (ppm)

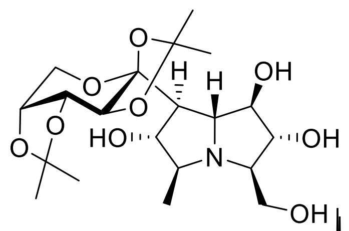

29

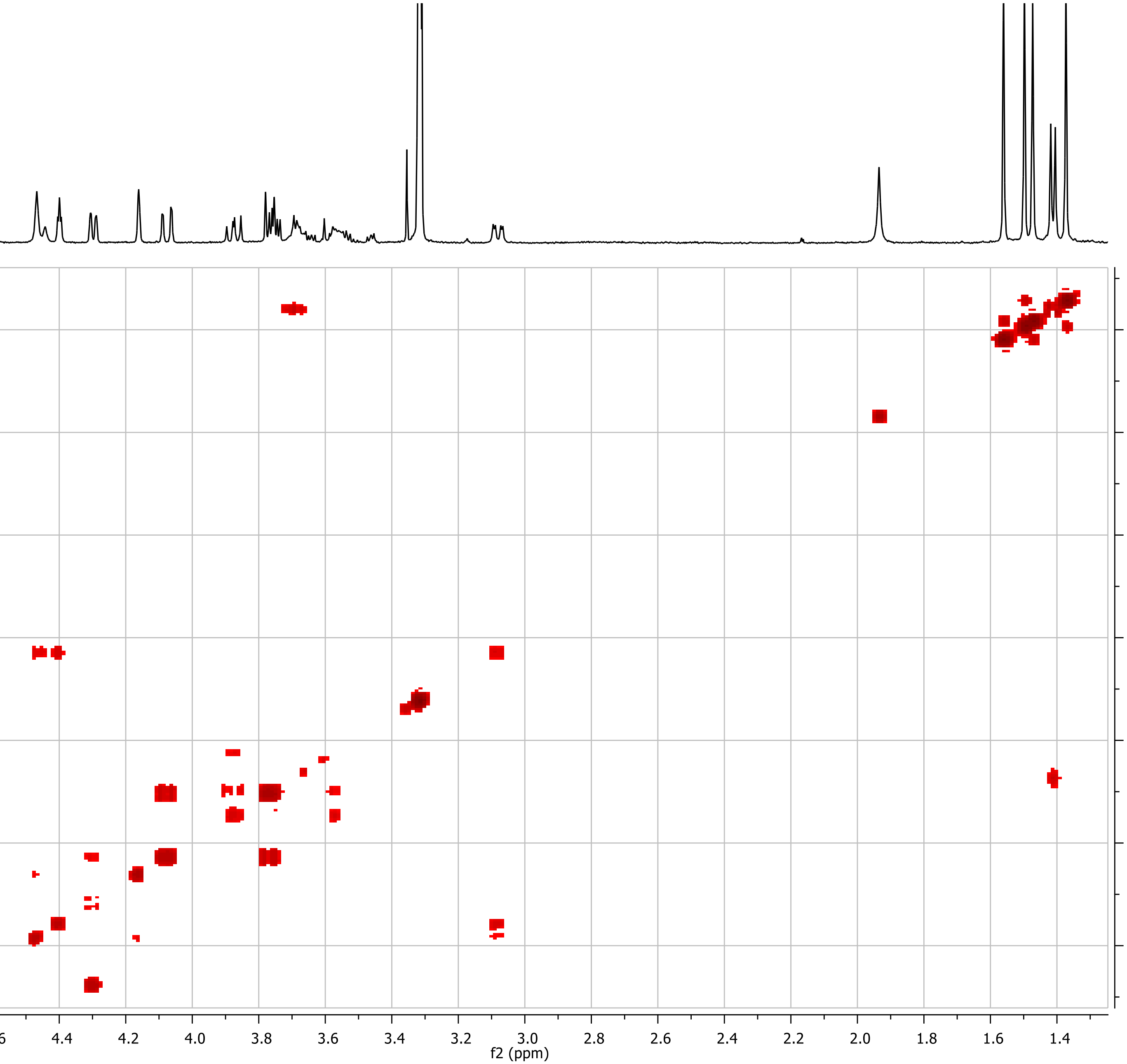

f2 (ppm)
f1 (ppm)
4.8
4.6
4.4
4.2
4.0
3.8
3.6
3.4
3.2
3.0
2.8
2.6
2.4
2.2
2.0
1.8
1.6
1.4
1.5
2.0
2.5
3.0
3.5
4.0
4.5

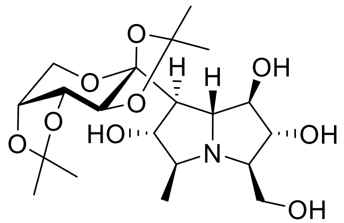

29

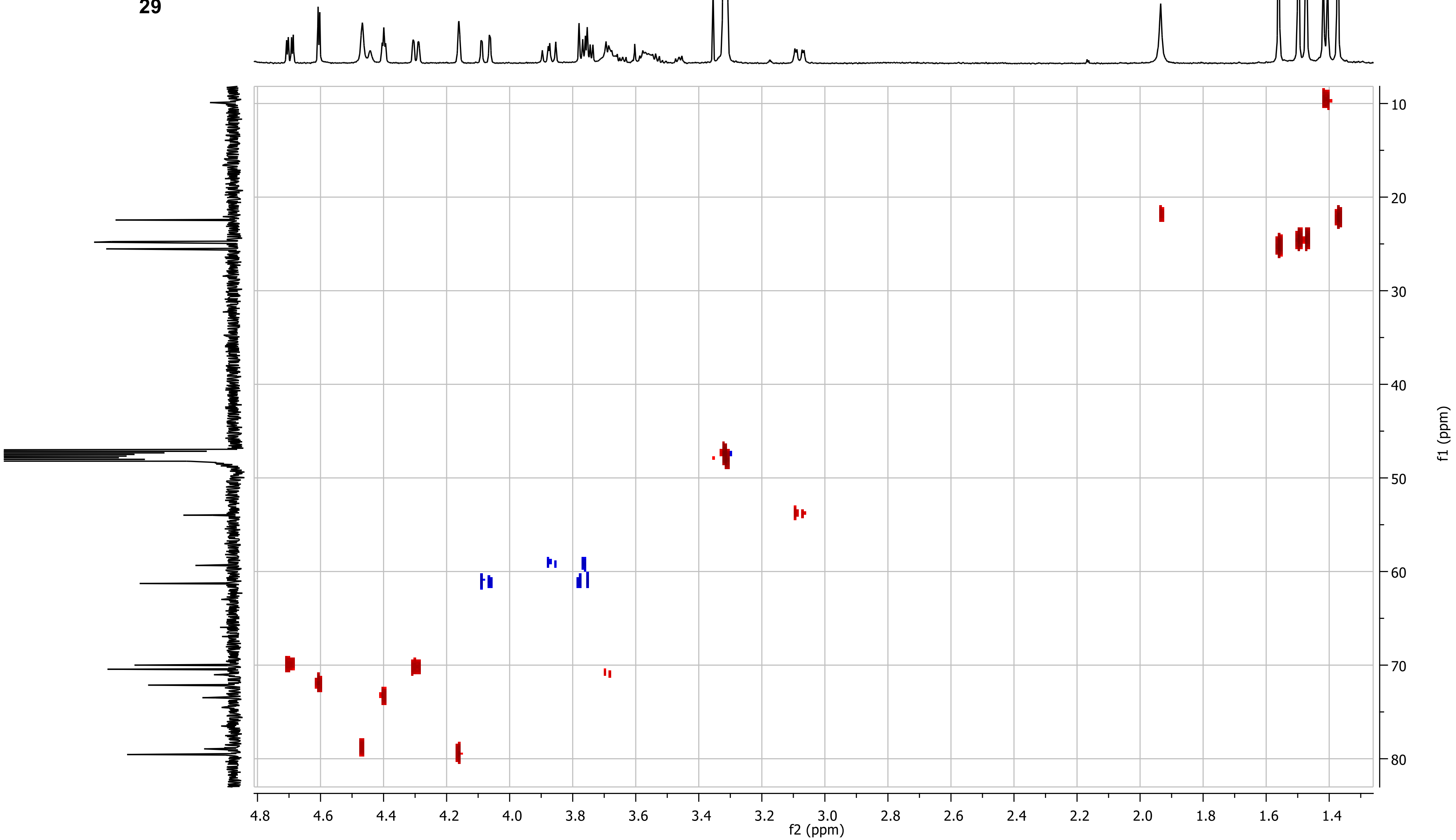

Supplement: Supplementary file 1 [file ao5c10431_si_001.pdf]
